# Mathematical Modelling of Parasite Dynamics: A Stochastic Simulation-Based Approach and Parameter Estimation via Modified Sequential-Type Approximate Bayesian Computation

**DOI:** 10.1007/s11538-024-01281-5

**Published:** 2024-04-10

**Authors:** Clement Twumasi, Joanne Cable, Andrey Pepelyshev

**Affiliations:** 1 Nuffield Department of Medicine, University of Oxford, South Parks Road, Oxford, Oxfordshire OX1 3SY UK; 2 School of Public Health, Imperial College London, 68 Wood Lane, London, Greater London W12 7RH UK; 3 School of Mathematics, Cardiff University, Senghennydd Road, Cardiff, South Glamorgan CF24 4AG UK; 4 School of Biosciences, Cardiff University, Sir Martin Evans Building, Cardiff, South Glamorgan CF10 3AX UK

**Keywords:** Individual-based model, Approximate Bayesian computation, Tau-leaping simulation, Host-parasite modelling, *Gyrodactylus*

## Abstract

The development of mathematical models for studying newly emerging and re-emerging infectious diseases has gained momentum due to global events. The gyrodactylid-fish system, like many host-parasite systems, serves as a valuable resource for ecological, evolutionary, and epidemiological investigations owing to its ease of experimental manipulation and long-term monitoring. Although this system has an existing individual-based model, it falls short in capturing information about species-specific microhabitat preferences and other biological details for different *Gyrodactylus* strains across diverse fish populations. This current study introduces a new individual-based stochastic simulation model that uses a hybrid $\tau$-leaping algorithm to incorporate this essential data, enhancing our understanding of the complexity of the gyrodactylid-fish system. We compare the infection dynamics of three gyrodactylid strains across three host populations. A modified sequential-type approximate Bayesian computation (ABC) method, based on sequential Monte Carlo and sequential importance sampling, is developed. Additionally, we establish two penalised local-linear regression methods (based on L1 and L2 regularisations) for ABC post-processing analysis to fit our model using existing empirical data. With the support of experimental data and the fitted mathematical model, we address open biological questions for the first time and propose directions for future studies on the gyrodactylid-fish system. The adaptability of the mathematical model extends beyond the gyrodactylid-fish system to other host-parasite systems. Furthermore, the modified ABC methodologies provide efficient calibration for other multi-parameter models characterised by a large set of correlated or independent summary statistics.

**Supplementary Information:**

The online version contains supplementary material available at 10.1007/s11538-024-01281-5.

## Introduction

### Background of the Study

Mathematical modelling and simulation play an increasingly crucial role in theoretical and applied ecology (Berec 2002). Applied mathematical models for host-parasite systems have evolved in response to the growing understanding of complex biological processes and the need for a more quantitative comprehension of such systems (Berec 2002; Grimm and Railsback 2005; Kaazempur-Mofrad et al. 2003; Twumasi et al. 2019). The use of individual-based modelling in population dynamics is a popular approach within contemporary theoretical ecology (Berec 2002), albeit its application in parasitological studies has been limited thus far (Gaba et al. 2006; Louie et al. 2007). This study builds upon our previous work (Twumasi et al. 2022), which focused on modelling a gyrodactylid-fish system to explore the spatial and temporal dynamics of two distinct co-infecting gyrodactylids (*Gyrodactylus turnbulli* and *G. bullatarudis*). Through re-analysing empirical data, our earlier study addressed three open biological questions related to this host-parasite system: microhabitat preferences of parasites, host survival, and parasite virulence over time. Twumasi et al. (2022) identified strain-specific microhabitat preferences, determined key factors influencing host survival, and quantified host-specific parasite virulence as a function of host mortality and recovery. However, the previous study did not incorporate spatial information and other relevant factors such as parasite fecundity, age group (young or old parasites), parasite mortality, parasite mobility, and host immune response. While a previous parasitological study developed an individual-based model (IBM) for this system (Oosterhout et al. 2008), their model lacks a comprehensive consideration of species-specific microhabitat preferences and other biological details for various *Gyrodactylus* strains across diverse fish populations (as discovered in Twumasi et al. (2022)). This highlights the need for a more robust and reproducible (individual-based) stochastic simulation model to address these gaps and enhance our understanding of the complex gyrodactylid-fish system.

In this current study, we present a new individual-based stochastic simulation model to explore the infrapopulation dynamics of a biological system over a standard 17-day experimental period. The model is designed to leverage the relative advantages of both IBMs and population-based models (PBMs). The infection dynamics of three different parasite strains are compared across three distinct fish populations over time. Based on a multi-dimensional continuous-time Markov chain (CTMC), our stochastic model employs a hybrid $\tau$-leaping simulation algorithm to enhance computational speed. Developed for the gyrodactylid-fish system, this simulation model aims to provide a relatively realistic representation of the biological system. Its goal is to facilitate the understanding of specific infection outcomes and address challenging experimental scenarios. The foundation of our simulator also rests on the mathematical and biological insights gained from our previously published study (Twumasi et al. 2022). A significant contribution of this current study is the provision of model-based statistical inferences for this system. For the first time, our study focuses on mathematically investigating: (i) gyrodactylids’ birth rates (for young and old parasites), (ii) species-specific death rates (in the presence or absence of an immune response), sex-specific mortality rates, (iii) host-specific immune response rates, (iv) species-specific movement rates, and (v) the effective parasite population carrying-capacity per fish host.

Approximate Bayesian computation (ABC) stands out as a widely-used likelihood-free estimation method, particularly in biological sciences and various fields, to fit complex models in simulation studies (Toni et al. 2009; Aryal and Jones 2020; Cisewski-Kehe et al. 2019; Corander et al. 2017; Christopher et al. 2021; Csilléry et al. 2012; McKinley et al. 2009; Wilkinson and Tavaré 2009). ABC methods find their application in modelling scenarios where the likelihood function of a model (Cox 2006) is either mathematically intractable or computationally expensive to evaluate. This approach approximates the true posterior distribution by summarising the data, often high-dimensional, using low-dimensional summary statistics. This simplifies the comparison between simulated and observed data, facilitated by a discrepancy distance measure (Li and Fearnhead 2018). The effectiveness of ABC hinges on the careful selection of summary statistics, a suitable distance metric, and the implementation of a Monte Carlo sampler (Li and Fearnhead 2018). Balancing the dimensionality of summary statistics is crucial; too many may distort the posterior approximation due to a low acceptance rate, while too few may result in a loss of data information (Prangle 2015). The quality of the posterior approximation is intricately tied to these choices. Beyond the basic ABC rejection algorithm (Pritchard et al. 1999), several improved versions have emerged, incorporating techniques like sequential Monte Carlo (SMC), Markov chain Monte Carlo (MCMC), sequential importance sampling (SIS), and regression-adjusted ABC samplers for posterior correction. These advancements aim to enhance computational efficiency, sample particles from regions of high posterior probability, ensure convergence to the true posterior, and broaden the applicability of ABC (Beaumont et al. 2002; Toni et al. 2009; Prangle 2015; Filippi et al. 2013). A substantial body of literature exists on ABC samplers, covering theoretical aspects of the resulting posterior distribution and its convergence (Twumasi 2022; Toni et al. 2009; Sisson et al. 2018; Filippi et al. 2013).

The current study introduces a modified ABC-SMC algorithm (dubbed weighted-iterative ABC), adapted from Filippi et al. (2013), and two penalised local-linear regression methods, utilising L1 and L2 regularisations. These additions aim to enhance the fitting of our model to high-dimensional empirical data. The penalised regression methods serve as robust extensions to the standard local-linear regression (proposed by Beaumont et al. (2002)) for ABC post-processing analysis. They address the imperfect match between simulated and observed data following ABC calibration, accommodating dependent or independent sets of summary statistics. For example, Beaumont et al.’s Beaumont et al. (2002) ABC posterior correction method faces implementation challenges due to matrix singularity issues arising from multicollinearity among simulated summaries in the neighbourhood of observed summaries or supercollinearity (when the number of ABC regression predictors exceeds the number of accepted ABC particles) (see Twumasi (2022), pp. 167–170). In this study, we also justify the necessity of our ABC regression-adjusted methods for other modelling problems by comparing high-dimensional simulated data using the unadjusted posterior (from the modified ABC-SMC sampler) and the corresponding adjusted posterior samples (based on our proposed penalised ABC correction methods) relative to the observed data within a reduced dimensional space. Finally, adopting a recently developing Bayesian hypothesis testing framework where the decision rule integrates estimated credible intervals and a region of practical equivalence, we address other open research questions of biological relevance based on the best-adjusted posterior samples following model identifiability and posterior predictive checks.

### Paper Structure, Research Questions and Study’s Limitations

This study is organised into four main sections. Section 1 summarises the study’s background, paper structure and research questions. In Sect. 2, we first describe the empirical data used in the study and then present our stochastic simulation model along with the proposed ABC methodologies. Under Sect. 3, we present the results of the ABC model fitting for our proposed simulation model based on both pseudo-observed (from our model) and observed experimental data (described in Sect. 2.1), respectively. Additionally, we include results from multivariate posterior predictive checks, employing Principal Component Analysis (PCA) and Principal Coordinate Analysis (PCoA), respectively. Bayesian hypothesis test results are also detailed. Finally, the concluding Sect. 4 presents discussions of main findings, conclusions, limitations, and recommendations for future works.

The study attempts to provide answers to the following five major research questions:

1. Are the birth rates (for young and old parasites) and death rates (with or without immune response) of *Gyrodactylus* parasites significantly different across the three parasite strains?
2. Is the adaptive immune response from gyrodactylid infection progression, host sex and host stock dependent?
3. Is the mortality rate of male fish with gyrodactylid infection significantly higher than female fish?
4. Are the microhabitat preferences of *Gyrodactylus turnbulli* and *G. bullatarudis* parasite species driven by their rate of movement on their fish host?
5. What is the effective population carrying capacity of *Gyrodactylus* parasites at the major body regions of their fish host?

## Methods

### Description of the Empirical Data

The observed parasite data utilised in the current study for model fitting were derived from the experimental investigation conducted by Cable and Oosterhout (2007). This dataset formed the basis for the IBM presented in Oosterhout et al. (2008) and the recent study on spatial-temporal parasite dynamics by Twumasi et al. (2022). To provide a brief overview, the experimental design involved 157 guppies in a full factorial design with nine distinct host-parasite combinations, comprising $13-22$ replicates per combination. Three different parasite strains were used to infect three different fish stocks: Ornamental Stock (OS), Lower Aripo River fish (LA), and Upper Aripo River fish (UA). However, five of the 157 guppies died before the observation or infection period, resulting in 152 guppy fish considered in the current study for model fitting, each with at least post-baseline data. The *Gyrodactylus* parasites included two strains of *Gyrodactylus turnbulli*: a laboratory-bred strain (*Gt3*) and a wild *turnbulli* strain (*Gt*). The second species was a wild-type strain of *G. bullatarudis* (*Gb*). Guppies were individually isolated, maintained under constant environmental conditions, and bred in a parasite-free environment, with tanks and containers arranged in a randomised block design. The total numbers of male and female guppy fish were 65 and 87, respectively. Each fish was initially infected with two parasites of the same strain, and parasite counts were recorded every two days (starting from day 1 after baseline infection) over a standard 17-day experimental period. The total number of parasites was recorded across eight distinct body regions (tail fin, lower body, upper body, anal fin, dorsal fin, pelvic fins, pectoral fins, and head) for each fish host. Additional laboratory experiments collected data on the surface area of each of the eight body regions of some selected guppy populations (and recorded across fish sex and fish stock).

### Proposed Stochastic Simulation Model

#### Introduction

Before formally defining our new stochastic simulation model for the gyrodactylid-fish system, we present a rationale for adopting a continuous-time Markov Chain (CTMC) and outline additional biological motivations influencing modelling considerations. Now, CTMCs are commonly used for modelling biological systems with low population counts and high uncertainty in state transitions (Banks et al. 2012). While gyrodactylid mean intensities are generally low in guppy populations; infection dynamics vary across parasite strains and fish populations (Twumasi et al. 2022). Therefore, a CTMC simulation model for the gyrodactylid-fish system can capture its stochastic nature and incorporate complexities given the empirical data.

Due to the hyperviviparous nature of *Gyrodactylus* parasites, they give birth to fully grown and pregnant young parasites. This reproductive process can rapidly increase the population or induce infections in their host within a short period (Bakke et al. 2007). Consequently, in the current study, we differentiate between young and old parasites in our simulation model, considering a mother as old and a newly born parasite as young before conception. In addition, as parasite numbers increase at the body region of a host, an immune response can be produced as the infection progresses, with non-response for some fish hosts. Hence, immune response is also considered as another realistic event in our simulation model (which may or may not occur for some fish). The formal mathematical definition of the new simulation model for the gyrodactylid-fish system is presented in Sect. 2.2.2.

#### Model Framework

**Fig. 1:**
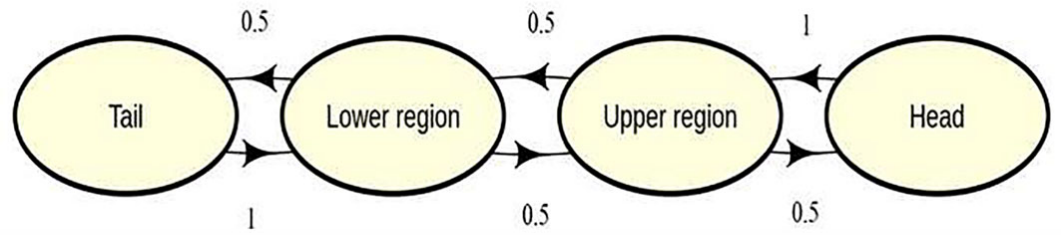
Transition diagram across the four major body regions of fish used as states for the CTMC model for a single parasite

The model simulates the movement of parasites, conditioned on relevant information such as fish sex, fish size, fish stock, and parasite strain, for two age groups (young and old parasites) over the external surfaces (i.e., four major body regions as recommended by Twumasi et al. (2022)) of a fish throughout a 17-day infection period. The population carrying capacity depends on the host size and the area of body regions. Figure 1 illustrates the four major body locations: tail, lower region (comprising the lower body, anal fin, pelvic fins, and dorsal fin), upper region (composed of the upper body and pectoral fins), and the head for a single host in the stochastic model.

The model is parameterised by the birth, death, and movement rates of young and older parasites, considering the presence or absence of the host’s immune response. Host death is assumed to occur at a rate proportional to the total number of parasites on the fish. Additionally, the stochastic model incorporates parasite body preference, which depends on the parasite strain (microhabitat preference). Model parameters also include the preference for parasites to move back and forth on the host and the effective carrying capacity (total parasites that can occupy a body location). It is essential to acknowledge that the omission of any time index *t* in a time-dependent quantity in certain instances (in the subsequent model description framework) is motivated by the desire for simplicity, notwithstanding that we assume the process exhibits time homogeneity. However, it is crucial to emphasise that, despite this simplification, the system maintains its dynamic and time-dependent nature (where applicable).

Now, suppose individual gyrodactylid parasite on a fish can transition between the four discrete states or major body locations: tail (state 1), lower region (state 2), upper region (state 3) and head (state 4) as represented by the transition diagram (Fig. 1). For a single fish, let $\{A_{j,k}(t); t\ge 0 \}$ be $4\times 2$ matrix denoting the number of gyrodactylid parasites at body location *j* ($j=1,2,3,4$) per parasite age group *k* ($k=1,2$) at any time *t*; where $k=1$ represent young parasites (daughter yet to reproduce) and $k=2$ denote old parasite (mother). Let $\{X_{j}(t); t\ge 0 \}$ be the total number of young and old gyrodactylid parasites at any time *t* at the *j*th body location of a fish from any parasite-fish group (i.e., *Gt3*-OS, *Gt3*-LA, *Gt3*-UA, *Gt*-OS, *Gt*-LA, *Gt*-UA, *Gb*-OS, *Gb*-LA or *Gb*-UA); such that $X_{j}(t)=\sum \limits _{k=1}^{2}A_{j,k}(t)$ for $t \in [t_{u-1},t_u)$ (where $u=1,2,\cdots ,9$ are observed time indices). For simplicity, let $X_{j}(t)=X_j$; then for each fish, we have observations $X_{j}=\left\{ X_{j0}, X_{j1}, \cdots , X_{j9}\right\}$ at times $t_0=0$, $t_1=1$, $t_2=3$, $\cdots$, $t_9=17$.

Let $z_h=\{z_{h1},z_{h2},z_{h3}\}$ be the respective realised values of the covariates: fish sex, fish size and fish stock, for fish *h*; where $h=1,2, \cdots , n_l$ with $n_l$ denoting the total number of parasites in the *l*th parasite-fish group (with $1 \le l \le 9$). Let also assume that $I_j(t)=I_j \rightarrow \{0, 1 \}$ is an (unobserved) indicator function representing the immune state of the *j*th body region of a host at time *t*; such that 0 indicates the absence of immune response, while 1 implies the presence of immune response. To generalise for all fish, let suppose that $\{A_{j,k}^{(h)}(t); t\ge 0 \}$ is a multidimensional time-homogeneous Markov chain, and $S^{(h)}(t)=S_t^{(h)}$ denotes its state vector at time *t* for the *h*th fish. Assuming *K* parasite age groups (a total of 2), *J* body regions (a total of 4), *I* immune states (a total of 2), and *W* host mortality states (a total of 2), then the state space $S_t^{(h)}$ is defined as a multidimensional vector with $J \times K \times I \times W$ components, denoted as $S_t^{(h)}= \left( s_{t, j, k, i, w}^{(h)} \right)$ for $1 \le j \le J$, $1 \le k \le K$, $1 \le i \le I$, $1 \le w \le W$; where $s_{t, j, k, i, w}^{(h)}$ represents the number of parasites per age group *k* at body region *j* with immune state *i* and host mortality state *w* for fish *h*.

For an individual fish *h* at any time *t*, we therefore assume that $A_{j,k}^{(h)}(t)=A_{j,k}^{(h)}$ satisfies the stochastic scheme defined by Table 1; where $b_{gk}(I_{j})$ is the birth rate for the *g*th parasite strain (for $1 \le g \le 3$) aged $1 \le k \le 2$ (which depends on the immune state $I_j$ at body region *j*), $d_{gi}$ is the death rate for *g*th strain at immune state $0 \le i \le 1$, $m(I_j)$ is a parasite’s movement rate (which depends on $I_j$), $\epsilon _{g}$ is the movement rate adjustment for the *g*th strain, $r(z_{h1},z_{h3})$ is the immune response rate by a single parasite (which depends the fish sex $z_{h1}$ and fish stock $z_{h3}$), $s(z_{h1},z_{h2})$ is the fish mortality rate caused by a single parasite (which depends on the fish sex $z_{h1}$ and fish size $z_{h2}$), $\xi (f_j,z_{h2},\kappa )$ is the population carrying capacity (which depends on the area of body region $f_j$, fish size $z_{h2}$ and the effective carrying capacity per unit area of each body region, $\kappa$). The main model parameters of underlying the stochastic simulation to be estimated are described in Table 2.

**Table 1:** The modelling scheme of the CTMC stochastic simulation at any time

Event	Transition	Rate
Parasite birth at region *j*	$A_{j,k}^{(h)} \rightarrow A_{j,k}^{(h)}+1$	$A_{j,k}^{(h)} \times \left[ 1-\frac{A_{j,k}^{(h)}}{\xi (f_j,z_{h2},\kappa )}\right] \times b_{gk}(I_{j})$
Parasite death at region *j*	$A_{j,k}^{(h)} \rightarrow A_{j,k}^{(h)}-1$	$A_{j,k}^{(h)} \times \left[ 1-\frac{A_{j,k}^{(h)}}{\xi (f_j,z_{h2},\kappa )}\right] \times d_{gi}$
Forward movement from region *j* to $j+1$	$A_{j,k}^{(h)} \rightarrow A_{j,k}^{(h)}-1$	$A_{j,k}^{(h)} \times m(I_{j})\times \epsilon _g$
	$$A_{j+1,k}^{(h)} \rightarrow A_{j+1,k}^{(h)}+1$$	
Backward movement from region *j* to $j-1$	$A_{j-1,k}^{(h)} \rightarrow A_{j-1,k}^{(h)}+1$	$A_{j,k}^{(h)} \times m(I_{j})\times (1-\epsilon _g)$
	$$A_{j,k}^{(h)} \rightarrow A_{j,k}^{(h)}-1$$	
Immune response at region *j*	$\sum \limits _{k=1}^{2}A_{j,k}^{(h)} \rightarrow 0$	$\left[ \sum \limits _{k=1}^{2}A_{j,k}^{(h)}\right] \times r(z_{h1},z_{h3})$
Mortality of host *h*	$\sum \limits _{j=1}^{4} \sum \limits _{k=1}^{2} A_{j,k}^{(h)} \rightarrow 0$	$\left[ \sum \limits _{j=1}^{4} \sum \limits _{k=1}^{2}A_{j,k}^{(h)}\right] \times s(z_{h1},z_{h2})$

**Table 2:** Main model parameters of the CTMC stochastic simulation

Parameters	Description
*Base simulation parameters*	
$b_{11}$	Birth rate for young *Gt3* parasites
$b_{12}$	Birth rate for old *Gt3* parasites
$b_{21}$	Birth rate for young *Gt* parasites
$b_{22}$	Birth rate for old *Gt* parasites
$b_{31}$	Birth rate for young *Gb* parasites
$b_{32}$	Birth rate for old *Gb* parasites
$d_{11}$	Death rate for *Gt3* parasites without host immune response
$d_{12}$	Death rate for *Gt3* parasites with host immune response
$d_{21}$	Death rate for *Gt* parasites without host immune response
$d_{22}$	Death rate for *Gt* parasites with host immune response
$d_{31}$	Death rate for *Gb* parasites without host immune response
$d_{32}$	Death rate for *Gb* parasites with host immune response
*m*	Movement rate for a single parasite
*r*	Immune response rate caused by a single parasite
*s*	Host mortality rate caused by a single parasite
$\kappa$	Effective carrying capacity per each body region
*Additional simulation parameters*	
$\epsilon _1$	Movement rate adjustment for *Gt3* parasites
$\epsilon _2$	Movement rate adjustment for *Gt* parasites
$\epsilon _3$	Movement rate adjustment for *Gb* parasites
$r_1$	Immune response rate adjustment for LA fish (ref: UA fish)
$r_2$	Immune response rate adjustment for OS fish (ref: UA fish)
$r_3$	Immune response rate adjustment for male fish (ref: female)
$s_1$	Host mortality rate adjustment for male fish (ref: female)

The probability that a single parasite will move between the four major body regions of fish within the simulation model ($J_{\text {transition}}$) is assumed to be constant over time (as shown in Fig. 1), and it is given asThe specific underlying assumptions of the CTMC simulation model are as follows:

1. The birth rate of young parasites are greater than the old parasites’ birth rate.
2. The death rate of young and old parasites are assumed to be equal but higher in the presence of host immune response.
3. The birth rate per age as well as the death rate with or without host immune response depend on the parasite strain.
4. Host mortality occurs at a rate proportional to the total number of parasites on the body of the fish, fish sex and fish size.
5. The rate of movement of each parasite depends its age, strain and host immune response.
6. Localised host immune response at each body region occurs at a rate proportional to the effective population carrying capacity per unit area, fish sex and fish stock. The localised immune response can also occur at any time within the observed infection period.
7. The fish size is measured by its standard length, and the unit area of the host’s body regions depends on its size and sex.
8. The population carrying capacity depends on the unit area of the host’s body regions, fish size and the effective carrying capacity (maximum number of parasites per unit area of body regions).
9. The transition or event rates are time-homogeneous and dependent on the current state of the process (independent of past states) within any infinitesimal amount of time or time step of the $\tau$-leaping simulation.

### Hybrid $\tau$-Leaping Algorithm for the Multidimensional CTMC Simulation Model

The CTMC stochastic simulation model is developed using a hybrid $\tau$-leaping algorithm whose leap size, $\tau _{\text {leap}}$, is given by Eq. 1 (adapted from Twumasi (2022), pp. 129–146); such that

1 $$\begin{aligned} \tau _{\text {leap}}= \min \left\{ \frac{\epsilon ({\bar{b}}+{\bar{d}})}{|({\bar{b}}- {\bar{d}})| \max ({\bar{b}},{\bar{d}})}, \frac{\epsilon ^2({\bar{b}}+{\bar{d}})^{2} \left[ \sum \limits _{j=1}^{4} \sum \limits _{k=1}^{2}A_{j,k}^{(h)}\right] }{({\bar{b}}+{\bar{d}})\max ({\bar{b}}^2,{\bar{d}}^2)} \right\} , \end{aligned}$$

where ${\bar{b}}$ is the average birth rate of young and old parasites, ${\bar{d}}$ is the average death rate of parasites in the presence or absence of host immune response, and $\epsilon$ is the error bound of the $\tau$-leaping algorithm (set at $\epsilon =0.002$; see Supplementary S2 in Additional supplementary material). The leap condition is determined by $\frac{1}{10a_0\left( A_{j,k}^{(h)}\right) }$ where $a_0\left( A_{j,k}^{(h)}\right)$ is the total event rate (which depends on state $A_{j,k}^{(h)}$) for fish *h* as specified in (Twumasi (2022), p. 134). Thus, the hybrid $\tau$-leaping is set up such that if the leap size $\tau _{\text {leap}}$ (given by Eq. 1) > $\frac{1}{10a_0\left( A_{j,k}^{(h}\right) }$, the $\tau$-leaping algorithm is implemented for a single fish, whereas we forego $\tau$-leaping and use the exact stochastic simulation algorithm (SSA) when the leap condition is not met. The hybrid $\tau$-leaping simulation at an error bound of 0 ($\epsilon =0$) result in exact SSA only since at $\epsilon =0$ (Gillespie 2001; Gillespie and Petzold 2003), the leap size $\tau _{\text {leap}}=0$ for any state value and birth-death parameter values $>0$. The pseudo-codes for the exact SSA and the hybrid $\tau$-leaping simulation algorithm are presented under Supplementary S1 in Additional supplementary material.

### Weighted-Iterative ABC

#### Introduction

As briefly highlighted in Sect. 1, ABC typically reduce high-dimensional data to low-dimensional user-chosen summary statistics and accept samples of the model parameter $\theta \in {\mathbb {R}}^n$ when the simulated summaries $s_{\text {sim}}=S(y_{\text {sim}})$ are close to the observed summaries $s_{\text {obs}}=S(y_{\text {obs}})$ such that $\rho \left( s_{\text {sim}}, s_{\text {obs}} \right) \le \epsilon$ for sufficiently small pre-defined tolerance level $\epsilon >0$; where $S(\cdot ) \in {\mathbb {R}}^m$ is the summary statistics of the data (possibly *m*-dimensional), $y_{\text {sim}} \sim f( \cdot \mid \theta )$, and $\rho (\cdot )$ is a discrepancy measure (e.g., Euclidean distance). Jung and Marjoram (2011) demonstrated that assigning higher weights to more informative summaries, as part of a well-chosen tolerance in ABC analysis, tremendously enhances performance compared to unweighted analysis. Additionally, in the literature, sequential Monte Carlo ABC (ABC-SMC) samplers have been proposed to address certain shortcomings linked with rejection-based ABC and ABC-MCMC samplers (such as particle degeneration and sampling from regions with lower posterior probability).

The ABC algorithm developed in the current study is a modification of the ABC-SMC sampler described in Filippi et al. (2013). In our modified ABC-SMC algorithm, we introduce a weighting scheme for the set of summary statistics per host to extract relevant information from high-dimensional parasite population data. For a single simulation run, our stochastic model generates high-dimensional data or a set of *M* sample paths over time and space (across the host’s body regions), corresponding to the entire observed fish with a population size of $M=152$. The set of carefully chosen summary statistics computed for a given host data includes: (i) log count of parasites across observed times (**9** summaries), (ii) Wasserstein $1-D$ distance between parasite distributions at host’s body regions (**4** summaries), (iii) the time before death (**1** summary), and (iv) parameter estimates of the birth-death process with catastrophic extinction (B-D-C process) based on all simulated sample paths (**3** summaries). The concept and detailed theoretical works on the B-D-C process and its parameter estimation can be found in work by Twumasi (2022, pp. 95–126). The motivation for refining the ABC summaries using the B-D-C parameter estimates is that this process simplifies our complex simulation model as a linear birth-death process where the process is subjected to catastrophes (e.g., host mortality) that result in parasite population extinction-an important phenomenon observable in the empirical data.

In a single simulation run, a matrix with a dimension of $152 \times 17$ summary statistics is obtained for comparing the discrepancy between the simulated and observed data during the ABC fitting of our stochastic model. The discrepancy metric is considered a weighted sum of squares distance metric $\rho$, extending the standard weighted Euclidean distance. An optimised linear regression function is developed (and presented under Supplementary S3 in Additional supplementary material) to aid in computing the summary statistics during ABC fitting after premature host mortality by projecting the infrapopulation of parasites till the end of the infection period.

#### Description of the Modified ABC Algorithm

Algorithm 1 is the pseudo-code for the weighted-iterative ABC algorithm. The main modifications in Algorithm 1 with respect to the previous ABC-SMC Algorithm defined in Filippi et al. (2013) are: (i) adaptively integrating importance weights for importance proposal sampling and summary statistics weights (based on accepted simulations by computing the harmonic mean between previous and current summary statistics weights at time $t \ge 1$) to improve ABC posterior approximations, (ii) inclusion of a weighted distance metric for comparing between multidimensional data of an entire population (in the case where summary statistics has bi-dimensional space), (iii) adaptation of a computationally efficient multivariate normal perturbation kernel with bandwidth matrix optimally determined, and (iv) an independent *post-hoc* step which entails a robust correction method to adjust the resulting ABC posterior approximation using a penalised heteroscedastic local-linear regression. The steps of the modified ABC algorithm can briefly be explained as follows:

- Suppose we have a decreasing sequence of tolerances $\epsilon _1> \epsilon _2> \cdots > \epsilon _T$ (*T* being the final time step), the prior distribution $\pi ( \cdot )$, a simulation model given by $f( \cdot \mid \theta )$, and a observed summary statistics $s_{\text {obs}}$ (possibly multidimensional).
- At time $t=1$, the weighted-iterative ABC algorithm draws proposals $\theta _i^{(1)} \sim \pi (\theta )$ (for $1 \le i \le N$) from the prior distribution $\pi (\theta )$ with equal importance weight of $W_i^{(1)}= \frac{1}{N}$; the accepted particles at the largest tolerance ($\epsilon _1 \le 1$) is indicated as $p_{\epsilon _1}(\theta \mid s_{\text {obs}})$ (or $p_{\epsilon _1}$ for simplicity), and considered as the first intermediate prior distribution. The initial distribution of $\pi (\theta )$ was determined for $\theta$ in the current study based on flat non-informative uniform priors (on a logarithmic scale) at $t=1$. Instead of commencing the rejection sampling with a smaller tolerance (as in the case of the standard rejection-based samplers), at $t=1$, the algorithm is similar to the standard rejection ABC (but with a larger tolerance comparatively). The discrepancy between simulated and observed summary statistics, given $\theta _i^{(t)}$ at time $t \ge 1$, is computed using the scaled weighted sum of squares distance metric such that
  2 $$\begin{aligned} \rho \left( s_{\text {sim}},s_{\text {obs}} \right) = \sqrt{\frac{1}{M} \sum _{k=1}^{{\textbf{M}}} \sum _{j=1}^{m} {\textbf{w}}_{j}^{(t)}\left( s_{\text {sim}_{k,j}}- s_{\text {obs}_{k,j}} \right) ^2}, \quad 1 \le t \le T. \end{aligned}$$
  where *M* is the total population size, *m* is the summary statistics length per simulation sample path or host ($m=17$ in our case), ${\textbf{w}}^{(t)}$ is a vector of the summary statistics weights at time *t*, and our summary statistics is assumed to have a bi-dimensional space (for a one-dimensional summary statistics, the standard weighted Euclidean distance can be used as the discrepancy measure instead). Prior to computing the weighted sum of squares distance metric $\rho \left( \cdot \right)$, the summary statistics weight ${\textbf{w}}^{(t)}$ at time $t \ge 1$, is computed based on the harmonic mean of the current weight $w_{j^ \prime }^{(t)}=1/ \sigma _{j^ \prime }^2$ (based on accepted particles, where $\sigma _{j^ \prime }$ is the standard deviation of the $j^ \prime$th summary statistic) for $1 \le j,j^ \prime \le m$ and the previous weight ${\textbf{w}}^{(t-1)}$; such that
  $$\begin{aligned} w_j^{(t)}= \frac{2}{ \frac{1}{w_j^{(t-1)}}+\frac{1}{w_{j^ \prime }^{(t)}}}. \end{aligned}$$
  According to Prangle (2017), there is no assurance that the summary statistics weights ${\textbf{w}}^{(t)}$ (meant to normalise the summary statistics at time step $t \ge 1$ for iterative ABC such as ABC-SMC) would actually normalise the summary statistics at subsequent iterations since particles or proposals are not sampled directly from the prior $\pi (\theta )$, but instead, from different proposal distributions $g_t(\theta )$ over time $t \ge 1$. Hence, the main motivations for adopting the harmonic mean of the previous and current summary statistics weights (based on the multiplicative inverse of the variance of the *j*th summary statistic of accepted particles) in this study (instead of strictly using the conventional approaches defined in Prangle (2017)) are to (i) minimise the degree of variability in the high-dimensional summary statistics weights at time $t \ge 1$ (based on averages across the entire host population as observed in the current study), and (ii) control the potential high disparities between the summary statistics weights at the current ABC time step *t* and the previous time $t-1$ as well as improve normalisation of summary statistics weights due to direct particle sampling from different proposal distributions (at ABC time steps $t-1$ and *t*) instead of the (initial) prior.
- At $t \ge 2$, the algorithm works in steps (with $\epsilon _t < \epsilon _{t-1}$): instead of directly sampling from $\pi (\theta )$, we randomly draw weighted particles $\theta ^* \sim p_{\epsilon _{t-1}}$ (for *N* different times) from the current intermediate prior $p_{\epsilon _{t-1}}$ with a probability equal to their corresponding normalised importance weight $W_i^{(t)}$ (estimated from Eq. 5). Following Filippi et al. (2013), we then perturb particles $\theta _i^{(t)} \sim K_{H^{(t)}}(\cdot \mid \theta ^*)$ at iterations $t \ge 2$ using a multivariate normal (MVN) perturbation kernel $K_{H^{(t)}}$ centred at or near $\theta ^*$, such that
  3 $$\begin{aligned} K_{H^{(t)}} \left( \theta ^{(t)} \mid \theta ^* \right)= & {} \frac{1}{ \sqrt{(2 \pi )^n \left( \det {H^{(t)}} \right) }}\nonumber \\ {}{} & {} \exp \left\{ -\frac{1}{2} \left( \theta ^{(t)}-\theta ^* \right) ^ {\top } \left( H^{(t)} \right) ^{-1} \left( \theta ^{(t)}-\theta ^* \right) \right\} , \end{aligned}$$
  with an optimal bandwidth matrix
  4 $$\begin{aligned} H^{(t)}= \sum \limits _{i=1}^N \sum \limits _{k=1}^{N_{\epsilon _{t-1}}} W_i^{(t-1)} {\tilde{W}}_k \left( {\tilde{\theta }}_k-\theta _i^{(t-1)} \right) \left( {\tilde{\theta }}_k-\theta _i^{(t-1)} \right) ^ {\top }; \end{aligned}$$
  where the quantity $\left\{ {\tilde{\theta }}_k \right\} _{1 \le k \le N_{\epsilon _{t-1}}}$ denote the set of accepted particles
  $\left\{ \theta _i^{(t-1)} \text {s.t.} \quad \rho (s_{\text {sim}},s_{\text {obs}}) \le \epsilon _{t}, \quad 1 \le i \le N \right\}$, with their corresponding importance weight $\left\{ {\tilde{W}}_k \right\} _{1 \le k \le N_{\epsilon _{t-1}}}$ normalised over all $1 \le k \le N_{\epsilon _{t-1}}$. Filippi et al. (2013) have shown that this choice of kernel bandwidth has good theoretical properties. We then simulate data $y_{\text {sim}} \sim f(\cdot \mid \theta _i^{(t)})$ for $1 \le i \le N$, obtain $N_{\epsilon _{t}}$ accepted samples $p_{\epsilon _{t}}(\theta \mid s_{\text {obs}})$ accordingly, and repeat the process until we reach the final or target posterior $p_{\epsilon _{T}}(\theta \mid s_{\text {obs}})$ at the final time step $t=T$ (where $N \ge N_{\epsilon _{1}}> N_{\epsilon _{2}}> \cdots >N_{\epsilon _{T}}$). Here,
  5 $$\begin{aligned} W_i^{(t)} = \frac{ \pi \left( \theta _i^{(t)} \right) }{\sum \limits _{l=1}^{N} W_l^{(t-1)} K_{H^{(t)}} \left( \theta _i^{(t)} \mid \theta _l^{(t-1)} \right) }, \quad 2 \le t \le T \quad \text {and} \quad W_i^{(1)}=\frac{1}{N}. \end{aligned}$$
- At time $t=1$, the initial prior density $\pi (\theta ) \propto g_1(\theta )$ is considered as the first importance or proposal density $g_1(\theta )$; whereas at $t \ge 2$, the importance or proposal density $g_t(\theta )$ is derived from Eq. 6 such that
  6 $$\begin{aligned} g_t(\theta )= \sum \limits _{i=1}^{N} W_i^{(t-1)} K_{H^{(t)}} \left( \theta \mid \theta _i^{(t-1)} \right) / \sum \limits _{i=1}^{N} W_i^{(t-1)}. \end{aligned}$$

Finally, we adjust the approximate posterior distribution, denoted as $p_{\epsilon _{T}}(\theta \mid s_{\text {obs}})$ at time $t=T$, obtained from the weighted-iterative ABC method. This adjustment is accomplished using a robust regression method with *L*1 and *L*2 regularisations, as proposed in Sect. 2.5. It is worth noting that the original local-linear regression methods by Beaumont et al. (2002) are non-implementable in multicollinear scenarios due to matrix singularity issues. It can be inferred from Prangle (2017) theoretical work on ABC-SMC convergence that as $t \rightarrow \infty$ and $\epsilon _t \rightarrow 0$, Algorithm 1 draws approximate samples from the ABC posterior with density

$$\begin{aligned} p_{\epsilon _t}(\theta \mid s_{\text {obs}})=\int \left[ { f(s_{\text {sim}} \mid \theta ) \pi (\theta ) \mathbb {1}_{A_{\epsilon _t,s_{\text {obs}}}} }/{ \int _{{\mathbb {R}}^n \times {\mathbb {R}}^m} f(s_{\text {sim}} \mid \theta )\pi (\theta ) \mathbb {1}_{A_{\epsilon _t,s_{\text {obs}}}} d \theta ds_{\text {sim}}} \right] ds_{\text {sim}}, \end{aligned}$$

where $\theta \sim g_t(\theta )$ and $\mathbb {1}_{A_{\epsilon _t,s_{\text {obs}}}}(\cdot ) \rightarrow \{0, 1 \}$ is an indicator function of the Lebesgue-measurable set ${A_{\epsilon _t,s_{\text {obs}}}}=\{s_{\text {sim}} \mid \rho (s_{\text {sim}},s_{\text {obs}} ) \le \epsilon _t \}$; whereas $\rho (\cdot )$ and $W^{(t)} \propto \frac{\pi (\theta )}{g_t(\theta )}$ are defined by Eqs. 2 and 5, respectively. Our modified ABC algorithm is set-up to have a fixed number of iterations or time steps (i.e., a total of 10 time steps), and a set of monotonically decreasing tolerances ($\epsilon _t$, $t=1, 2, \cdots ,10$) at each ABC time step *t* is carefully pre-specified such that: $\epsilon _t= 0.5$, 0.43, 0.4, 0.35, 0.3, 0.2, 0.1, 0.08, 0.06, 0.02.

**Algorithm 1:**
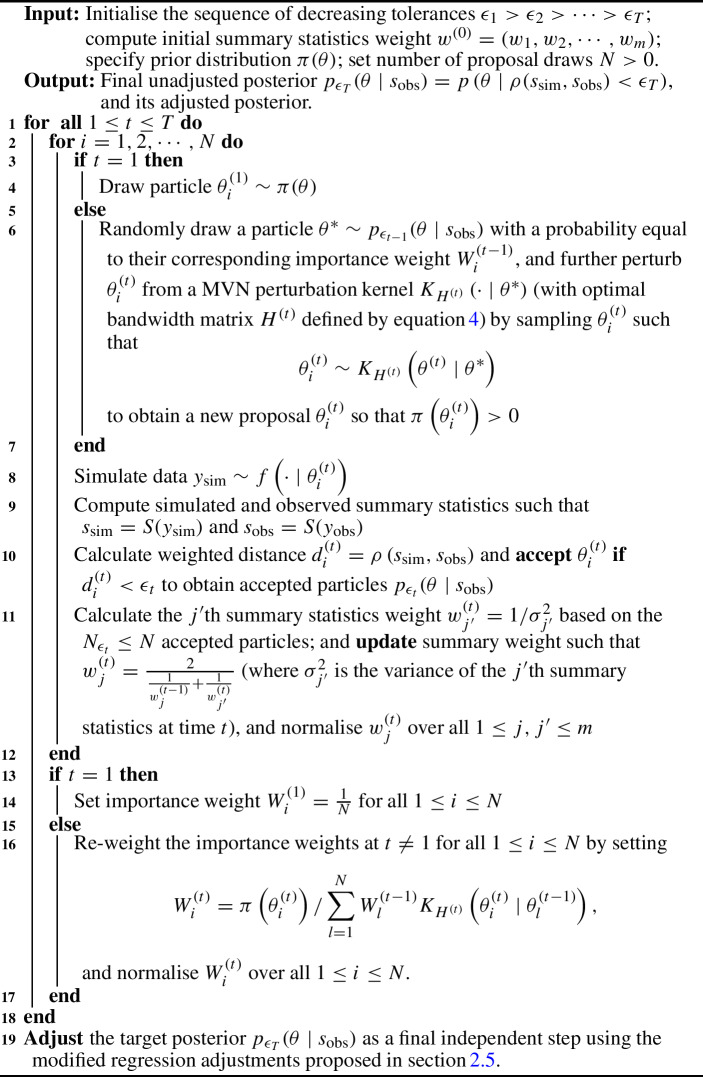
Pseudo-code of the weighted-iterative ABC

### Weighted Ridge and Lasso Regressions for Posterior Adjustment

This section describes our two penalised regression adjustment methods: weighted ridge regression (WRR) and weighted lasso regression (WLR). The work of Arkin and Montgomery (1980) inspired these penalised regression approaches, who originally developed WRR for general regression problems unrelated to ABC. In our proposed regression-adjusted methods, the dependent variables represent the posterior samples or approximate posterior distribution of the model parameters (on a logarithmic scale) obtained from the modified ABC-SMC algorithm. The predictors are the corresponding simulated summary statistics in the vicinity of the observed summaries. To streamline the regression adjustment, we transform the two-dimensional simulated summary statistics ($s_{\text {sim}}$) across all 152 fish into a one-dimensional format within the neighbourhood of $s_{\text {obs}}$ before applying regression. After fitting our simulation model, the primary objective of regression adjustments is to enhance the resulting posterior samples.

#### Proposed ABC Posterior Mean Adjustment

Given a set of $\eta$ unadjusted posterior samples from the weighted-iterative ABC algorithm (described by Algorithm 1), let $\theta _i^{(r)}$ be the *i*th posterior sample (for $i=1,2,\cdots , \eta$) for the *r*th model parameter (for $r=1,2,\cdots , n$). Suppose $s_{\text {sim},i}$ are the accepted simulated summary statistics (with dimension $M \times m$) corresponding to the *i*th posterior sample; where the $M \ge 1$ corresponds to a population size, and $m \ge 1$ the number of summary statistics for each individual in the population model (to be simulated). The regression model in the vicinity of the observed summary statistics $s_{\text {obs}}$ (with dimension $M \times m$) is given as

7 $$\begin{aligned} \theta _i^{(r)}= \alpha ^{(r)} + \bar{{\mathcal {S}}}_i^\top \beta ^{(r)}+ \varsigma _i^{(r)}, \quad 1 \le i \le \eta \quad \text {and} \quad 1 \le r \le n \end{aligned}$$

where $\bar{{\mathcal {S}}}_i= \frac{1}{M}\sum \limits _{k=1}^{M} \left[ s_{\text {sim}_{{(k,m)}},i}-s_{\text {obs}_{(k,m)}} \right]$ is an *m*-dimensional vector of mean differences between $s_{\text {sim},i}$ and $s_{\text {obs}}$ across all *M* individuals for the *i*th posterior sample; $\alpha ^{(r)}$ is the intercept (whose estimate represent the required adjusted posterior mean), $\beta ^{(r)}$ is a vector of regression coefficients corresponding to the *m* predictors (in the neighbourhood of $s_{\text {obs}}$), and $\varsigma _i^{(r)}$ are the regression error terms with mean 0 and heteroscedastic variance, corresponding to the *r*th model parameter. If $M=1$, $\bar{{\mathcal {S}}}_i= s_{\text {sim},i}-s_{\text {obs}}$ as in the case of Beaumont et al. (2002) regression adjustment methods (where $s_{\text {sim},i}$ and $s_{\text {obs}}$ are assumed to a one-dimensional array or vector of length *m*, respectively).

Given Eq. 7, the robust weighted ridge regression estimates of $\left( \alpha ^{(r)},\beta ^{(r)} \right)$ can be derived by minimising the loss function ${\mathcal {L}}_{\text {ridge}}^{(r)}$ for each *r*th model parameter such that

8 $$\begin{aligned} {\mathcal {L}}_{\text {ridge}}^{(r)}=\sum _{i=1}^{\eta } \left\{ \theta _i^{(r)} - \alpha ^{(r)} - \sum \limits _{j=1}^{m}\bar{{\mathcal {S}}}_{i,j}\beta _j^{(r)} \right\} ^2 K_{\delta }(\left\Vert s_{\text {sim},i}-s_{\text {obs}}\right\Vert )+ \lambda \left\Vert \beta ^{(r)}\right\Vert _{2}^2; \end{aligned}$$

where $K_{\delta }(\cdot )$ is a Gaussian kernel with bandwidth or scale parameter $\delta$ given as

9 $$\begin{aligned} K_{\delta }(\left\Vert s_{\text {sim},i}-s_{\text {obs}}\right\Vert )= \omega _i=\frac{1}{ \sqrt{2 \pi \delta }} \textrm{e}^ {\frac{-1}{2\delta ^2} \left\Vert s_{\text {sim},i}-s_{\text {obs}}\right\Vert ^2}, \end{aligned}$$

and $\left\Vert s_{\text {sim},i}-s_{\text {obs}}\right\Vert =\rho (s_{\text {sim},i},s_{\text {obs}})$ is the weighted distance (computed using Eq. 2) between $s_{\text {sim},i}$ and $s_{\text {obs}}$; and the penalty term $\lambda \left\Vert \beta ^{(r)}\right\Vert _{2}^2= \lambda \sum \limits _{j=1}^{m}\beta _{j}^{(r)2}$ is the *L*2 regularisation element, with $\lambda$ representing the biasing or penalty parameter.

Similarly, the loss function for the weighted lasso regression ${\mathcal {L}}_{\text {lasso}}^{(r)}$ is defined such that

10 $$\begin{aligned} {\mathcal {L}}_{\text {lasso}}^{(r)}=\sum _{i=1}^{\eta } \left\{ \theta _i^{(r)} - \alpha ^{(r)} - \sum \limits _{j=1}^{m}\bar{{\mathcal {S}}}_{i,j}\beta _j^{(r)} \right\} ^2 K_{\delta }(\left\Vert s_{\text {sim},i}-s_{\text {obs}}\right\Vert )+ \lambda \sum \limits _{j=1}^{m} \big | \beta _{j}^{(r)} \big |. \nonumber \\ \end{aligned}$$

To minimise the loss functions (given by Eqs. 8 and 10) respectively, the variables in these equations are updated using the transformed variables defined in Eq. 11. The estimates of $\beta ^{(r)}$ and $\alpha ^{(r)}$ are then obtained separately (by initially ignoring the intercept $\alpha ^{(r)}$ in Eq. 7 prior to fitting the regression model) since the predictors and the dependent variables are respectively mean centred and re-scaled using $\sqrt{\omega _i}$ to obtain a set of variables with similar scaling (where the latter is motivated by Midi and Zahari (2008)); such that for $1 \le i \le \eta$ and $1 \le j \le m$:

11 $$\begin{aligned} \theta _i^{(r)*}= \sqrt{\omega _i} \left( \theta _i^{(r)}-{\bar{\theta }}^{(r)} \right) \qquad \text {and} \qquad \bar{{\mathcal {S}}}_{ij}^{*}= \sqrt{\omega _i}\left( \bar{{\mathcal {S}}}_{ij}- \bar{\bar{{\mathcal {S}}}}_j \right) , \end{aligned}$$

where ${\bar{\theta }}^{(r)}$ is the weighted mean of $\theta _i^{(r)}$, and $\bar{\bar{{\mathcal {S}}}}_j$ is the weighted mean of the *j*th predictor. The reason for the use of the re-scaled variables is that since these penalised regression methods regularise the linear regression by imposing a penalty based on the size or magnitude of the regression coefficients, they require the variables (predictors and posterior samples) to have similar measurement scales in order to assess their contributions to the penalised terms fairly, while maintaining the information content of the variables after re-scaling. Hence, Eqs. 8 and 10 are transformed (without the intercept) such that

12 $$\begin{aligned} {\mathcal {L}}_{\text {ridge}}^{(r)*}= \sum _{i=1}^{\eta } \left\{ \theta _i^{(r)*} - \sum \limits _{j=1}^{m} \bar{{\mathcal {S}}}_{i,j}^{*} \beta _j^{(r)*} \right\} ^2 \omega _i+ \lambda \sum \limits _{j=1}^{m}\beta _{j}^{(r)*2}, \end{aligned}$$

and

13 $$\begin{aligned} {\mathcal {L}}_{\text {lasso}}^{(r)*}= \sum _{i=1}^{\eta } \left\{ \theta _i^{(r)*} - \sum \limits _{j=1}^{m} \bar{{\mathcal {S}}}_{i,j}^{*} \beta _j^{(r)*} \right\} ^2 \omega _i+ \lambda \sum \limits _{j=1}^{m} \big | \beta _{j}^{(r)*} \big |, \end{aligned}$$

where $\beta _j^{(r)*}$ are the regression coefficient corresponding to the scaled predictors. For the WRR, the estimate of $\beta _j^{(r)*}$ can be obtained analytically (Arkin and Montgomery 1980) (by minimising Eq. 12) such that

14 $$\begin{aligned} {\hat{\beta }}_{m \times 1}^{(r)*}= (X_{m \times \eta }^{\top }W_{\eta \times \eta }X_{\eta \times m}+\lambda I_{m \times m})^{-1}X_{m \times \eta }^{\top }W_{\eta \times \eta } \theta _{\eta \times 1}^{(r)*} \quad 1 \le r \le n; \end{aligned}$$

where $I_{m \times m}$ is an $m \times m$ identity matrix, *W* is a diagonal weighting matrix with the *i*th diagonal element given by

$$\begin{aligned}{} & {} \omega _i=W_{ii}=K_{\delta }(\left\Vert s_{\text {sim},i}-s_{\text {obs}}\right\Vert ), \quad 1 \le i \le \eta ,\\{} & {} X= \begin{bmatrix} \bar{{\mathcal {S}}}_{1,1}^{*} &{} \bar{{\mathcal {S}}}_{1,2}^{*}&{} \cdots &{}\bar{{\mathcal {S}}}_{1,m}^{*}\\ \vdots &{}\vdots &{} \ddots &{} \vdots \\ \bar{{\mathcal {S}}}_{\eta ,1}^{*}&{}\bar{{\mathcal {S}}}_{\eta ,2}^{*}&{} \cdots &{}\bar{{\mathcal {S}}}_{\eta ,m}^{*} \end{bmatrix}, \quad \theta ^{(r)*}= \begin{bmatrix} \theta _1^{(r)*}\\ \vdots \\ \theta _{\eta }^{(r)*} \end{bmatrix}, \\{} & {} {\bar{\theta }}^{(r)}=\frac{ \sum \limits _{i=1}^{\eta } \omega _i \theta _i^{(r)}}{\sum \limits _{i=1}^{\eta } \omega _i}, \quad \text {and} \quad \bar{\bar{{\mathcal {S}}}}_j= \frac{\sum \limits _{i=1}^{\eta } \omega _i \bar{{\mathcal {S}}}_{ij}}{\sum \limits _{i=1}^{\eta } \omega _i}. \end{aligned}$$

However, for WLR, we obtain estimates of $\beta ^{(r)*}$ by numerically minimising Eq. 13 for all $\beta ^{(r)*} \in {\mathbb {R}}^m$ (with the help of the *glmnet* R package Hastie and Qian 2014) since the exact form can be determined analytically. To obtain an expression for the intercept $\alpha ^{(r)}$ in Eq. 7 per standard practice in regression (based on either WRR or WLR), it is not difficult to check that the exact estimate of the intercept term (after reverse variable transformation of Eq. 11 into their respective original scales after model fitting) is

15 $$\begin{aligned} {\hat{\alpha }}^{(r)}= {\bar{\theta }}^{(r)}- \sum _{j=1}^{m} {\hat{\beta }}_j^{(r)*} {\bar{X}}_j, \end{aligned}$$

where ${\bar{X}}_{j}= \frac{\sum \limits _{i=1}^{\eta } \omega _i X_{ij}}{\sum \limits _{i=1}^{\eta } \omega _i}$, ${\bar{\theta }}^{(r)}$ is the weighted mean of $\theta ^{(r)}$ and ${\hat{\beta }}_j^{(r)*}$ is the estimate of the regression coefficient corresponding to the *j*th transformed predictor. ${\hat{\alpha }}^{(r)}$ is a quantity denoting the adjusted posterior means on a logarithmic scale in the current study (since our unadjusted posterior samples were on a logarithmic scale). Hence, the required posterior mean adjustment of the *r*th model parameter is estimated by taking inverse of its logarithmic form (given by Eq. 15) such that

16 $$\begin{aligned} {\hat{\alpha }}_{\text {adjust}}^{( r)}= \textrm{e}^ {{\hat{\alpha }}^{(r)}}, \quad r=1,2,\cdots ,n. \end{aligned}$$

It is imperative to note that the exponential transform of the estimate of ${\hat{\alpha }}^{(r)}$ in Eq. 16 holds since the current study assumes the unadjusted posterior samples were obtained on a logarithmic scale. An exponential transformation is unnecessary for other studies where the unadjusted posterior samples were obtained based on their original scales. In addition, the adjusted posterior distribution $\theta _{\text {adjust}}^{(r)}$ (on logarithmic scale) for the *r*th model parameter is derived from Eq. 17 such that

17 $$\begin{aligned} \theta _{\text {adjust},i}^{(r)}=\theta _i^{(r)} - \sum _{j=1}^{m}{\hat{\beta }}_j^{(r)*}\bar{{\mathcal {S}}}_{ij}, \quad i=1,2,\cdots , \eta . \end{aligned}$$

The *glmnet* package in R (Hastie and Qian 2014) is used to obtain the optimal value of the penalty parameter $\lambda$ via cross-validation, achieving the least predictive error before posterior adjustments. Also, the optimal value of the bandwidth or smoothing parameter $\delta$ of the Gaussian kernel $K_{\delta }(\cdot )$ (given by Eq. 9) is adaptively estimated (based on the weighted distances between the simulated and observed summary statistics) via a cross-validation procedure (which minimises the asymptotic mean integrated squared error) using the *kedd* package in R (Guidoum 2020). In this study, 95% credible intervals of posterior mean estimates are estimated based on the Equal-Tailed Interval (ETI) of posterior distributions using the *bayestestR* package in R (Makowski et al. 2019).

## Results

### Introduction

The results of a numerical experiment based on our stochastic simulation model under predefined parameter settings and calibrated by the proposed ABC methods (comprising the weighted-iterative ABC and the proposed regression adjustments for posterior correction) are presented in Sect. 3.2. The goal is to evaluate the effectiveness of our modified ABC-SMC sampler and investigate the identifiability of the stochastic model for the gyrodactylid-fish system using pseudo-observed data. In Sect. 3.3, our stochastic simulation model is then fitted to the observed empirical data. Following additional posterior predictive checks (as outlined in Sect. 3.3), the best-adjusted posterior samples are utilised for Bayesian hypothesis testing to address the main research questions (denoted as 1–4) in Sect. 3.5.

### Results of the Numerical Experiment at Predefined Parameter Values

A detailed description of the numerical experiment and its results are summarised under Supplementary S4 in Additional supplementary material. We generated pseudo-observed data by simulating our stochastic model at predefined parameter values on a logarithmic scale. Subsequently, our weighted-iterative ABC was employed to fit the model to the pseudo-observed data. The quantiles of ABC distances, which quantify the discrepancy between pseudo-observed and simulated data, decreased monotonically across the ABC time steps. This suggested the improved performance and convergence of our modified ABC-SMC algorithm, resulting in an iteratively better approximation of the true posterior distribution. Following posterior correction using our proposed ridge-adjusted and lasso-adjusted regression methods (defined in Sect. 2.5), we observed that the unadjusted and ridge-adjusted posterior estimates resulted in relatively lower biases (with their model parameter estimates close to predefined true parameter values) and lower mean squared error (MSE) than the lasso-adjusted posterior estimates.

**Fig. 2:**
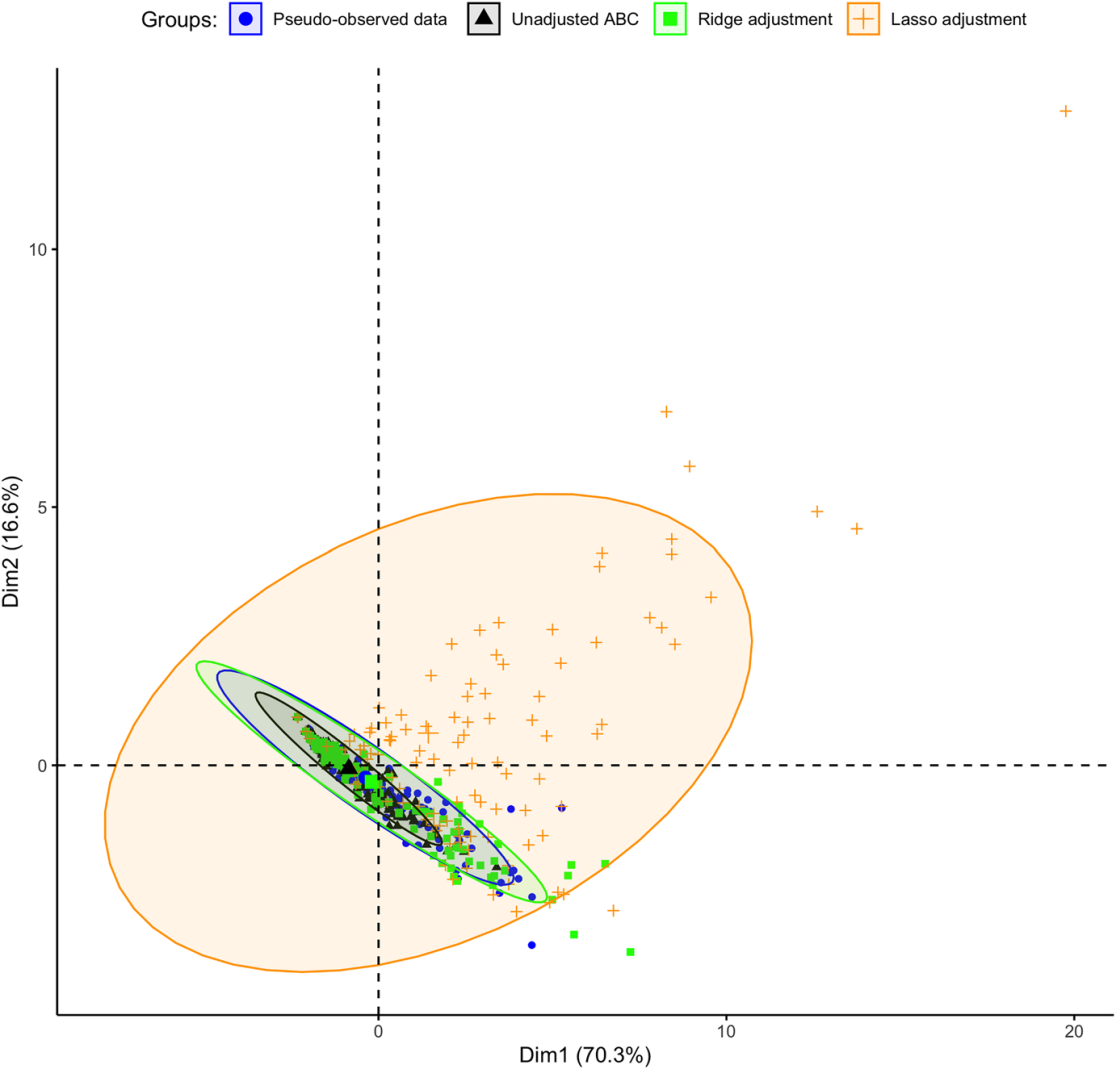
PCA plot describing the variability and hierarchical relationship between the pseudo-observed and simulated data (based on the unadjusted and regression-adjusted posterior estimates) within a lower dimensional space

**Fig. 3:**
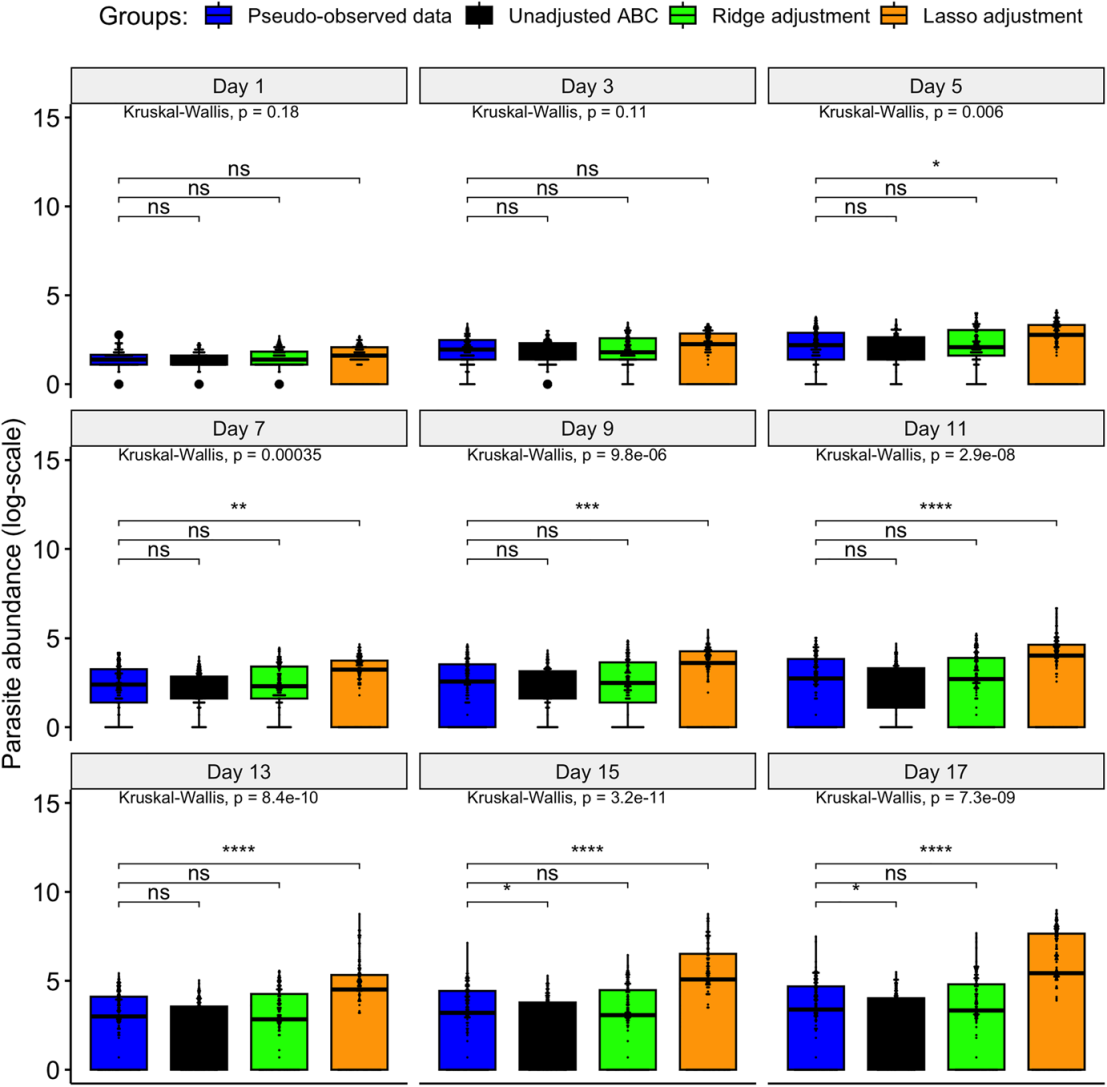
Comparative distribution plot of the pseudo-observed data and the different simulated datasets (where ^ns^p-value non-significant; ^∗^ p$<0.05$; ^∗∗^ p$<0.01$; ^∗∗∗^ p$<0.001$; ^∗∗∗∗^ p$<0.0001$)

Using the *vegan* R package (Oksanen et al. 2019), a principal coordinate analysis (PCoA) was performed to visualise similarities or dissimilarities among ABC posterior samples in a lower-dimensional space. We found a similarity between the unadjusted posterior and the ridge-adjusted posterior samples, in contrast to the lasso-adjusted posterior. However, a multivariate homogeneity test showed statistically insignificant variability among the three ABC posterior approximation methods. We employed principal component analysis (PCA) to examine further the distribution of simulations derived from the unadjusted and adjusted posteriors, and to identify potential patterns that may exist between the datasets within reduced dimensional space. Simulations derived from the unadjusted posterior exhibit spatial concentration within the pseudo-observed data. In contrast, the pseudo-observed data aligns more closely with simulations based on the ridge-adjusted posterior, and the latter set is contained within simulations derived from the lasso-adjusted posterior (see Fig. 2). We observed no statistical difference between the distribution of the pseudo-observed data and the simulated data derived from the ridge-adjusted posterior across the observation time points compared to that of the unadjusted and lasso-adjusted posteriors (see Fig. 3). Hence, the ridge-adjusted posterior correction method demonstrated a superior model fit compared to the lasso adjustment method, thereby improving upon the unadjusted posterior. This finding may or may not consistently align with the actual empirical data during the ABC fitting process.

### ABC Fitting of the Stochastic Model Given the Empirical Data

**Fig. 4:**
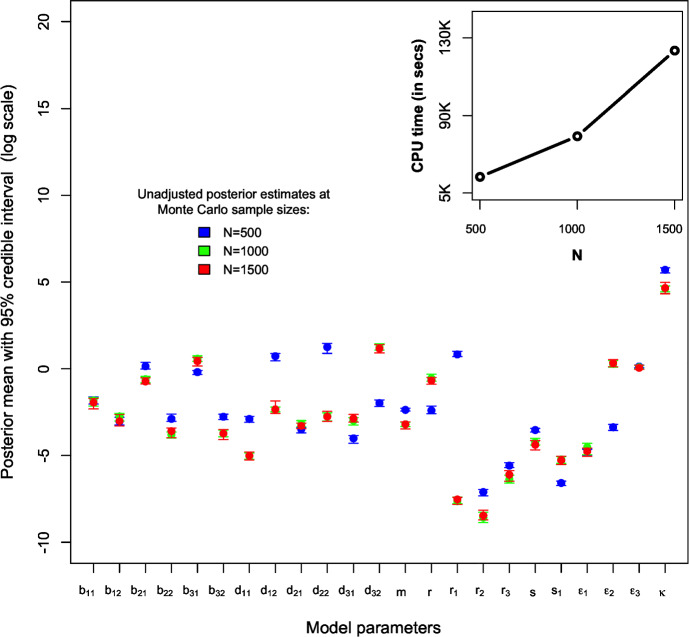
Comparative plot of the unadjusted posterior mean estimates with their respective 95% credible intervals (on logarithmic scale) at different values of $500 \le N \le 1500$ with a plot of their respective computational times (top-right)

The stochastic model, characterised by multiple parameters as detailed in Table 2, was also fitted using the proposed weighted-iterative ABC method, as outlined in Algorithm 1, at Monte Carlo sample sizes of $N=500$, $N=1000$, and $N=1500$, based on the empirical data (described in Sect. 2.1). The findings of Twumasi (Twumasi (2022), pp. 186–203) also influenced the choice of *N* in this study, where a simple numerical experiment was conducted based on a toy model with a multivariate normal likelihood and a known analytical posterior distribution. The experiment demonstrated that the resulting posterior is consistently compatible and independent of *N* for values ranging from at least 500 to 5000. However, the computational time for ABC increased quadratically with higher values of *N*. In this study, we considered a Monte Carlo sample size range of $500 \le N \le 1500$ during the ABC fitting of our stochastic simulation model, aiming to identify the minimum value of *N* at which the ABC posterior converges to similar estimates. Figure 4 shows that at values of $N=1000$ and 1500, the posterior estimates are consistent with quadratically increasing computational times. The ABC marginal density plots of the unadjusted posteriors at these Monte Carlo samples are presented as Supplementary Figures in Additional supplementary material. Thus, the Monte Carlo sample size of $N=1500$ is sufficient to fit our stochastic model based on the weighted-iterative ABC, as also revealed in the numerical experiment in Supplementary S4 in Additional supplementary material. The resulting posterior samples at $N=1500$ were considered for further ABC post-processing analysis with the two penalised regression adjustment methods.

**Fig. 5:**
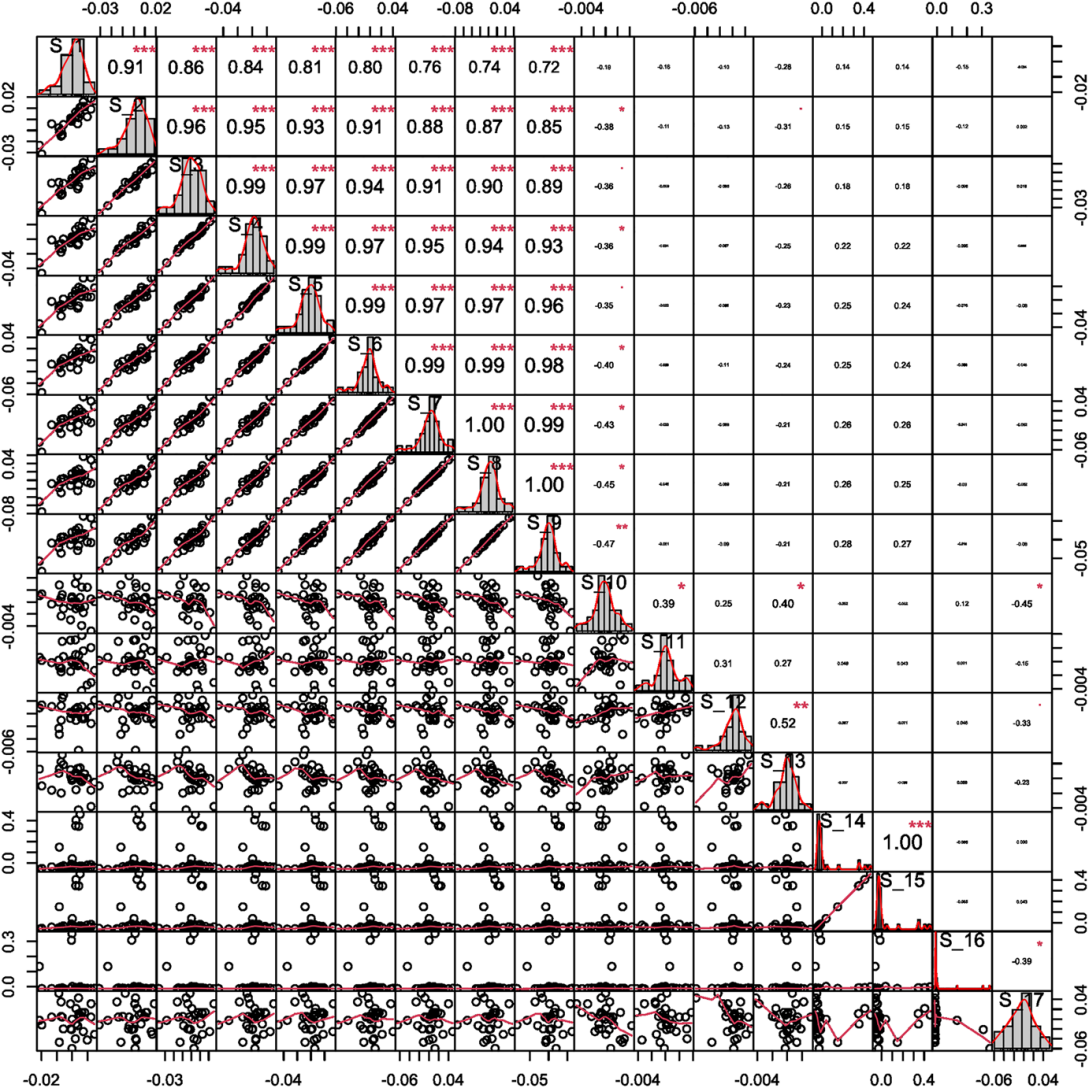
Correlation matrix plot indicating high multicollinearity between some of the 17 regression predictors (denoted by $S_i$, $1\le i \le 17$ in the neighbourhood of the observed summary statistics) in the modified regression-adjusted ABC (with *L*2 regularisation)

**Fig. 6:**
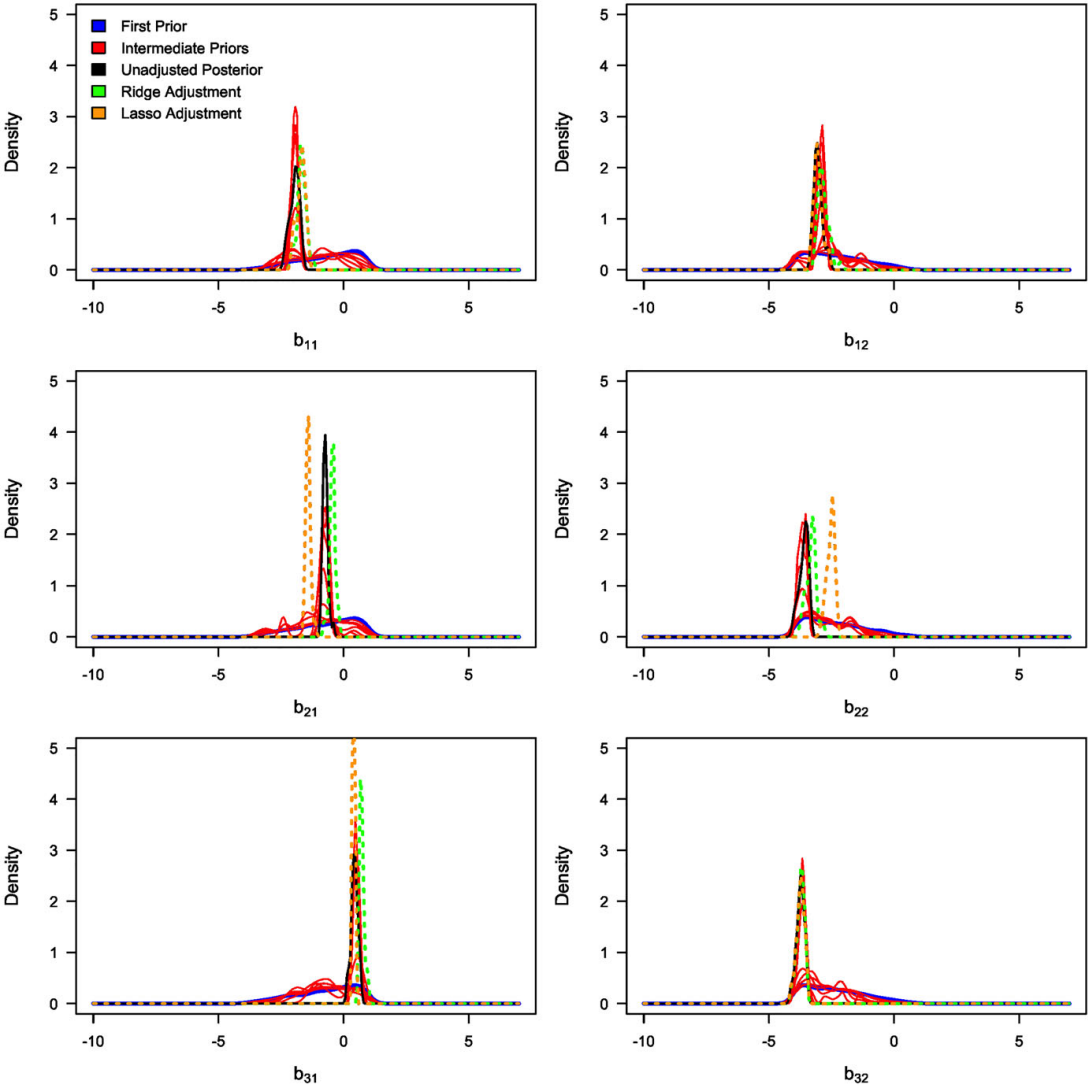
Marginal density plots of the unadjusted (in black) and adjusted (in green) posterior distributions of model parameters: $b_{11}$, $b_{12}$, $b_{21}$, $b_{22}$, $b_{31}$, and $b_{32}$ against the sequentially improving prior distributions (x-axis on a logarithmic scale) (Color figure online)

**Fig. 7:**
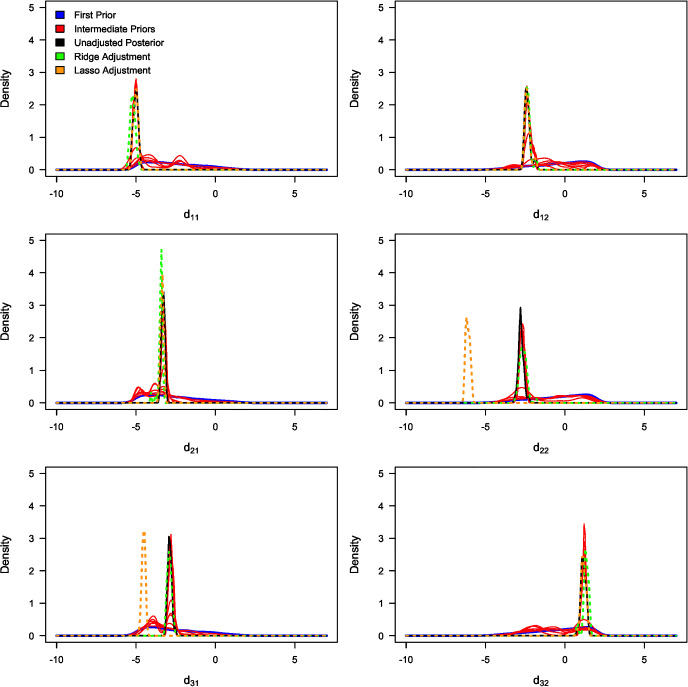
Marginal density plots of the unadjusted (in black) and adjusted (in green) posterior distributions of model parameters: $d_{11}$, $d_{12}$, $d_{21}$, $d_{22}$, $d_{31}$, and $d_{32}$ against the sequentially improving prior distributions (x-axis on a logarithmic scale) (Color figure online)

**Fig. 8:**
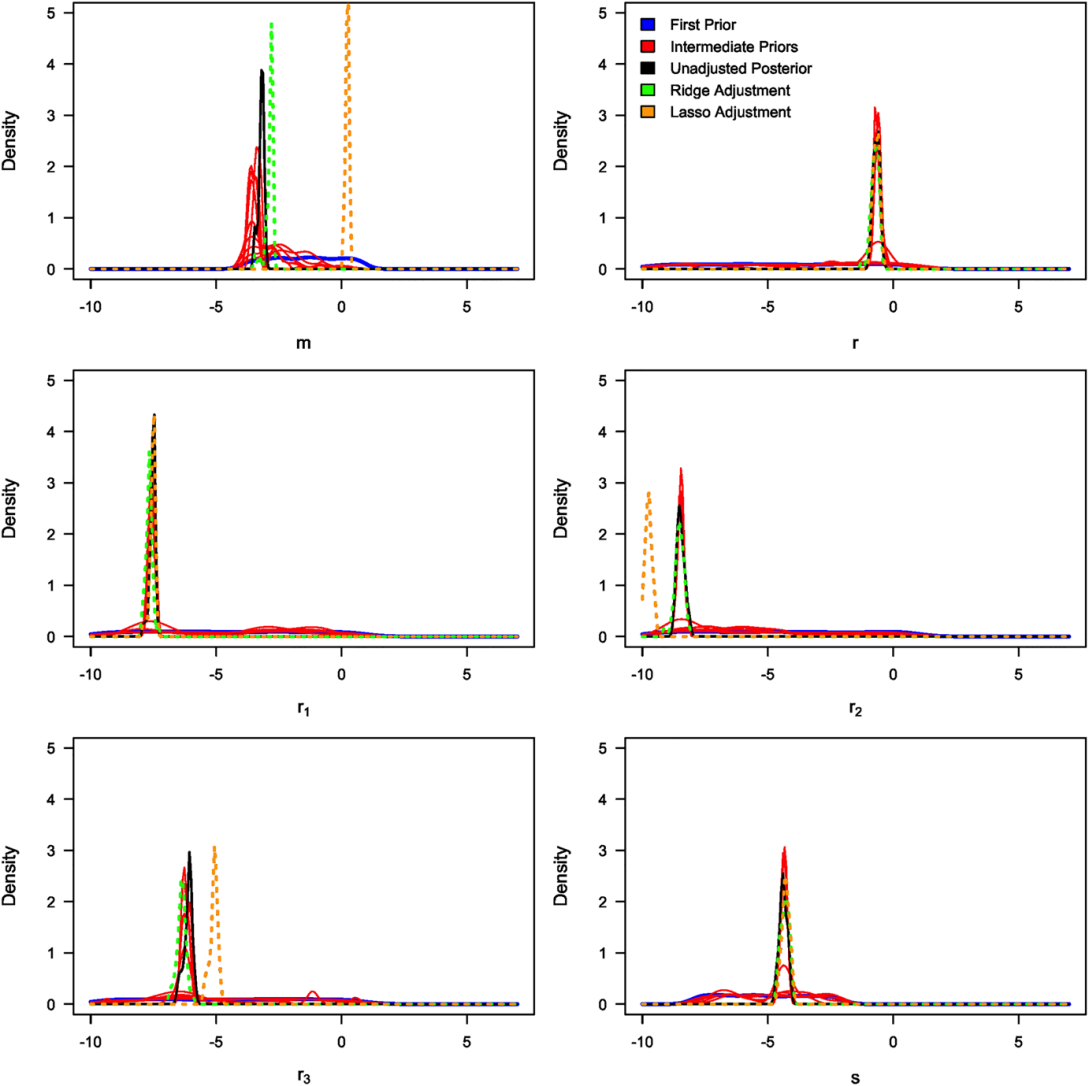
Marginal density plots of the unadjusted (in black) and adjusted (in green) posterior distributions of model parameters: *m*, *r*, $r_1$, $r_2$, $r_3$, and *s* against the sequentially improving prior distributions (x-axis on a logarithmic scale) (Color figure online)

**Fig. 9:**
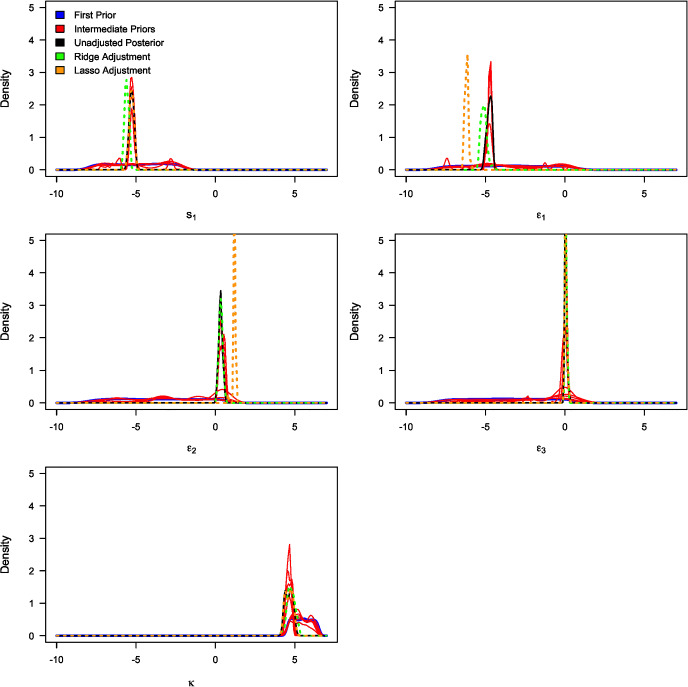
Marginal density plots of the unadjusted (in black) and adjusted (in green) posterior distributions of model parameters: $s_1$, $\epsilon _1$, $\epsilon _2$, $\epsilon _3$, and $\kappa$ against the sequentially improving prior distributions (x-axis on a logarithmic scale) (Color figure online)

**Fig. 10:**
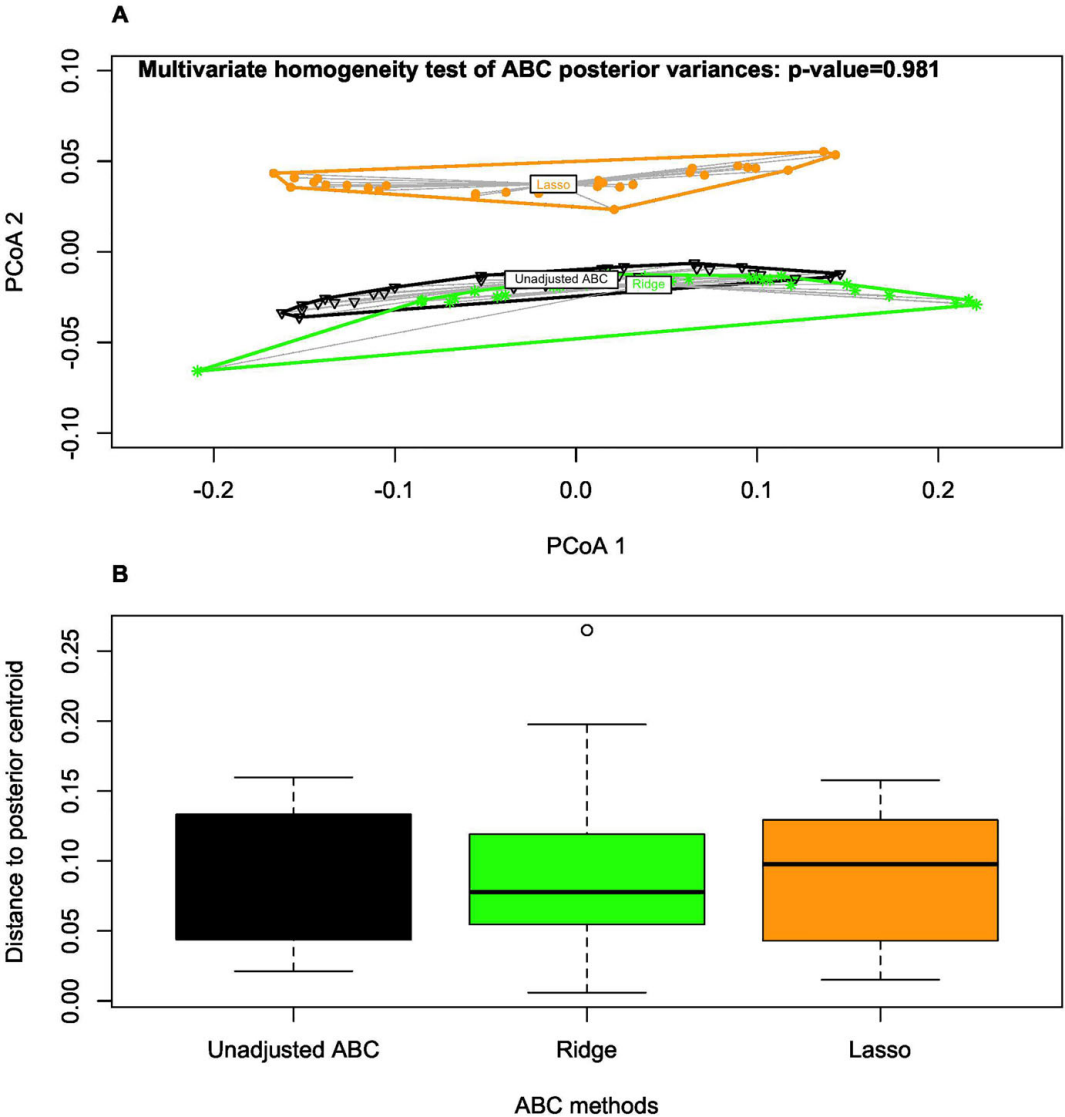
PCoA plot of the similarities between posterior samples under a lower-dimensional space (Part A) and the distribution of the average distances of the posterior samples to their posterior centriod (Part B) between the unadjusted ABC and the two penalised regression-adjusted ABC methods (based on ridge and lasso regularisations)

Table 3 summarises the unadjusted and adjusted posterior mean estimates of the underlying model parameters along with their respective 95% credible intervals. Due to high multicollinearity among certain regression predictors, as evidenced by Fig. 5 (for instance), the standard Beaumont et al. (2002) local-linear regression (with heteroscedastic errors) could not be implemented. This limitation arose due to the non-invertible matrices in its estimator in the presence of multicollinearity. The marginal density plots for the unadjusted and adjusted posterior distributions of the 23 parameters against sequentially improving priors are shown in Figs. 6, 7, 8 and 9. As observed in the numerical experiment (under Sect. 3.2), PCoA revealed that the unadjusted and ridge-adjusted posterior samples were similar, in contrast to the lasso-adjusted posterior. However, there was no statistically significant difference in the variability among these posterior samples, as depicted in Fig. 10.

**Table 3:** Unadjusted and adjusted posterior mean estimates of the 23 parameters of the multidimensional stochastic model with their respective 95% credible intervals (C.I.)

Parameters	Unadjusted mean (95% C.I.)	Ridge-adjusted mean (95% C.I.)	Lasso-adjusted mean (95% C.I.)
*Base simulation parameters*			
$b_{11}$	0.1426(0.0991 to $-$0.1807)	0.1796(0.1291 to $-$0.2574)	0.1851(0.1319 to $-$0.2325)
$b_{12}$	0.0489(0.0371, 0.0717)	0.0572(0.0444 to $-$0.0903)	0.0485(0.0371 to $-$0.0717)
$b_{21}$	0.4831(0.4158 to $-$0.5936)	0.6463(0.4901 to $-$0.7983)	0.2420(0.2102 to $-$0.2908)
$b_{22}$	0.0273(0.0187 to $-$0.0331)	0.0351(0.0251 to $-$0.0566)	0.0792(0.0585 to $-$0.0995)
$b_{31}$	1.5563(1.1785 to $-$1.9272)	2.0180(1.8465 to $-$2.6066)	1.4905(1.3930 to $-$1.6355)
$b_{32}$	0.0240(0.0169 to $-$0.0301)	0.0237(0.0173 to $-$0.0295)	0.0232(0.0169 to $-$0.0301)
$d_{11}$	0.0066(0.0052 to $-$0.0082)	0.0054(0.0042 to $-$0.0073)	0.0067(0.0052 to $-$0.0082)
$d_{12}$	0.0960(0.0762 to $-$0.1555)	0.0967(0.0767 to $-$0.1613)	0.0933(0.0761 to $-$0.1554)
$d_{21}$	0.0373(0.0317 to $-$0.0437)	0.0327(0.0213 to $-$0.0371)	0.0351(0.0302 to $-$0.0404)
$d_{22}$	0.0638(0.0485 to $-$0.0860)	0.0710(0.0522 to $-$0.1113)	0.0022(0.0019 to $-$0.0028)
$d_{31}$	0.0567(0.0464 to $-$0.0718)	0.0538(0.0449 to $-$0.0706)	0.0110(0.0089 to $-$0.0129)
$d_{32}$	3.2213(2.4965 to $-$4.0057)	3.8354(2.1862- to $-$4.6909)	3.2046(2.4984 to $-$4.0079)
*m*	0.0407(0.0312 to $-$0.0469)	0.0584(0.0356 to $-$0.0649)	1.2563(1.0858 to $-$1.3808)
*r*	0.5112(0.4115 to $-$0.6076)	0.4836(0.3509 to $-$0.5837)	0.5050(0.4117 to $-$0.6080)
*s*	0.0126(0.0093 to $-$0.0159)	0.0133 (0.0100 to $-$0.0166)	0.0136(0.0104 to $-$0.0176)
$\kappa$	104.5657(75.5036 to $-$145.1051)	118.0921(81.8550 to $-$175.3728)	99.4674(75.4581 to $-$145.0316)
*Additional simulation parameters*			
$\epsilon _1$	0.0086(0.0064 to $-$0.0103)	0.0059(0.0048 to $-$0.0089)	0.0020(0.0017 to $-$0.0024)
$\epsilon _2$	1.3875(1.1172 to $-$1.6996)	1.4134(1.1469 to $-$1.7348)	1.4134 (1.1469 to $-$1.7348)
$\epsilon _3$	1.0586(0.9676 to $-$1.2343)	1.0580(0.9918 to $-$1.1926)	1.0489(0.9678 to $-$1.2346)
$r_1$	0.000536(0.000404 to $-$0.000609)	0.000452(0.000360 to $-$0.000577)	0.000538(0.000404 to $-$0.000609)
$r_2$	0.000212(0.000166 to $-$0.000290)	0.000207(0.000136 to $-$0.000258)	0.000057(0.000047 to $-$0.000075)
$r_3$	0.002216(0.001535 to $-$0.002832)	0.001641(0.001134 to $-$0.002276)	0.006137(0.004307 to $-$0.007814)
$s_1$	0.0052(0.0040 to $-$0.0064)	0.0037(0.0031 to $-$0.0045)	0.0051(0.0040 to $-$0.0064)

### Additional Posterior Predictive Analysis Using PCA and Estimated Coverage Probabilities

**Fig. 11:**
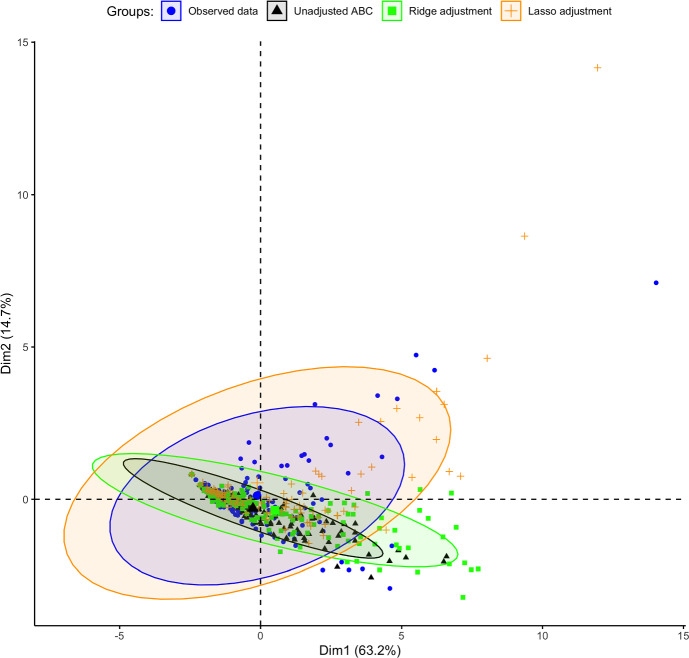
PCA plot describing the variability and relationship between the observed empirical and simulated data (based on the unadjusted and regression-adjusted posterior estimates) within a lower dimensional space

PCA was employed to assess the distributions of simulations derived from the unadjusted posterior and two regression-adjusted posteriors compared to the study’s empirical data. The aim was to explore patterns among them in a lower-dimensional space. As illustrated in Fig. 11, the observed data are spatially distributed within the simulated data derived from the lasso-adjusted posterior. In contrast, simulations from the unadjusted and ridge-adjusted posteriors overlap with the observed data. This implies that the observed data exhibit similar patterns or distributions to the simulated data derived from the lasso-adjusted posterior in the reduced-dimensional space, more so than the simulated data obtained from the unadjusted and ridge-adjusted posteriors.

**Fig. 12:**
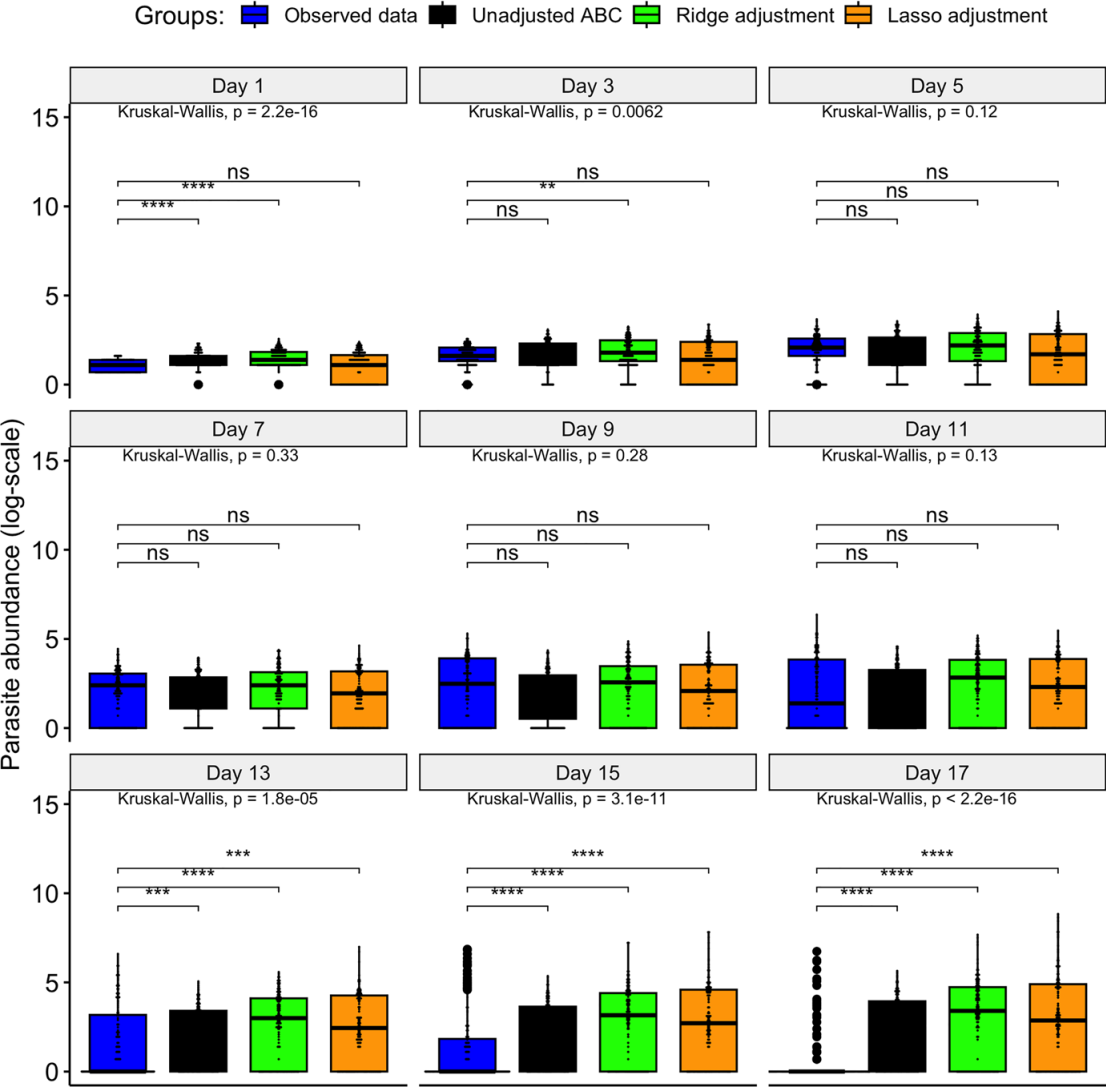
Comparative distribution plot of the empirical data and the simulated datasets (where ^ns^p-value non-significant; ^∗^ p$<0.05$; ^∗∗^ p$<0.01$; ^∗∗∗^ p$<0.001$; ^∗∗∗∗^ p$<0.0001$)

Figure 12 reveals no significant differences in the distribution of observed data and simulated data derived from the lasso-adjusted posterior at observation time points from days 1 to 11. However, discrepancies emerge on days 13 to 17, indicating significant differences in distributions between the observed data and the three distinct simulated datasets (based on the unadjusted and the adjusted posteriors). This finding contrasts with the results of the numerical experiment (summarised under Sect. 3.2), where the ridge regression adjustment was observed to produce simulated data statistically similar to the empirical data. Consequently, we can infer varying performance of the ridge and lasso regression adjustment methods in correcting the unadjusted posterior and minimising dissimilarity between the observed and simulated data. Their performance thus depends on the specific experimental data considered during the ABC fitting of the stochastic model.

**Fig. 13:**
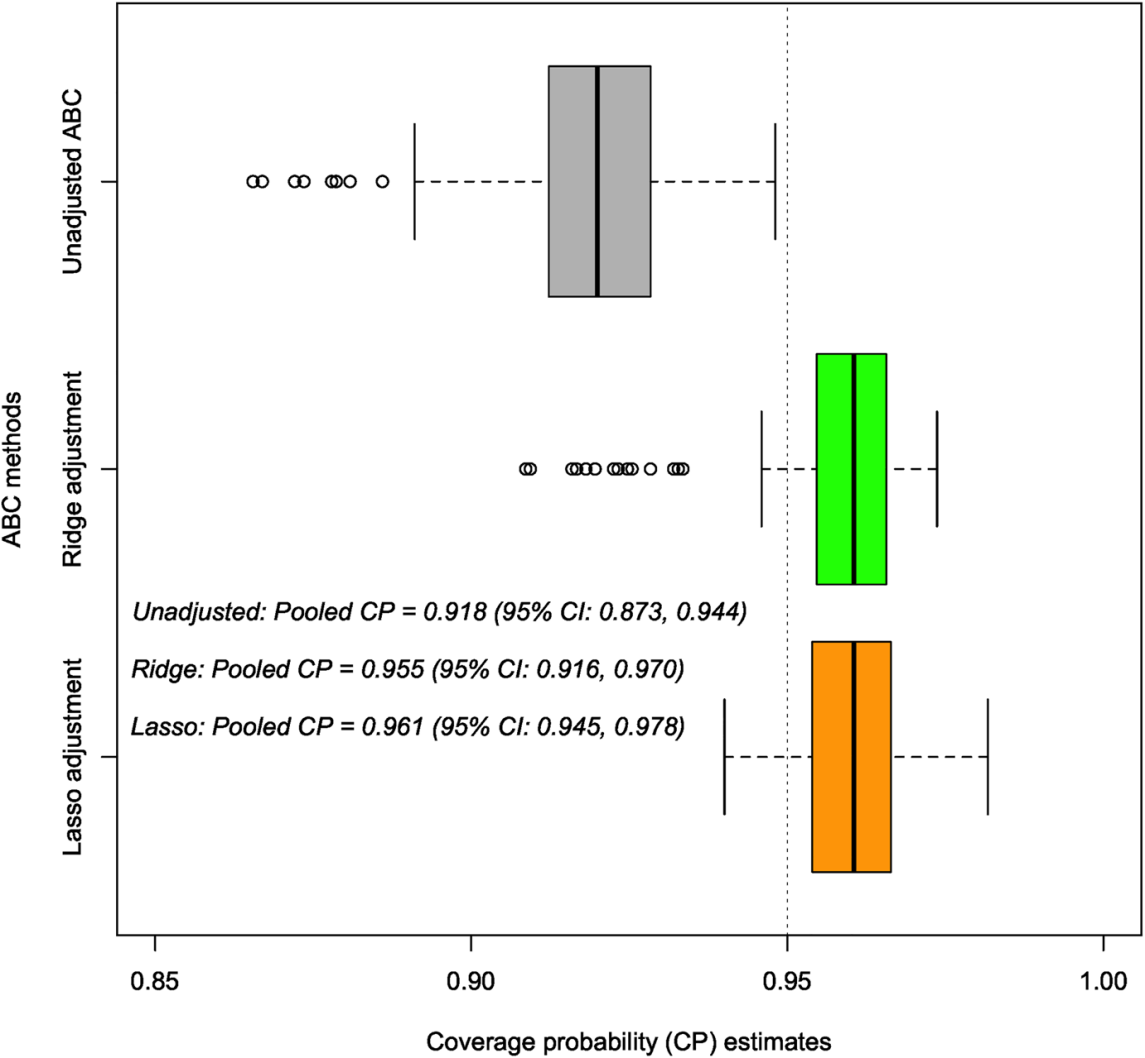
Distribution of estimated coverage probabilities based on simulated data from the unadjusted and regression-adjusted posteriors, with their corresponding estimated pooled CP and 95% credible intervals

We further computed coverage probabilities to assess the proportion of occurrences where the true empirical data fall within a 95% Bayesian prediction interval derived from simulations produced from the unadjusted and the two regression-adjusted posteriors, each replicated over 100 times (Fig. 13). As shown in Fig. 13, the estimated coverage probability (CP) closely approximates the nominal level of 95% for the 95% Bayesian prediction intervals generated from simulated data based on the two regression-adjusted posteriors. Additionally, the corresponding 95% credible intervals of the pooled CP (based on the regression-adjusted posteriors) contain the 95% nominal level, indicating well-calibrated prediction intervals that offer robust estimates of uncertainty about the empirical data. Notably, the lasso-adjusted posterior resulted in a relatively narrower credible interval width than the ridge-posterior regarding their pooled coverage probability estimates. Conversely, the simulated data derived from the unadjusted ABC posterior exhibited under-coverage, as evidenced by the corresponding 95% credible intervals of the pooled CP failing to encompass the nominal level of 95%. Given the observed empirical data, the lasso-adjusted regression method demonstrated a more substantial improvement in the resulting ABC posterior compared to the ridge-adjusted regression method (based on results from the PCA and estimated coverage probabilities). Therefore, the lasso-adjusted posterior is further considered for subsequent Bayesian hypothesis testing to evaluate various research hypotheses (under Sect. 3.5).

### Bayesian Hypothesis Testing Based on the Lasso-Adjusted Posterior Samples

#### Introduction

Classical null hypothesis significance testing (NHST) often employs a dichotomous decision rule to make conclusions regarding a parameter value of interest (i.e., the null value). This decision is based on either the p-value of a test statistic or an estimated confidence interval of the underlying parameter. In the latter method, which is favoured over the criticised p-value-dependent NHST decision (Lee 2016), we reject the null hypothesis if the parameter value falls outside a confidence interval. However, confidence intervals often fail to capture parameter uncertainty accurately and may suffer from coverage probability issues (Wilcox and Serang 2017). Some studies attempt to extend a similar logic to Bayesian posterior distributions, rejecting a parameter value if it falls outside a credible posterior interval (Kruschke and Liddell 2018). According to Kruschke and Liddell (2018), this standard decision rule raises two statistical issues. First, it can only reject and never accept a parameter value. Second, even if a null value is true, the decision process will eventually reject it with large posterior samples of the underlying parameter.

Other studies propose a more accurate decision rule, akin to frequentist equivalence testing (Rogers et al. 1993; Westlake 1981). This Bayesian approach involves integrating a region of practical equivalence (ROPE) around the null value and an estimated $100(1-\alpha )\%$ highest density interval (HDI) (Kruschke and Liddell 2018). Consequently, it is recommended that if an HDI is used to assess null values as part of a decision rule, the decision should also consider a ROPE around the null value (Kruschke 2014; McElreath 2020). In other words, a null value should not be rejected solely because it falls outside an HDI, as observed in previous studies (Kruschke 2011). The suggestion is to reject the null only when the HDI strictly falls outside the ROPE, indicating that the parameter’s most credible values are not practically equivalent to the null value. Acceptance of the null is warranted if the HDI lies entirely within the ROPE, and indecision prevails if there is an overlap (Kruschke and Liddell 2018; Schwaferts and Augustin 2020). For an extensive range of Bayesian decisions using ROPE, including more technical reports, refer to the work by Schwaferts and Augustin (2020).

In the current study, we simultaneously used the ROPE and HDI (which is dubbed in the literature as ROPE+HDI) to test relevant hypotheses concerning differences between some underlying parameters of our stochastic simulation model with the help of the adjusted posterior samples and the *bayestestR* package in R (Makowski et al. 2019). McElreath (2020) and Kruschke (2014) have recommended an 89% HDI to be an ideal choice compared to the usual 95% HDI for Bayesian hypothesis testing with ROPE. According to Kruschke (2014) the 95% HDI might not be the most appropriate for Bayesian posterior distributions due to potentially lacking stability if not enough posterior samples are drawn (as observed in the current study). Hence, an appropriate ROPE and an 89% HDI are considered for testing sets of hypotheses. Results from the Bayesian hypothesis test will aid in providing answers to research questions 1–4. Now, let us suppose a null hypothesis $H_0: \theta _1= \theta _2$ (or $d=\theta _1-\theta _2=0$), where $\theta _g \in R$ denotes model parameters corresponding to some independent groups $g=1,2$ (possibly identically distributed). The alternative hypothesis is defined as $H_1: \theta _1 \ne \theta _2$ (or $d=\theta _1-\theta _2 \ne 0$). Let ${\mathcal {A}}_{\text {I}}= \{[a,b] \mid a,b \in \Theta , a<b \}$ represent the action space w.r.t the HDI of the posterior distribution of $d=\theta _1-\theta _2$, and let ${\mathcal {A}}_R= [-0.5 \sigma _d,0.5 \sigma _d]$ denote the ROPE range (recommended by Norman et al. (2003)), where $\sigma _d$ is the standard deviation of the posterior samples of *d*. Let also suppose $\gamma =P({\mathcal {A}}_{\text {I}} \subseteq {\mathcal {A}}_R \mid d)$ denote the ROPE coverage probability (or the probability that elements of ${\mathcal {A}}_{\text {I}}$ fall within ${\mathcal {A}}_R$ given the posterior samples of *d*). Following Kruschke and Liddell (2018), we also reject or accept $H_0$ according to the following HDI+ROPE decision rule:

$$\begin{aligned} \text {ROPE equivalence decision}= \left\{ \begin{array}{ll} \text {reject} \quad H_0, &{} \gamma =0 \\ \text {indecisive}, &{} 0< \gamma <1 \\ \text {accept} \quad H_0,&{} \gamma =1.\\ \end{array}\right. \end{aligned}$$

The null hypothesis and the ROPE+HDI test described above can be modified to compare differences between model parameters corresponding to more than two groups similarly (as performed in the subsequent sections).

#### Assessing Differences Between the Birth Rate Model Parameters

We first tested three major hypotheses in relation to the birth rate parameters of the fitted stochastic model based on ROPE+HDI Bayesian tests (Table 4). The null hypotheses tested are as follows:

$$H_{01}$$: $$b_{i1}-b_{j1}=0$$, for $$i \ne j$$ and $$1 \le i,j, \le 3.$$

$$H_{02}$$: $$b_{i2}-b_{j2}=0$$, for $$i \ne j$$ and $$1 \le i,j, \le 3.$$

$$H_{03}$$: $$b_{i1}-b_{j2}=0$$, for $$i=j$$ and $$1 \le i,j, \le 3.$$

For the first null hypothesis ($H_{01}$), there is sufficient evidence to conclude that the birth rate of young *Gb* parasites (yet to reproduce) is significantly greater than the birth rates of young *G. turnbulli* strains (i.e., *Gt3* and *Gt* young parasites). Conversely, for the second null hypothesis ($H_{02}$), the birth rates of old *G. turnbulli* strains are found to be significantly greater than those of old *G. bullatarudis* parasites. However, based on the Bayesian test results, we arrive at an indecisive conclusion between *Gt3* and *Gt* parasites regarding the birth rates of both young and old parasites, respectively. Concerning the third hypothesis ($H_{03}$), our findings indicate that the birth rates of old parasites are significantly lower than those of their young counterparts across all three parasite strains. These findings show that the population growth of gyrodactylids is predominantly driven by young parasites, owing to their high birth rate. The heightened likelihood of reproduction in young *Gb* parasites compared to the two *G. turnbulli* strains may explain the observed high parasite abundance or mean intensities over time after analysing the empirical data by Twumasi et al. (2022). Past experimental investigations have demonstrated that gyrodactylids can undergo reproduction up to four times. However, given that the initial birth occurs before the parasite reaches two days of age, the population can persist with just two births (Denholm et al. 2013). Additionally, Denholm et al. (2013) revealed that the parasite’s first birth is the primary determinant of the overall population growth. This finding may explain why the present study observed a significantly greater birth rate in young gyrodactylids (who are yet to reproduce) than their older counterparts (with a birth history) across all strains.

**Table 4:** Results from the test of statistical differences between the birth rate parameters

Parameter	89% HDI	ROPE range	ROPE coverage (%)	Decision
*First hypotheses*				
$b_{11}-b_{21}$	$-$0.1183 to $-$0.0105	$-$0.0180 to 0.0180	11.54	Indecisive
$b_{11}-b_{31}$	$-$1.4555 to $-$1.1993	$-$0.0458 to 0.0458	0	Rejected
$b_{21}-b_{31}$	$-$1.3931 to $-$1.1377	$-$0.0456 to 0.0456	0	Rejected
*Second hypotheses*				
$b_{12}-b_{22}$	$-$0.0514 to $-$0.0023	$-$0.0087 to 0.0087	3.85	Indecisive
$b_{12}-b_{32}$	0.01371 to 0.0443	$-$0.0052 to 0.0052	0	Rejected
$b_{22}-b_{32}$	0.0368 to 0.0765	$-$0.0065 to 0.0065	0	Rejected
*Third hypotheses*				
$b_{12}-b_{12}$	0.0901 to 0.18381	$-$0.0146 to 0.0146	0	Rejected
$b_{21}-b_{22}$	0.1341 to 0.2005	$-$0.0131 to 0.0131	0	Rejected
$b_{31}-b_{32}$	1.3730 to 1.6009	$-$0.0389 to 0.0389	0	Rejected

#### Assessing Differences Between the Death Rate Model Parameters

Also, we test three major hypotheses concerning the death rate model parameters (Table 5). The null hypotheses tested are as follows:

$$H_{04}$$: $$d_{i1}-d_{j1}=0$$, for $$i \ne j$$ and $$1 \le i,j, \le 3.$$

$$H_{05}$$: $$d_{i2}-d_{j2}=0$$, for $$i \ne j$$ and $$1 \le i,j, \le 3.$$

$$H_{06}$$: $$d_{i1}-d_{j2}=0$$, for $$i=j$$ and $$1 \le i,j, \le 3.$$

In the absence of host immune response, a notable discrepancy exists in the mortality rates among the three parasite strains, with the death rate of the wild *G. turnbulli* being significantly higher than that of the other two parasite strains (with that of *Gb* > *Gt3*). However, when a host response is present (potentially attributed to rapid infrapopulation growth, high parasite virulence, or intense competition for resources), the wild *Gb* parasite attains the highest mortality rate, surpassing that of *Gt3* (> *Gt*). For *Gt3* and *Gb* parasite strains, the death rate in the absence of a host response is significantly lower than the mortality rate in the presence of an immune response. Conversely, a contrasting observation is noted for the *Gt* parasite strain, with the immune-induced death rate being significantly lower in comparison. The observed variation in the gyrodactylid death rate, contingent on the adaptive immunocompetency of the host, likely signifies a trade-off between effective parasite exploitation and the localised immune response of the host. This implies that the higher mortality rates observed in the *Gt3* and *Gb* parasite strains compared to the *Gt* strain, as revealed in the multi-state Markov modelling based on empirical data from the previous study by Twumasi et al. (2022), may be attributed to the host immune response, particularly as the infection intensifies, especially during the peak time of infection. While the temperature range effectively regulates the population dynamics of gyrodactylids, studies on *Gyrodactylus* have demonstrated that adaptive host immunity, which develops in most fish populations, can also contribute to the extinction of gyrodactylid populations on a fish host (Rubio-Godoy et al. 2012).

**Table 5:** Results from the test of statistical differences between death rate parameters

Parameter	89% HDI	ROPE range	ROPE coverage (%)	Decision
*Fourth hypotheses*				
$d_{11}-d_{21}$	$-$0.0327 to $-$0.0241	$-$0.0014 to 0.0014	0	Rejected
$d_{11}-d_{31}$	$-$0.006 to $-$0.0021	$-$0.0006 to 0.0006	0	Rejected
$d_{21}-d_{31}$	0.0198 to 0.0288	$-$0.0015 to 0.0015	0	Rejected
*Fifth hypotheses*				
$d_{12}-d_{22}$	0.0748 to 0.1371	$-$0.0099 to 0.0099	0	Rejected
$d_{12}-d_{32}$	$-$3.8805 to $-$2.5099	$-$0.2377 to 0.2377	0	Rejected
$d_{22}-d_{32}$	$-$3.9666 to $-$2.6215	$-$0.2352 to 0.2352	0	Rejected
*Sixth hypotheses*				
$d_{11}-d_{12}$	$-$0.1327 to $-$0.0704	$-$0.0099 to 0.0099	0	Rejected
$d_{21}-d_{22}$	0.0284 to 0.0382	$-$0.0016 to 0.0016	0	Rejected
$d_{31}-d_{32}$	$-$3.9574 to $-$2.615	$-$0.2353 to 0.2353	0	Rejected

#### Assessing Differences Between the Movement Rate Adjustment Parameters

We further test differences between movement rate adjustment parameters across the three parasite strains (Table 6). The strain-specific movement rate adjustment parameters are expected to account for the unique caudal-rostral preferences of the gyrodactylid strains in the simulation model (as confirmed in Twumasi et al. (2022)). Here, the null hypotheses are defined as follows:

$$H_{07}$$: $$\epsilon _{i}-\epsilon _{j}=0$$, for $$i \ne j$$ and $$1 \le i,j, \le 3.$$

Table 6 illustrates that the movement rate adjustment of the laboratory-bred *G. turnbulli* strain (*Gt3*) is significantly lower compared to both the wild *G. turnbulli* (*Gt*) and the wild *G. bullatarudis* (*Gb*) strains, whereas the movement rate of the *Gt* strain is relatively higher than that of the *Gb* strain. This observation suggests that the stochastic model is able to differentiate between the distinct microhabitat preferences of *Gt3* and *Gb* strains, as previously justified by Twumasi et al. (2022), particularly after the initial infection at the caudal region of the host. As supported by the previous work Twumasi et al. (2022), the *Gb* worms are initially placed at the caudal region of their fish host, which is not their most preferred microhabitat compared to the host’s rostral region. Consequently, the movement rate of the *Gb* parasites is expected to be relatively higher than that of *Gt3* to facilitate its rapid transition towards the rostral or head regions of their fish host over time, as discovered in the spatial-temporal analysis of the parasites’ microhabitat preference. Twumasi et al. (2022) also noted that the wild *G. turnbulli* strain changes its microhabitat preference over time, transitioning from the tail to the rostral region of the host based on empirical data. This finding confirms why the movement rate of the *Gt* strain is significantly higher than that of the *Gt3* strain, which rather prefers the caudal region of its fish host over time following the initial infection at the host’s caudal region.

**Table 6:** Results from the test of statistical differences between the movement rate adjustment parameters

Parameter	89% HDI	ROPE range	ROPE coverage (%)	Decision
*Seventh hypotheses*				
$\epsilon _{1}-\epsilon _{2}$	$-$3.6261 to $-$3.0855	$-$0.1327 to 0.1327	0	Rejected
$\epsilon _{1}-\epsilon _{3}$	$-$1.1860 to $-$0.9772	$-$0.0384 to 0.0384	0	Rejected
$\epsilon _{2}-\epsilon _{3}$	1.9888 to 2.5386	$-$0.1581 to 0.1581	0	Rejected

#### Assessing Differences Between the Immune Response Rate Adjustment Parameters as Well as the Sex-Specific Host Mortality Parameter

Finally, we test two different hypotheses in relation to the immune response rate adjustment parameters and the sex-specific host mortality parameter, respectively. The null hypotheses of these tests are defined as:

$$H_{08}$$: $$r_{i}-r_{j}=0$$, for $$i \ne j$$ and $$1 \le i,j, \le 3.$$

$$H_{09}$$: $$s_1=0.$$

Table 7 summarises the findings regarding $H_{08}$ and $H_{09}$. The analysis reveals significant differences in the immune response rate adjustment parameters and the significance of the model parameter $s_1$ from zero (representing the host mortality rate adjustment for male fish relative to female fish). These outcomes contribute valuable insights into whether the adaptive host immune response exhibits sex and host dependency and whether the mortality rate of male fish surpasses that of female fish, as indicated by the fitted stochastic model and evidence derived from empirical data during the multi-state Markov modelling in our earlier study (Twumasi et al. 2022). The Bayesian test results suggest a higher likelihood of mortality in male fish than female fish (with the latter as the reference category in the simulation model). Furthermore, it is inferred that the immune response rate of LA fish is significantly greater than that of OS stock, where the immune response rate of OS fish exceeds that of UA fish. The relatively high response in the LA and OS fish stocks may explain why their risks of death were lower than those of the UA fish (Twumasi et al. 2022). The current study found the LA fish’s immune response rate to be lower than that of male stocks in general. This observation aligns with the findings from the multi-state Markov model (Twumasi et al. 2022), which predicted a shorter duration of infection for male fish compared to infected female fish across all parasite strains, fish stocks, and host sizes, possibly attributed to the greater immune response in male fish relative to female fish.

**Table 7:** Results from the test of statistical differences between the immune response rate adjustment parameters and sex-specific host mortality parameter

Parameter	89% HDI	ROPE range	ROPE coverage (%)	Decision
*Eighth hypotheses*				
$r_{1}-r_{2}$	0.00038 to 0.00054	$-$0.00003 to 0.00003	0	Rejected
$r_{1}-r_{3}$	$-$0.0069 to $-$0.0040	0.00046 to 0.00046	0	Rejected
$r_{2}-r_{3}$	$-$0.0074 to $-$0.0045	$-$0.00046 to 0.00046	0	Rejected
*Ninth hypothesis*				
$s_1$	0.0041 to 0.0063	$-$0.00035 to 0.00035	0	Rejected

## Discussion and Conclusions

### Biological Implications of the Study

This study contributes mathematically and biologically to the gyrodactylid-fish system, offering insights that may apply to modelling other biological systems. Expanding our recent study into spatial-temporal parasite dynamics of this system (Twumasi et al. 2022), we have added to our understanding of this system by developing a novel individual-based stochastic simulation model to address, for the first time, other open biological questions through model-based Bayesian analysis. The birth rates for both young and old gyrodactylid parasites, as well as the death rates with and without immune response, were observed to differ significantly among the three parasite strains. We verified that the adaptive immune response to the progression of infection is dependent on host sex and host stock, with male fish being more susceptible to mortality from gyrodactylid infection. Additionally, the total number of *Gyrodactylus* parasites capable of occupying a host’s major body region ranged between 75 and 145, with an average value of over 100 parasites.

Our individual-based stochastic model, designed to enhance gyrodactylid simulations compared to an existing computer-based IBM, relies on model assumptions incorporating biological realism specific to the gyrodactylid-fish system. Empirical data and the biology of the system inform these assumptions. The current IBM for this system serves as a valuable tool for predicting gyrodactylid infection development on single hosts and forecasting optimal life history strategies of parasites (Oosterhout et al. 2008). However, in a realistic context, the time to host immune response may vary after infection, and localised immune response could be influenced by host and parasite genotype, surface area of body locations, and host sex.

In the examination of infrapopulation dynamics of gyrodactylids on their fish hosts, the existing IBM did not distinguish between different body regions of the host (tail fin, lower body, upper body, anal fin, dorsal fin, pelvic fins, pectoral fins, and head), including their respective surface areas. Additionally, it did not differentiate between young and old parasites and imposed restrictions on the maximum linear distance that parasites can move over time. In practice, there are unique microhabitat preferences specific to different gyrodactylid strains across diverse host populations over time (Twumasi et al. 2022). This spatial information needed incorporation for simulating the species-specific infrapopulation dynamics. Furthermore, the specific structure of the existing IBM software, along with its pseudo-codes, has yet to be explicitly and mathematically defined in the previous study (Oosterhout et al. 2008). This lack of clarity presents challenges in terms of implementation, result replication, and validation of their proposed model. It is noteworthy that the existing spatially explicit IBM software for this biological system is inaccessible, making it difficult to compare with the current study’s simulation model (see Oosterhout et al. (2008)). This limitation also underscored the necessity for a more robust simulation model for the gyrodactylid-fish system, as developed in the current study.

### Mathematical Implications of the Study

Sequential Monte Carlo samplers (ABC-SMC) are effective when coupled with sequential importance sampling (SIS) to generate particles in high posterior regions and mitigate issues of particle degeneration that often occur in other ABC samplers (Beaumont et al. 2009). In other studies, ABC summary statistics weighting, where very informative summaries are assigned higher weights, has also improved ABC posterior convergence compared to unweighted ABC analysis (Jung and Marjoram 2011). Thus, the present study capitalised on the relative importance of weighting summary statistics and ABC-SMC with SIS to improve ABC calibration in multi-parameter settings. This improvement is notable even with small Monte Carlo sample sizes during ABC implementation, particularly when dealing with high-dimensional summaries that capture data information, whether dependent or independent.

However, ABC-SMC samplers can also suffer from dimensionality issues, especially if many parameters must be estimated (Khazeiynasab and Qi 2021). To address this, Blum et al. (2013) suggested that the resulting ABC posterior can be adjusted based on either ridge or lasso regularisation procedures via regression adjustment, especially in the case of complex model fitting to minimise the dissimilarity between simulated and observed data. By extending Beaumont et al.’s Beaumont et al. (2002) local-linear regression adjustment method (to include L1 and L2 penalties, respectively), we have demonstrated in this study that our proposed penalised regression methods can improve the resulting ABC posterior distribution of ABC-SMC sampler based on findings from a numerical experiment as well as ABC fitting of our stochastic simulation model to an empirical data. Nonetheless, we found a data-dependent varying performance between our ridge and lasso regression adjustment methods in correcting the unadjusted posterior and minimising dissimilarity between the observed and simulated data. Thus, their relative performance will vary depending on the specific experimental data considered during the ABC fitting of a model.

### Limitation of the Study

The current study had a few limitations. First, our individual-based stochastic simulation model, designed for the standard 17-day experimental period, is specifically developed to explore the infrapopulation infection dynamics of gyrodactylids on a single fish. This limitation arises from the study design, and consequently, the model would need to be modified to examine infection transmission between hosts in a scenario where fish can interact. Also, the current study only investigated the infection dynamics of the gyrodactylid parasites within the standard 17-day experimental period. Hence, this study did not consider the interpopulation (or mixed-gyrodactylid) within-host infection dynamics, between-host transmission or intrapopulation infection dynamics (using a social network model), or long-term predictions beyond the standard 17-day infection period across the different host populations by adapting our stochastic simulation model.

###  Future Research Directions

The current study can be extended in several ways. Specifically, the following are future works concerning the stochastic simulation model, the modified sequential Monte Carlo ABC with adaptive importance sampling and the gyrodactylid-fish system:

- Within the stochastic simulation model, we assumed that the rate of localised host immune response (which occurs temporally as a function of the number of parasites at any of the host body locations) also depends on fish sex (with two levels) and type of fish (with three levels). Thus, the current study only considered the additive impact of the covariates (fish sex and fish stock) on the immune response rate without considering interaction effects. Future studies should consider the multiplicative or interaction effects of these covariates on the rate of localised immune response and compare the modified model with the current version with additive immune response rates.
- In the modified ABC-SMC sampler, we predetermined and fixed the decreasing tolerance thresholds and the final ABC stopping time, employing ten ABC time steps and associated tolerances. Subsequent research could propose adaptive tolerance strategies and a stopping rule, allowing the ABC algorithm to terminate upon achieving posterior convergence. Additionally, exploring the impact of various optimal perturbation kernels and employing other regularisation methods, such as elastic net regularisation (combining L1 and L2 penalties), could be valuable.
- Future investigations might focus on identifying low-dimensional and informative summary statistics for ABC fitting. This effort aims to refine ABC posterior approximations further, mitigating instances of model under- or over-fitting across different parasite-fish groups (especially towards the end of the infection period).
- Furthermore, the simulation time axis of the stochastic simulation model, or the observed time points, can be extended to enable predictions beyond the standard 17-day experimental period. This extension would facilitate assessing how infections are sustained over the long term across various host populations. Thus, an extended model should be developed and fitted using the proposed ABC methodologies with the help of experimental data.
- Future studies can further conduct *in silico* experiments that are challenging to explore experimentally because of similarities between gyrodactylids and other unfavourable experimental conditions that may prevail. This exploration involves modifying our stochastic simulation model to investigate mixed gyrodactylid parasite populations. Specifically, co-infections on a single or fish population can be examined based on existing knowledge about these gyrodactylid species, such as *G. turnbulli* and *G. bullatarudis* parasites. In addition, relevant ecological questions can be investigated regarding how the different *Gyrodactylus* species interact or compete and which one temporally wins at the individual host and population levels.
- Finally, future studies can develop a social network model coupled with our stochastic simulation model to describe the infection dynamics of a fish population and their interactions. The social network model should capture the parasite load for each fish over time but must not necessarily give the exact spatial locations of parasites on an individual host. The model should be calibrated using the appropriate ABC for network models.

**Supplementary S1:** Pseudo-codes of exact simulation and $\tau$-leaping for the CTMC simulation model. Here, we present the pseudo-codes for both the exact Stochastic Simulation Algorithm (SSA) and the hybrid $\tau$-leaping algorithm, designed for our multidimensional CTMC stochastic simulation model.

**Supplementary S2:** Determining an error bound for the Hybrid *tau*-leaping simulation model. Here, a reasonable choice of the error bound $\epsilon$ ($0 < \epsilon \ll$1) for the hybrid *tau*-leaping simulation model was investigated by exploring the trade-off between simulation accuracy and computational speed at some predefined parameter values based on 100 different simulation realisations or repetitions; where each simulation realisation corresponded to the nine observed parasite-fish groups (given fish sex, fish size, fish stock and parasite strain).

**Supplementary S3:** Projection of parasite numbers after fish mortality. Here, an optimised linear regression function is developed to aid in computing the summary statistics during ABC fitting of our sophisticated simulation model after premature host mortality (which also includes a proposed theorem and its mathematical proof).

**Supplementary S4:** Assessing the weighted-iterative ABC and regression adjustments using a numerical experiment. Here, we present the results of a numerical experiment conducted with our stochastic simulation model at pre-defined parameter values. The aim is to evaluate the performance of our modified ABC-SMC sampler and investigate model identifiability. Supplementary Figures: Marginal density plots of the unadjusted ABC posterior at N = 500, 1000 and 1500 given the observed empirical data.

### Supplementary Information

Below is the link to the electronic supplementary material.

**Supplementary S1: Pseudo-codes of exact simulation and (\tau)-leaping for the CTMC simulation model.** Here, we present the pseudo-codes for both the exact Stochastic Simulation Algorithm (SSA) and the hybrid (tau)-leaping algorithm, designed for our multidimensional CTMC stochastic simulation model. (pdf 7,244KB)

## Data Availability

The datasets and well-documented R codes or source files used in the current study have been made publicly available for reproducibility of results. To directly access these files, click here
