# Supplementary material for: Mathematical Modelling of Parasite Dynamics: A Stochastic Simulation-Based Approach and Parameter Estimation via Modified Sequential-Type Approximate Bayesian Computation
Source: Bull Math Biol. 2024 Apr 10;86(5):54. doi: 10.1007/s11538-024-01281-5 (PMC11006762; doi:10.1007/s11538-024-01281-5)
Supplement: Supplementary file 1 — Supplementary S1: Pseudo-codes of exact simulation and (\tau)-leaping for the CTMC simulation model. Here, we present the pseudo-codes for both the exact Stochastic Simulation Algorithm (SSA) and the hybrid (tau)-leaping algorithm, designed for our multidimensional CTMC stochastic simulation model. (pdf 7,244KB) [file 11538_2024_1281_MOESM1_ESM.pdf]

Mathematical modelling of parasite dynamics: A stochastic simulation-based approach and parameter estimation via modified sequential-type approximate Bayesian computation

Clement Twumasi[1,2,3,4*], Joanne Cable[4] and Andrey Pepelyshev[3*]

[1*]Nuffield Department of Medicine, University of Oxford, South Parks Road, Oxford, OX1 3SY, Oxfordshire, UK.

[2]School of Public Health, Imperial College London, 68 Wood Lane,London, W12 7RH, Greater London, UK.

[3*]School of Mathematics, Cardiff University, Senghennydd Road, Cardiff, CF24 4AG, South Glamorgan, UK.

[4]School of Biosciences, Cardiff University, Sir Martin Evans Building, Cardiff, CF10 3AX, South Glamorgan, UK.

1 Supplementary S1: Pseudo-codes of exact simulation and τ-leaping for the CTMC simulation model

This section provides the pseudo-codes for both the exact Stochastic Simulation Algorithm (SSA) and the hybrid τ-leaping algorithm (Algorithms 1 and 2) designed for the multidimensional Continuous-Time Markov Chain (CTMC) model, aimed at simulating the gyrodactylid infection dynamics of a single fish (based on the stochastic scheme defined by Table 1 in the main paper). All indices in the algorithms are defined in a manner consistent with the specifications outlined in the main paper.

Algorithm 1: Exact SSA of the simulation model (pseudo-code)

Input: A_0, I_0, J, $\mathbf{x}$, b_k, d_k, m, r, s, s_1, κ, ϵ_1, ϵ_2, ϵ_3, f, t, t_{final}, fish sex (z_{h1}), fish size (z_{h2}), fish stock (z_{h3}), parasite strain (g) and host survival status ($\mathbf{x}$).

Output: Number of parasites at each body region over time, $X_j(t) = \sum_{k=1}^{2} A_{j,k}^{(h)}(t)$ for fish h; host survival status (alive: $\mathbf{x} = 1$; dead: $\mathbf{x} = 2$)

1 **while** $t < t_{final}$ *and* $\mathbf{x} = 1$ **do**

2 Set initial time $t = t_0$; state $A_{1,1} = A_0 = 2$ & zero elsewhere; immune state $I_j = I_0 = 1$; host survival status $\mathbf{x} = 1$.

3 Calculate the total event rates $a_\delta\left(A_{j,k}^{(h)}\right)$ for all higher-level events $\delta = 1, 2, \cdots, 6$ such that: Birth $= A_{j,k}^{(h)} \times \left[1 - (A_{j,k}^{(h)}/(f_j \times z_{h2} \times \kappa))\right] \times b_{gk}(I_j)$,

Death $= A_{j,k}^{(h)} \times \left[1 - (A_{j,k}^{(h)}/(f_j \times z_{h2} \times \kappa))\right] \times d_{gi}$,

Forward movement $= A_{j,k}^{(h)} \times m(I_j) \times \epsilon_g$,

Backward movement $= A_{j,k}^{(h)} \times m(I_j) \times (1 - \epsilon_g)$,

Immune response $= \sum_{k=1}^{2} A_{j,k}^{(h)} \times r(z_{h1}, z_{h3})$,

Fish mortality $= \sum_j \sum_k A_{j,k}^{(h)} \times s(z_{h1}, z_{h2})$.

4 Compute the overall total rate, $a_0 = \sum_{\delta=1}^{6} a_\delta$, for higher-level events $\delta = 1, 2, \cdots, 6$ (from step 3).

5 Determine the higher-level events to occur at a host's body regions using a random number $\mathbf{u}$ from $Uniform(0, a_0)$ at a probability equal to $\frac{a_\delta}{a_0}$ such that:

6 **if** $\mathbf{u} < a_1$ **then**

7 update $A_{j,k} = A_{j,k} + 1$ (birth event occurs)

8 **end**

9

10 **if** $a_1 < \mathbf{u} < a_1 + a_2$ **then**

11 update $A_{j,k} = A_{j,k} - 1$ (death event occurs)

12 **end**

13

14 **if** $a_1 + a_2 < \mathbf{u} < a_1 + a_2 + a_3$ **then**

15 update $A_{j,k} = A_{j,k} - 1$, $A_{j+1,k} = A_{j+1,k} + 1$(forward movement event occurs)

16 **end**

17

18 **if** $a_1 + a_2 + a_3 < \mathbf{u} < a_1 + a_2 + a_3 + a_4$ **then**

19 update $A_{j-1,k} = A_{j-1,k} + 1$, $A_{j,k} = A_{j,k} - 1$ (backward movement event occurs)

20 **end**

21

22 **if** $a_1 + a_2 + a_3 + a_4 < \mathbf{u} < a_1 + a_2 + a_3 + a_4 + a_5$ **then**

23 set $I_j = 2$ (immune response event occurs)

24 **else**

25 set $\mathbf{x} = 2$ and **break** (host mortality event occurs)

26 **end**

27

28 Generate time increment τ_{SSA} from $Exponential(a_0)$, and update the time such that $t = t + \tau_{\text{SSA}}$.

29 Record $\left(X_j = \sum_{k=1}^{2} A_{j,k}^{(h)}, \mathbf{x}\right)$ at the desired discrete times for fish h at body region $j = 1, 2, 3, 4$.

30 **end**

Algorithm 2: Hybrid τ-leaping algorithm for simulation model (pseudo-code)

Input: A_0, I_0, J, $\mathbf{x}$, b_k, d_k, m_k, r, s, s_1, κ, ϵ_1, ϵ_2, ϵ_3, f, t, t_{final}, fish sex (z_{h1}), fish size (z_{h2}), fish stock (z_{h3}), parasite strain (g) and host survival status $(\mathbf{x})$.

Output: Number of parasites at each body region over time, $X_j(t) = \sum_{k=1}^{2} A_{j,k}^{(h)}(t)$ for fish h; host survival status (alive: $\mathbf{x} = 1$; dead: $\mathbf{x} = 2$)

1 **while** $t < t_{final}$ *and* $\mathbf{x} = 1$ **do**
2   Set initial time $t = t_0$; state $A_{1,1} = A_0 = 2$ & zero elsewhere; immune state $I_j = I_0 = 1$; host survival status $\mathbf{x} = 1$.
3   Calculate the total event rates $a_\delta\left(A_{j,k}^{(h)}\right)$ for all higher-level events $\delta = 1, 2, \cdots, 6$ such that: Birth $= A_{j,k}^{(h)} \times \left[1 - (A_{j,k}^{(h)}/(f_j \times z_{h2} \times \kappa))\right] \times b_{gk}(I_j)$,
Death $= A_{j,k}^{(h)} \times \left[1 - (A_{j,k}^{(h)}/(f_j \times z_{h2} \times \kappa))\right] \times d_{gi}$,
Forward movement $= A_{j,k}^{(h)} \times m(I_j) \times \epsilon_g$,
Backward movement $= A_{j,k}^{(h)} \times m(I_j) \times (1 - \epsilon_g)$,
Immune response $= \sum_{k=1}^{2} A_{j,k}^{(h)} \times r(z_{h1}, z_{h3})$,
Fish mortality $= \sum_j \sum_k A_{j,k}^{(h)} \times s(z_{h1}, z_{h2})$.
4   Compute the overall total rate, $a_0 = \sum_{\delta=1}^{6} a_\delta$, for higher-level events $\delta = 1, 2, \cdots, 6$ (from step 3).
5   Compute the leap size τ_{leap}.
6   **if** $\tau_{leap} > \frac{1}{10a_0}$ **then**
7   set $t = t + \tau_{\text{leap}}$ and determine the higher-level events to occur at a host's body regions using a random number $\mathbf{u}$ from $Uniform(0, a_0)$ such that:
8   **if** $\mathbf{u} < a_6$ **then**
9   Set $\mathbf{x} = 2$ and **break** (host mortality event occurs)
10   **end**
11
12   **if** $a_6 < \mathbf{u} < a_5 + a_6$ **then**
13   Set $I_j = 2$ (immune response event occurs)
14   **end**
15
16   **if** $a_5 + a_6 < \mathbf{u} < a_1 + a_2 + a_3 + a_5 + a_6$ **then**
17   update $A_{j,k} = A_{j,k} + \sum_\delta v_\delta P(a_\delta, \tau_{\text{leap}})$ where $P \sim Poisson(a_\delta \tau_{\text{leap}})$ and v_δ is state-change vector (birth, death and forward movement events occur)
18   **else**
19   update $A_{j,k} = A_{j,k} + \sum_\delta v_\delta P(a_\delta, \tau_{\text{leap}})$ (birth, death and backward movement events occur)
20   **end**
21   **else**
22   **end**
23   Execute exact SSA (Algorithm 1)
24   Record $\left(X_j = \sum_{k=1}^{2} A_{j,k}^{(h)}, \mathbf{x}\right)$ at the desired discrete times for fish h and $j = 1, 2, 3, 4$.
25 **end**

2 Supplementary S2: Determining an error bound for the Hybrid τ-leaping simulation model

In this section, we present results regarding the impact of the error bound, denoted as ϵ $(0 < \epsilon \ll 1)$, on the simulation output for the hybrid τ-leaping simulation model. We explore the trade-off between simulation accuracy and computational speed under fixed parameter values (Table 1). The analysis is based on 100 different simulation realisations or repetitions, each corresponding to the nine observed parasite-fish groups (given fish sex, fish size, fish stock, and parasite strain). The simulation accuracy is quantified using the mean square error, given by equation 1, which is calculated based on the mean (simulated) parasite numbers over time (day 1 to 17) from both the exact SSA (Algorithm 1) and the hybrid τ-leaping algorithm (Algorithm 2). We consider 10 different error bound values, namely, $\epsilon = 0.002, 0.004, 0.006, 0.008, 0.01, 0.02, 0.04, 0.06, 0.08$, and 0.1.; such that

$$\text{MSE}\left(\bar{X}^{(g)}_{\text{leap}}(t), \bar{X}^{(g)}_{\text{SSA}}(t)\right) = \frac{1}{100}\sum_{r=1}^{100}\left(\bar{X}^{(g)}_{\text{leap},r}(t) - \bar{X}^{(g)}_{\text{SSA},r}(t)\right)^2, \quad (1)$$

where $\bar{X}^{(g)}_{\text{leap},r}(t)$ and $\bar{X}^{(g)}_{\text{SSA},r}(t)$ are the mean parasite numbers over time t from hybrid $\tau - leaping$ and exact SSA, respectively; across each of the nine parasite-fish groups (g) and simulation realisation (r). The respective confidence intervals of the mean over time between the two simulation methods were also compared at $0 < \epsilon < 0.1$ for each parasite-fish group over time. The simulation speed was quantified by the computational time (computer's CPU time measured in seconds).

The investigation revealed a reduction in simulation accuracy in the hybrid τ-leaping algorithm as the error bound (ϵ) increased from $\epsilon = 0.002$ to $\epsilon = 0.1$ (refer to Figure 1). Notably, mean parasite numbers from the hybrid τ-leaping simulations remained relatively consistent with the exact Stochastic Simulation Algorithm (SSA) at error bounds within the range $0.002 \leq \epsilon \leq 0.01$ across the nine parasite-fish groups (see Figures 2–6), including their respective confidence intervals. However, as the error bound increased beyond $\epsilon = 0.02$, the performance of the τ-leaping algorithm deteriorated across the parasite-fish groups, particularly

approaching $\epsilon = 0.1$ (see Figures 7–11). Figure 12 indicates that at $\epsilon = 0.008$ or $\epsilon = 0.01$, the τ-leaping algorithm exhibited relatively fast performance, although not significantly different from the computational time of the exact SSA. This unexpected observation may be attributed to either the number of simulation repetitions (100 repetitions) or the relatively small number of parasites over time at the pre-specified parameter values (randomly chosen) such that the leaping condition was not frequently met. Based on considerations of simulation accuracy, $\epsilon = 0.002$ remains a reasonable choice for the error bound in subsequent simulations using the multidimensional stochastic model.

Table 1: Fixed parameter values for choosing an error bound

Parameters	Fixed values
**Base simulation parameters**	
b_{11}	0.668
b_{12}	0.018
b_{21}	0.668
b_{22}	0.018
b_{31}	0.668
b_{32}	0.018
d_{11}	0.008
d_{12}	0.071
d_{21}	0.008
d_{22}	0.071
d_{31}	0.008
d_{32}	0.071
m	0.083
r	0.001
s	0.009
κ	182
**Additional simulation parameters**	
ϵ_1	0.545
ϵ_2	0.333
ϵ_3	0.001
r_1	0.351
r_2	0.196
r_3	0.994
s_1	0.041

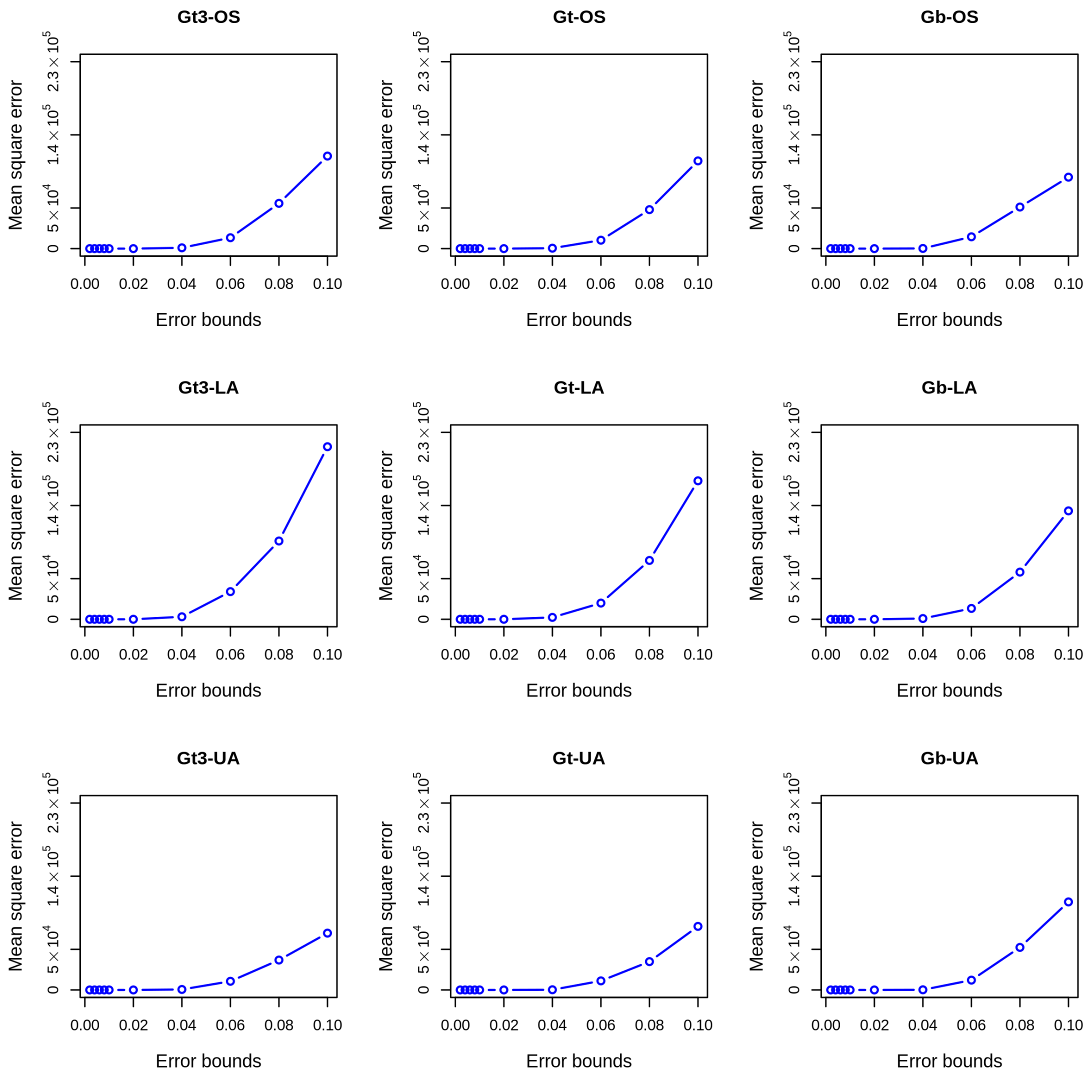

Fig. 1: Mean square error from the Hybrid τ-leaping algorithm at different error bounds.

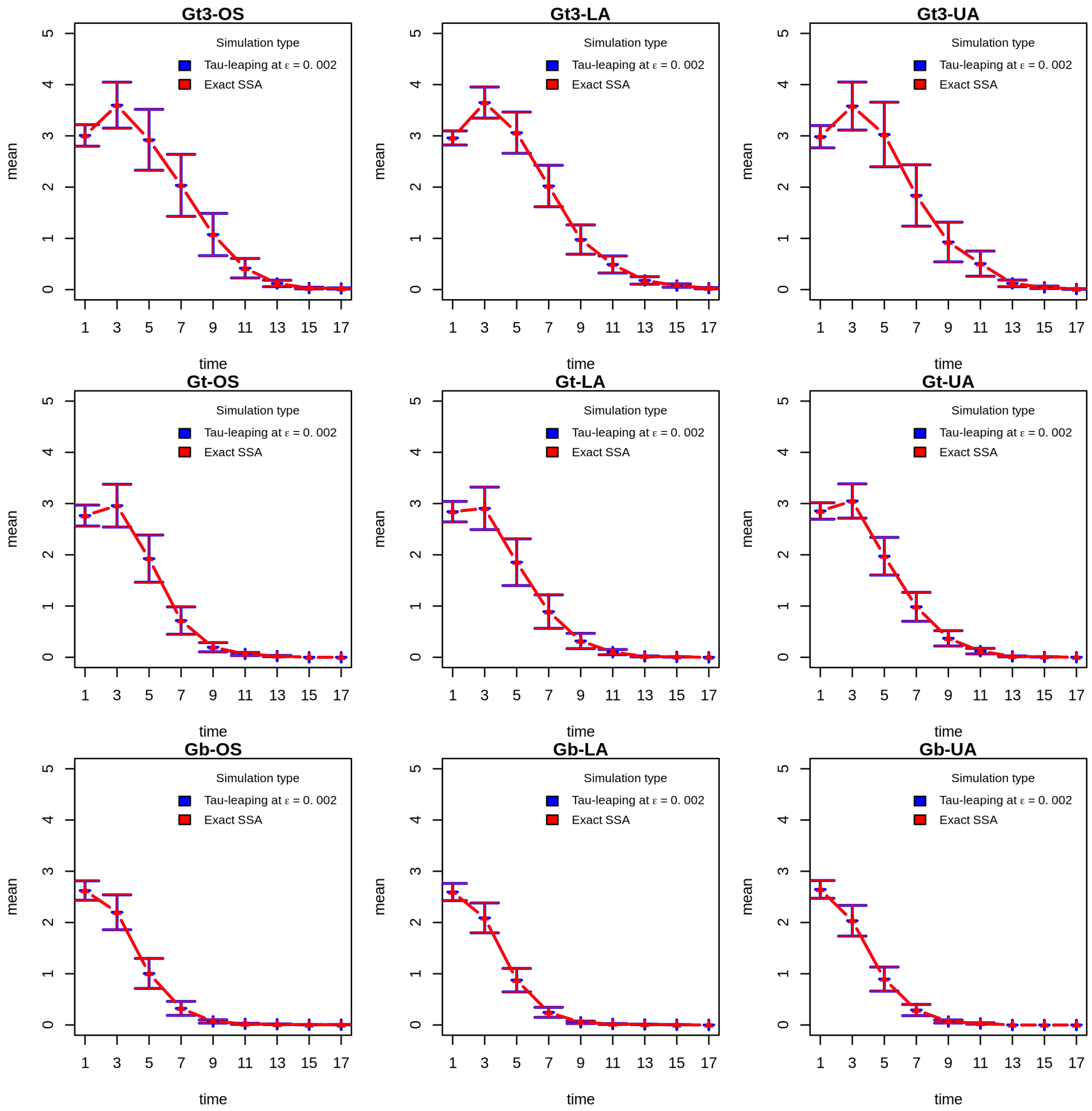

Fig. 2: Mean comparison between exact SSA and Hybrid τ-leaping simulations at $\epsilon = 0.002$.

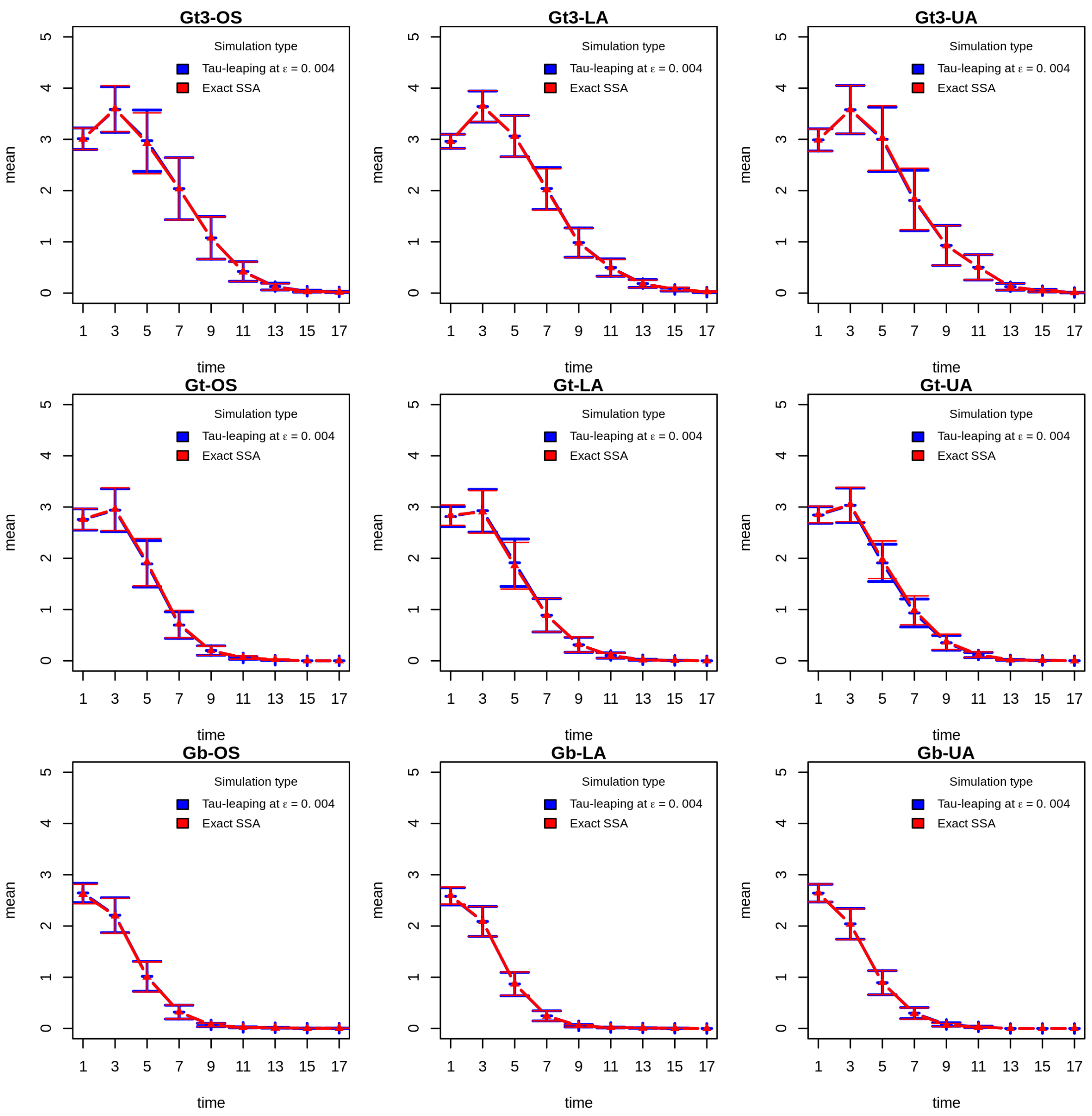

Fig. 3: Mean comparison between exact SSA and Hybrid τ-leaping simulations at $\epsilon = 0.004$.

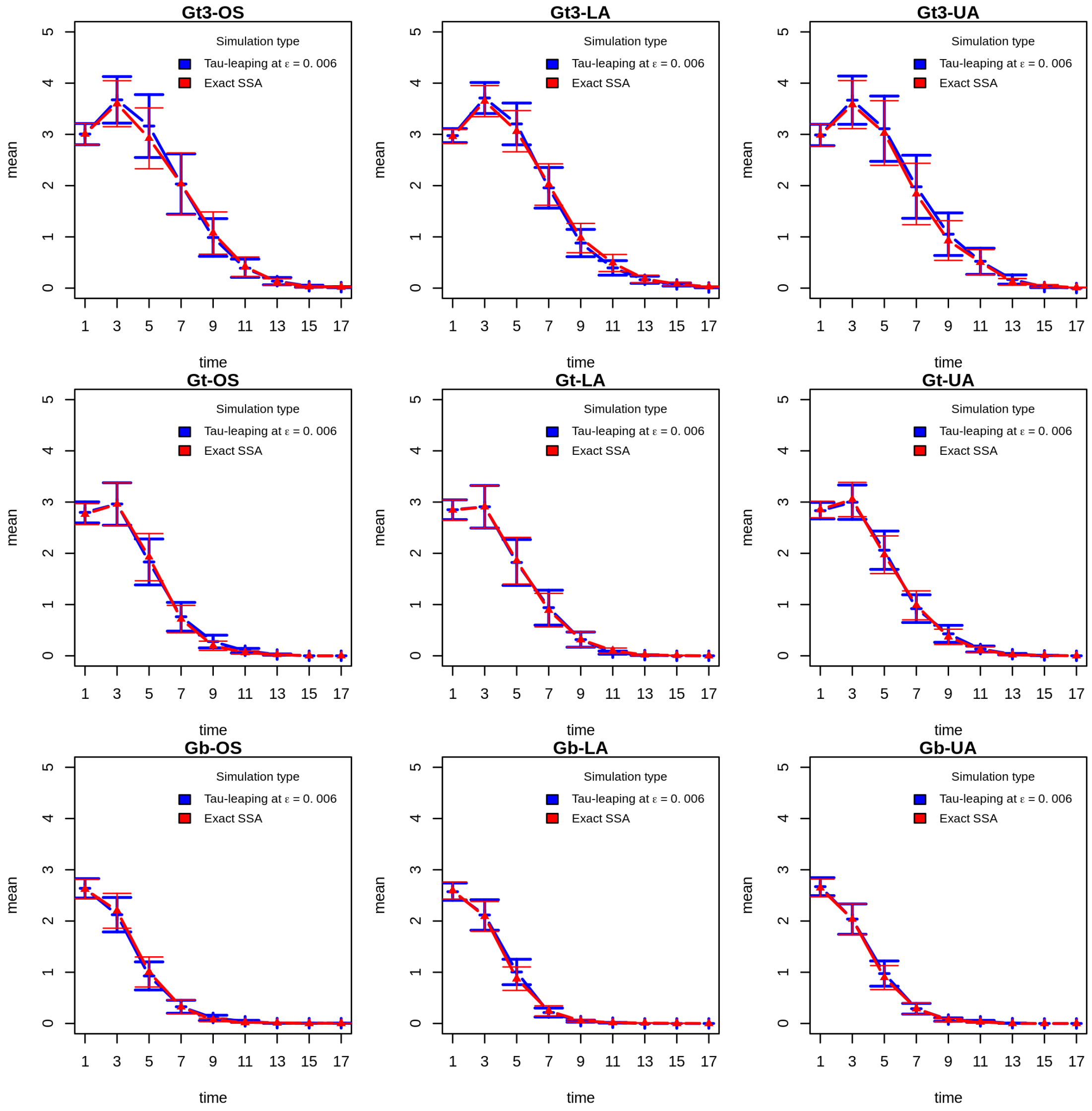

Fig. 4: Mean comparison between exact SSA and Hybrid τ-leaping simulations at $\epsilon = 0.006$.

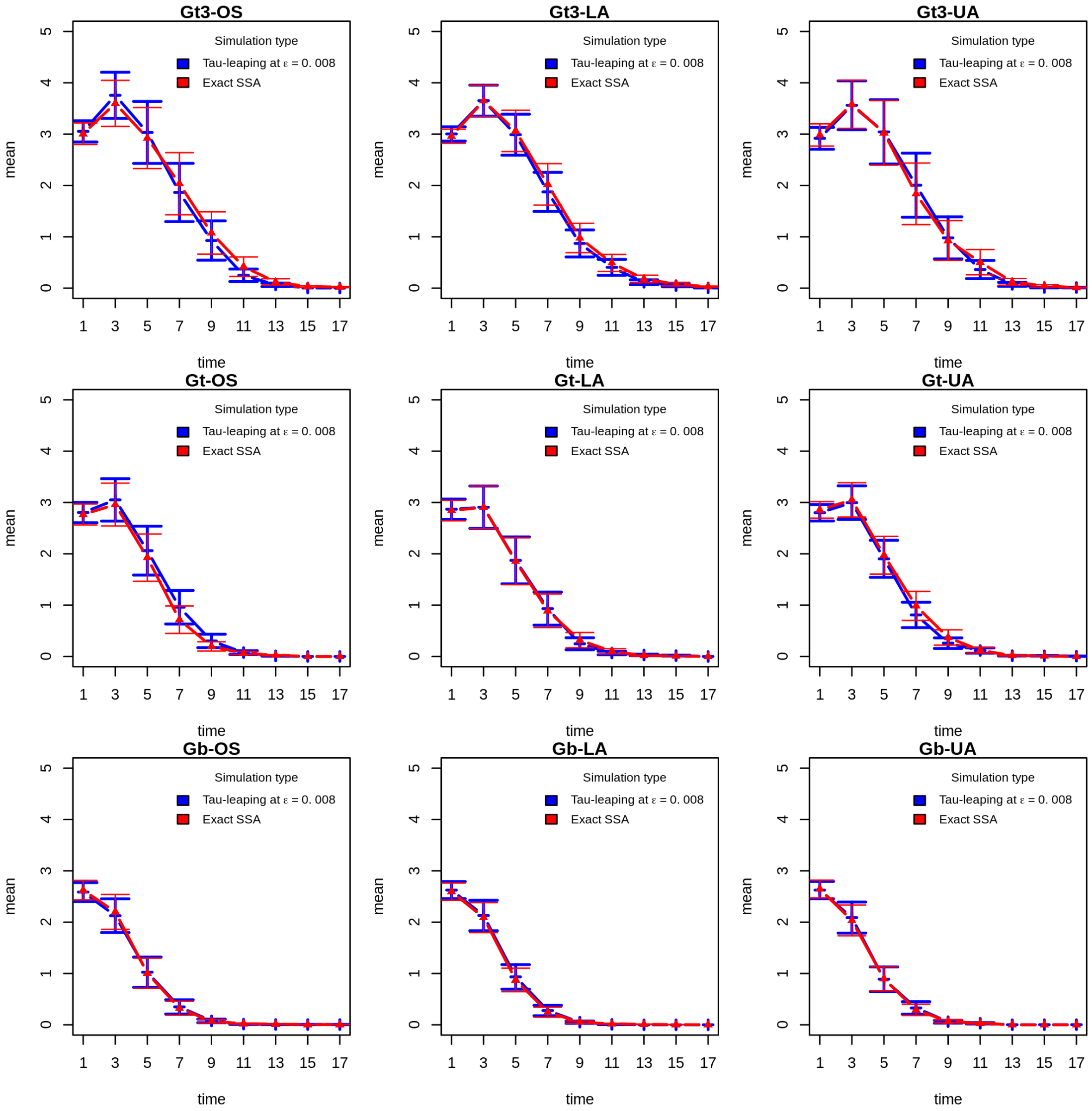

Fig. 5: Mean comparison between exact SSA and Hybrid τ-leaping simulations at $\epsilon = 0.008$.

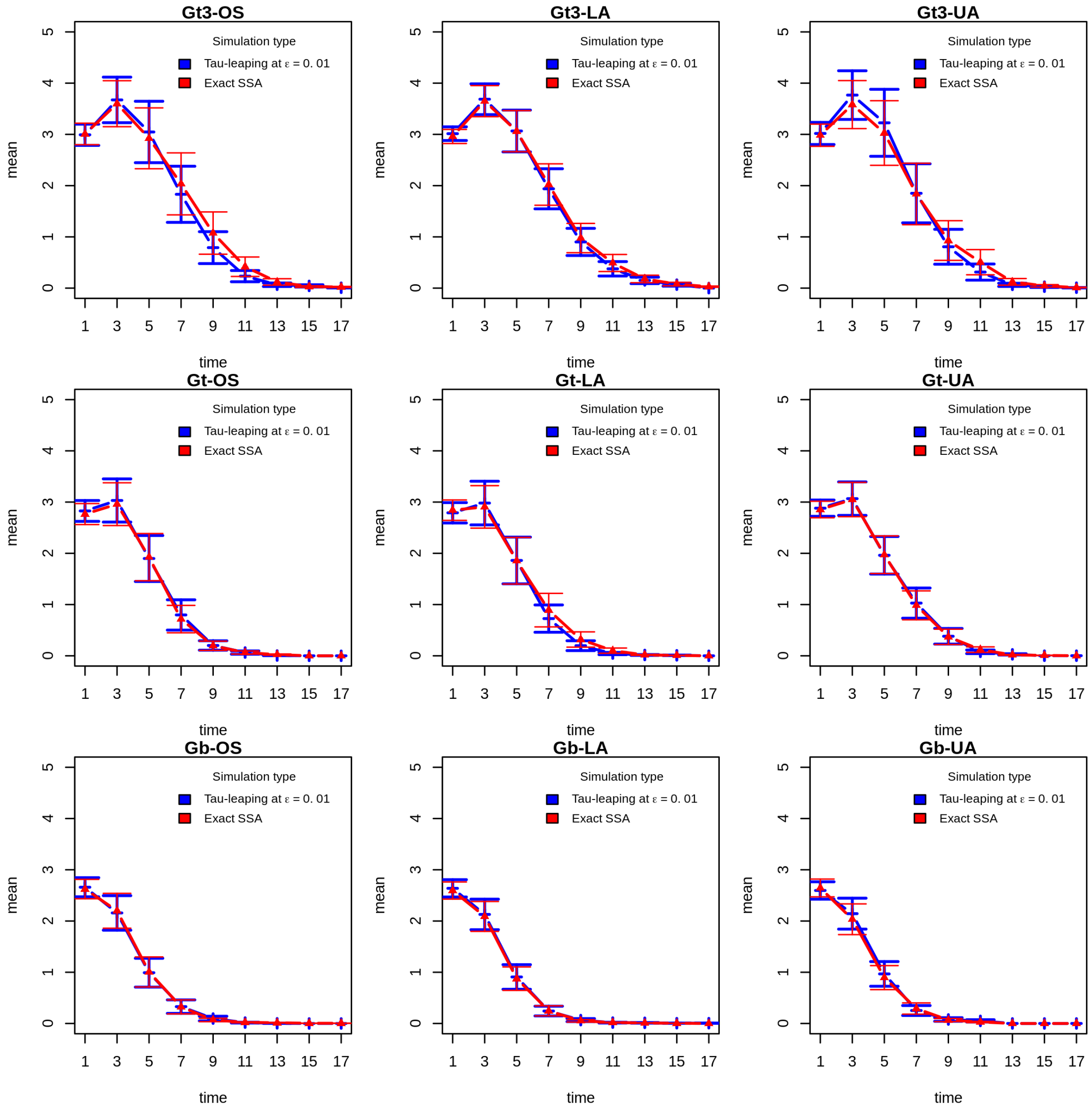

Fig. 6: Mean comparison between exact SSA and Hybrid τ-leaping simulations at $\epsilon = 0.01$.

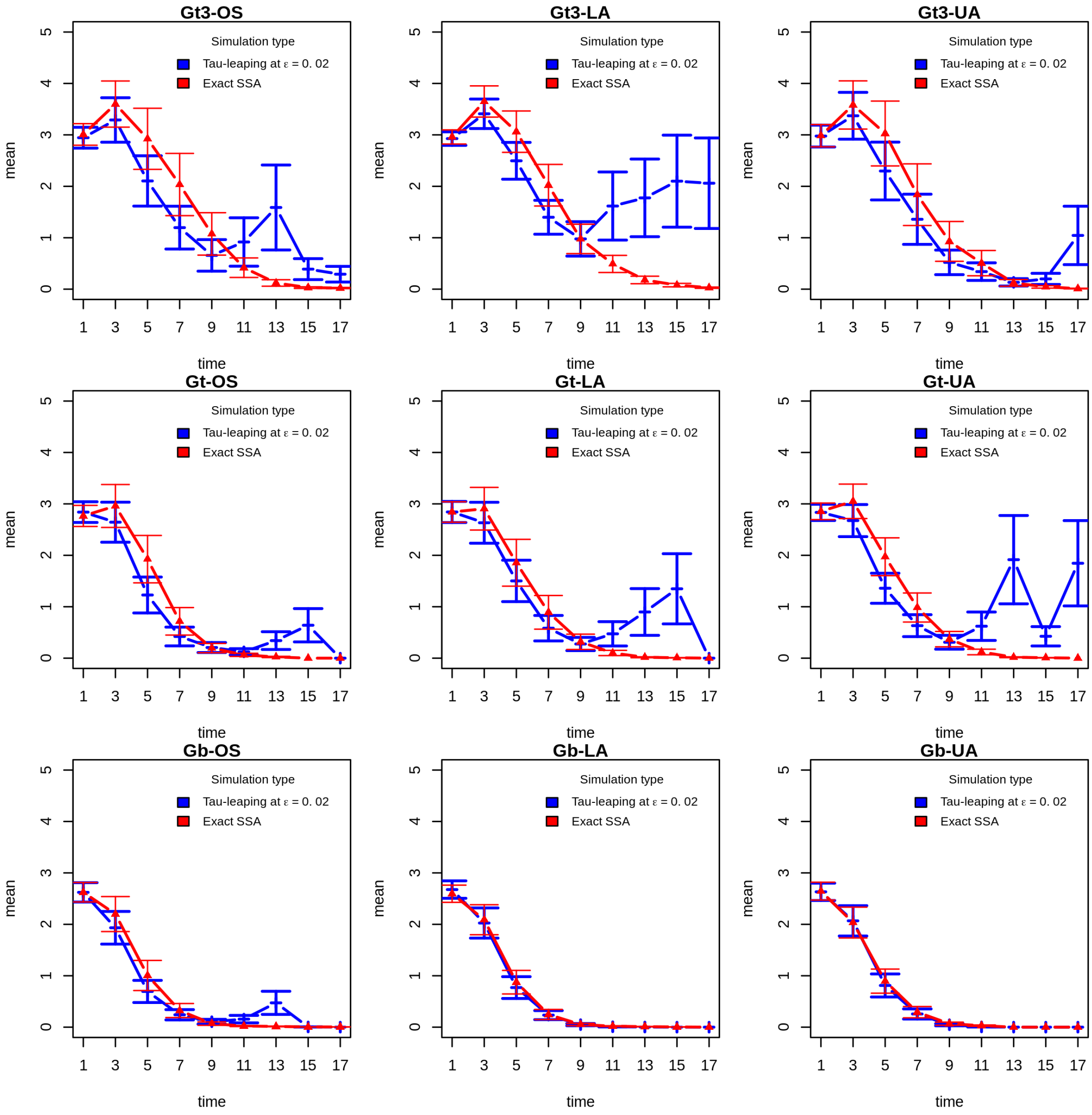

Fig. 7: Mean comparison between exact SSA and Hybrid τ-leaping simulations at $\epsilon = 0.02$.

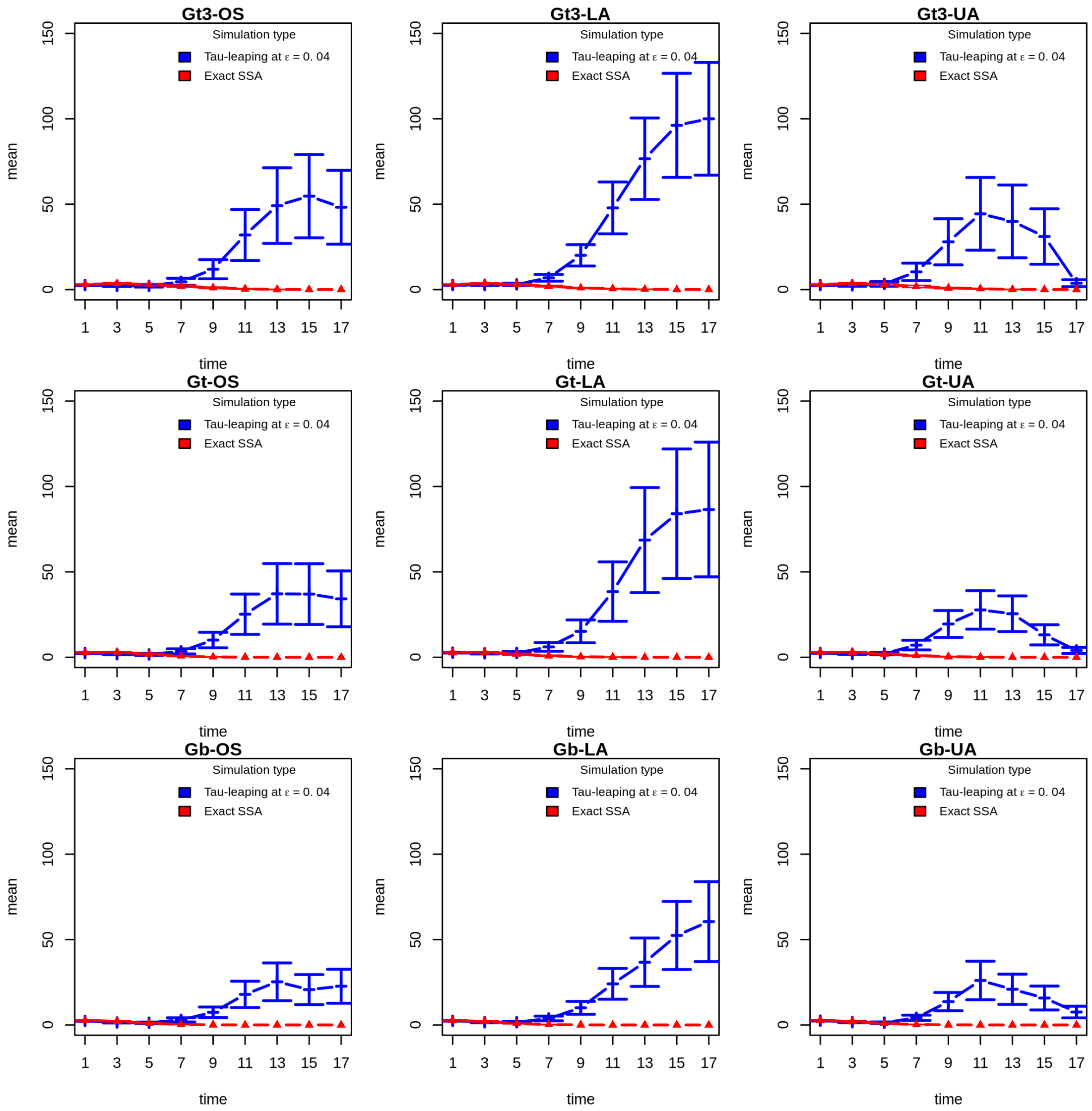

Fig. 8: Mean comparison between exact SSA and Hybrid τ-leaping simulations at $\epsilon = 0.04$.

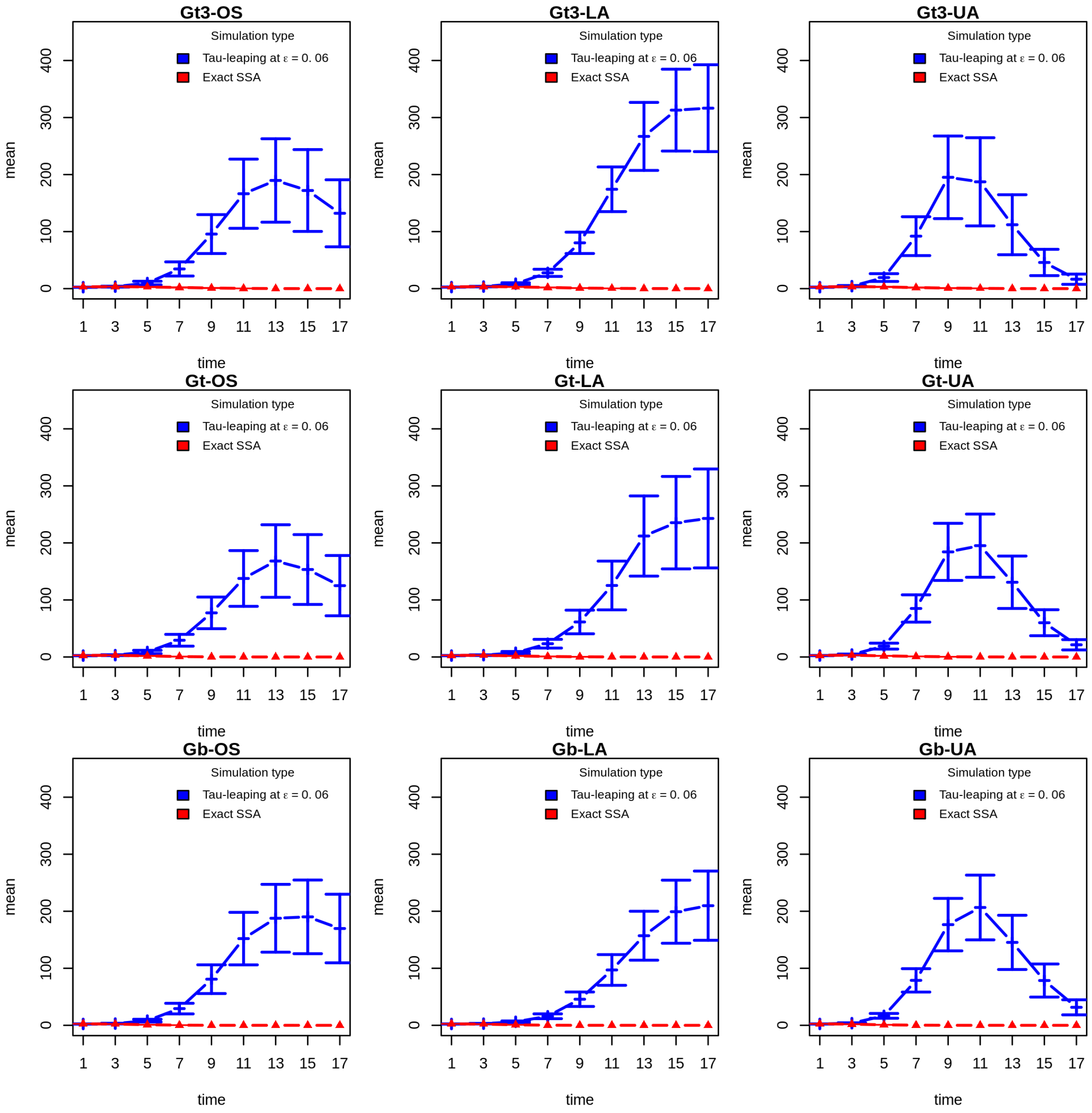

Fig. 9: Mean comparison between exact SSA and Hybrid τ-leaping simulations at $\epsilon = 0.06$.

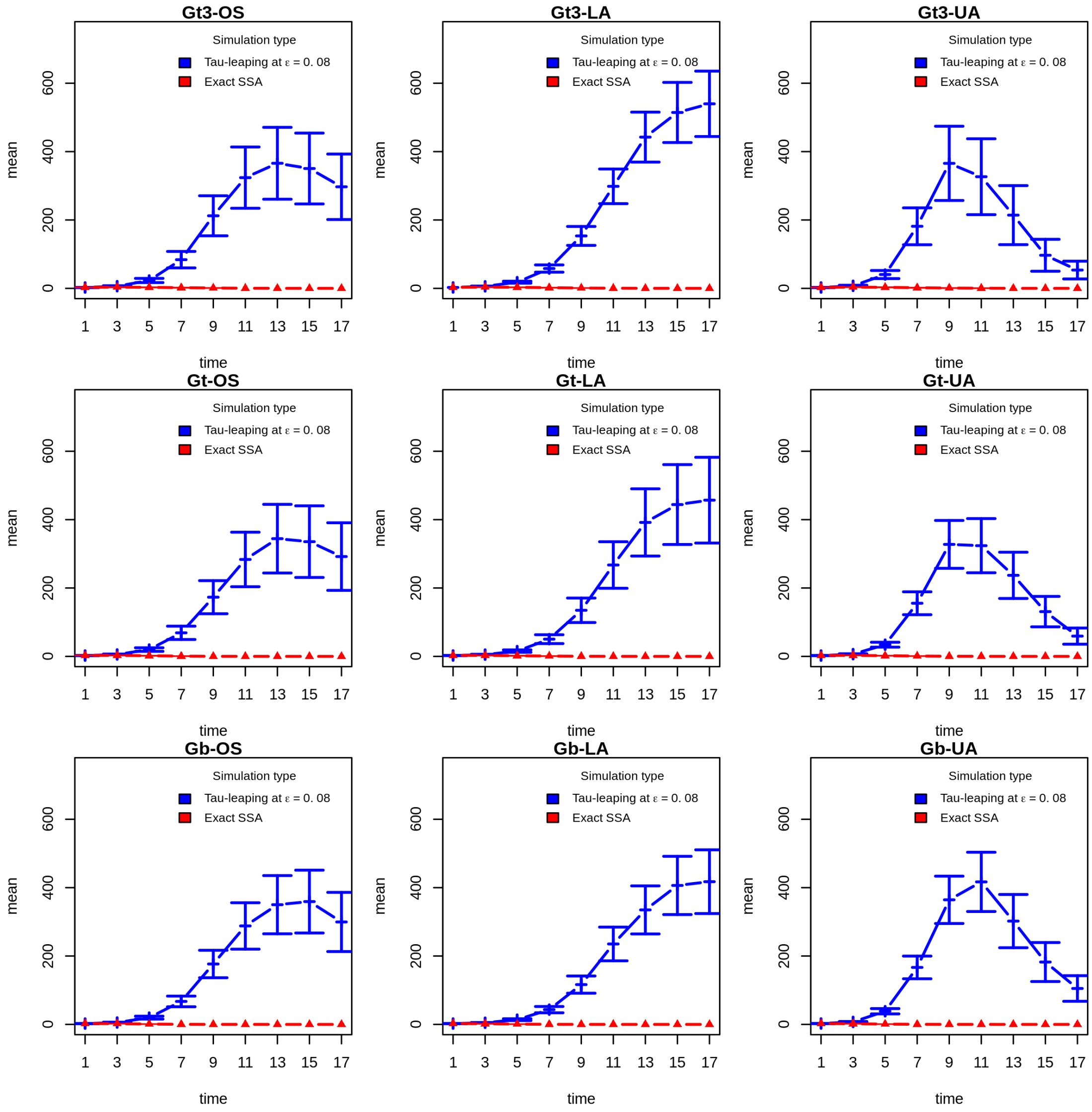

Fig. 10: Mean comparison between exact SSA and Hybrid τ-leaping simulations at $\epsilon = 0.08$.

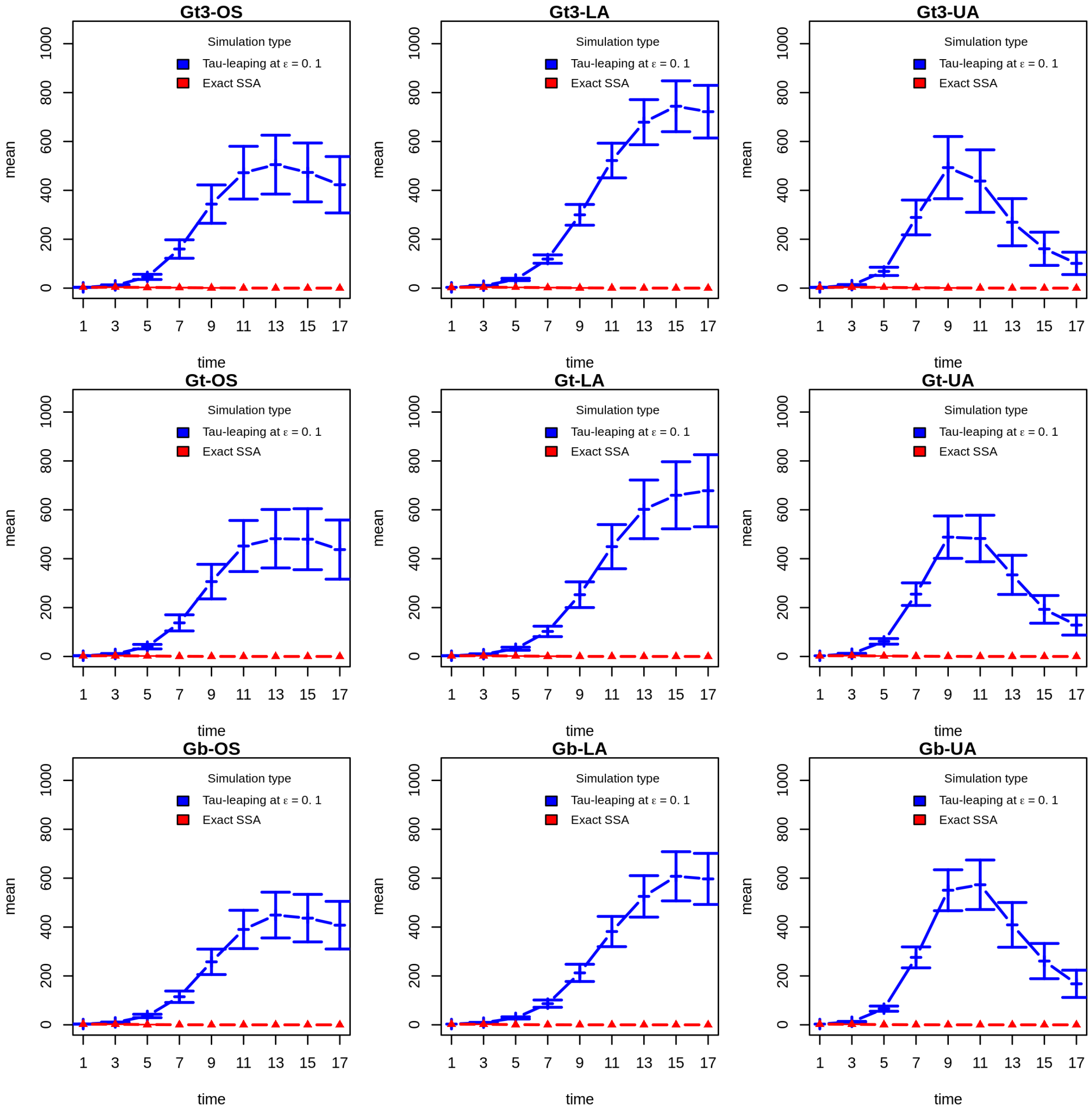

Fig. 11: Mean comparison between exact SSA and Hybrid τ-leaping simulations at $\epsilon = 0.1$.

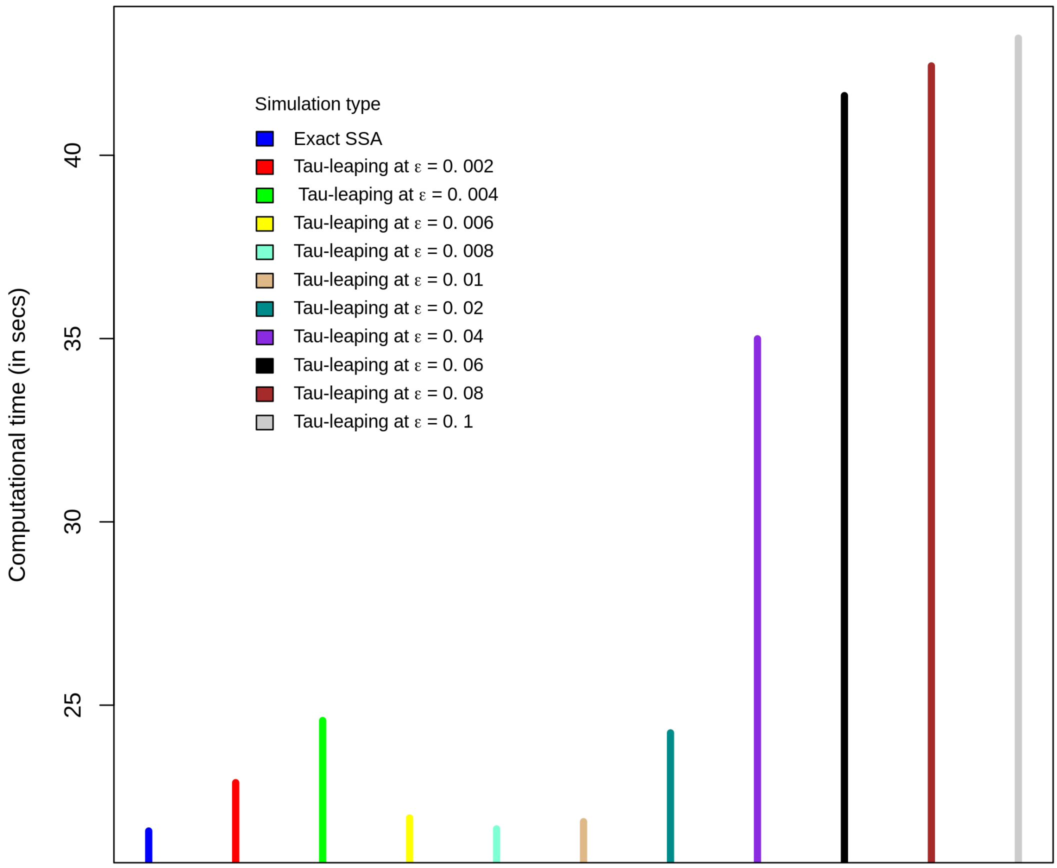

Fig. 12: Comparison between computational time between exact SSA and Hybrid τ-leaping simulation at different error bounds $0 < \epsilon \leq 0.1$.

3 Supplementary S3: Projection of parasite numbers after fish mortality

This section presents the regression function used to project parasite numbers after host mortality in the CTMC simulation model defined in the main paper.

Now, when a host dies at time $t \geq 1$, it is often observed in most biological systems that the entire parasite population on the host becomes extinct before time $t + 1$. However, a study on survival and behaviour of *Gyrodactylus turnbulli* and *G. bullatarudis* on dead fish revealed that under certain conditions, such as high parasite abundance at the time of death, these parasites might survive on a dead host for a while [2]. Thus, after premature host mortality (before the end of the infection period), we cannot assume that there is parasite population extinction in all body regions, depending on the parasite abundance at the time of death.

3.1 The projection function

During such realisations when an infected fish dies before the end of the observation period in the CTMC simulation model or observed empirical data, we use an estimated linear regression function (given by equation 6) based on the parasite data before host mortality, to linearly project the parasite numbers till the end of the observation period to aid in ABC implementation (by assigning more weight to the most recent data before host death as in a case of missing value imputation assuming at least missing-at-random). Furthermore, we minimise the projection estimation error by assigning more weight to the most recent outcomes or data prior to host mortality based on equations 2–4. Thus, the parasite population projection after host mortality is used to aid in the summary statistics computation for ABC fitting of our stochastic model; specifically, during the calculation of other components of the summary statistics such as the log counts of parasites over time till day 17, and weights of the Wasserstein 1-D distance metric between body regions of the host. We propose a linear function to estimate missing values after fish mortality till the end of the observation period:

Let y_t= total parasites at time t, $z_t = \log(y_t)$, k be the time before fish mortality, α a regression parameter and $\gamma \in (0, 1)$, a tuning parameter. Given $z_1, z_2, z_3, \cdots, z_k$, we want to estimate or project for $\hat{z}_{k+1}, \cdots, \hat{z}_9$. Now, let the proposed least squares

regression equation, denoted by $m(t)$, be defined as given by equation 2;

$$m(t) = (k - t)\alpha + z_k \tag{2}$$

with the estimate of the regression parameter (α) determined such that

$$\hat{\alpha} = \min \sum_t (m(t) - z_t)^2 \gamma^{k-t}. \tag{3}$$

Thus, the prediction of z_i for $i = k+1, k+2, \cdots, 9$ can be estimated using equation 4

$$\hat{z}_{k+i} = m(k+i) = -i \times \hat{\alpha} + z_k, \tag{4}$$

where i is the number of predictions across time. Then,

$$y_{k+i} = \exp(\hat{z}_{k+i}). \tag{5}$$

Hence, also letting $y_{t,u}$ be parasite at location u and time t implies the expected projections are defined such that

$$\hat{y}_{k+i,u} = \hat{y}_{k+i} \times \frac{y_{k,u}}{y_k}. \tag{6}$$

Remark. The estimate of the regression parameter (α) defined by equation 3 can be derived explicitly using Theorem 1 (for the theorem and its mathematical proof, see section 3.2). Due to the high computational demand in simulating data from our complex stochastic model as well as implementing our weighted-iterative ABC for model fitting (especially during the computation of the high-dimensional summary statistics), we tuned or set the value of γ at ≥ 0.9 (based on all observed fish that prematurely died), which we found to be a reasonable choice based on some heuristic technique which achieved some degree of convergence when estimating α (defined by Theorem 1 under section 3.2) at varying γ values ranging from 0 to 1 across all the different parasite-host groups. Figure 13 shows the convergence effect of varying values of the tuning parameter $\gamma \in (0, 1)$ on the estimate of

the regression coefficient α (defined by equation 7) across all parasite-fish groups (based on all observed fish that prematurely died as indicated by different colours).

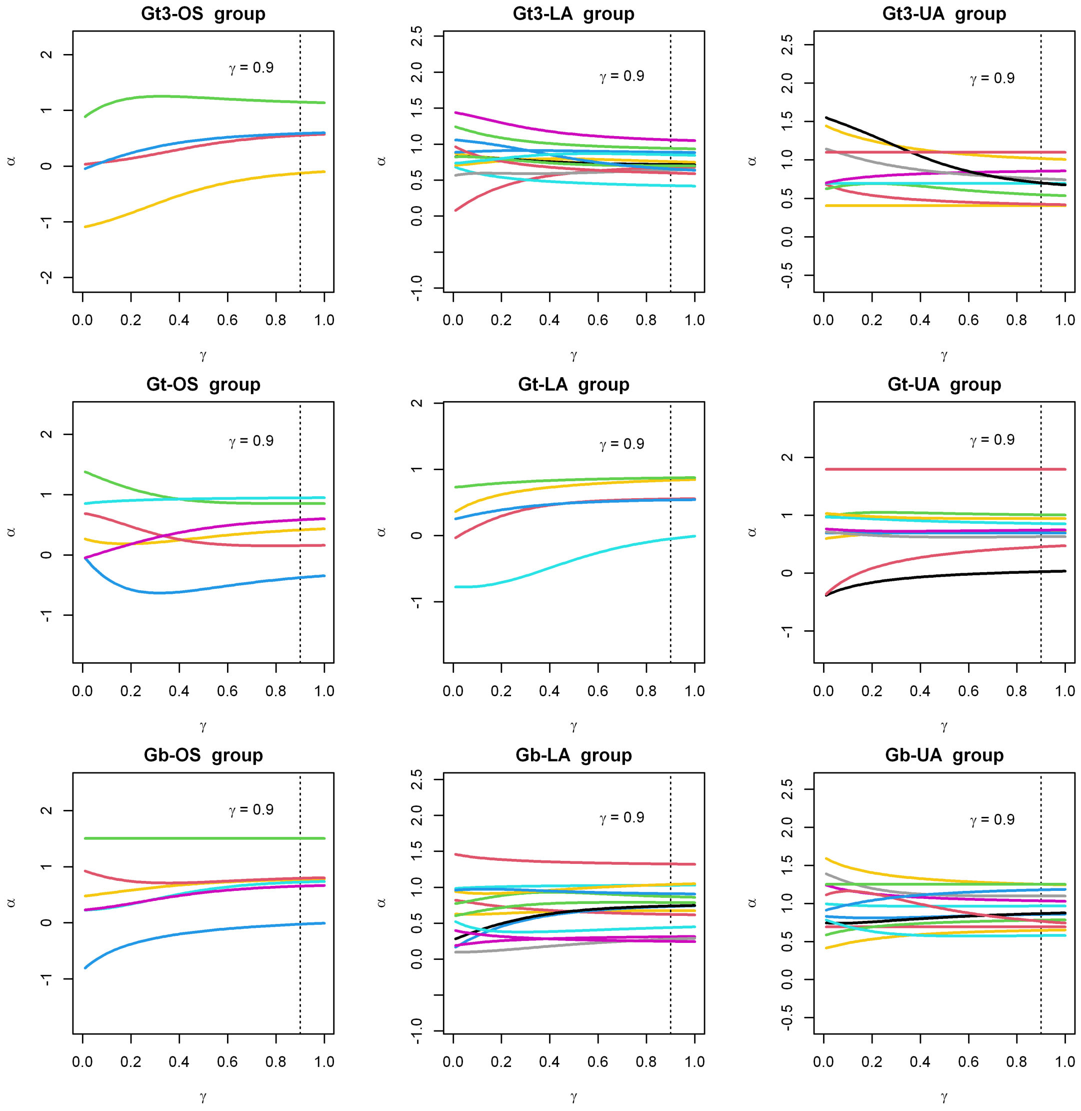

Fig. 13: Effect of varying values of the tuning parameter $\gamma \in (0, 1)$ on the estimate of the regression coefficient α (with different colours indicating all observed fish that prematurely died).

3.2 Derivation of an exact least squares estimator of the regression parameter α (defined by equation 3) based on Theorem 1

Theorem 1. ***(Least squares estimate of the regression parameter)***
The exact least squares estimate of the regression parameter α based on the linear regression equation 2 is given as:

$$\hat{\alpha} = \frac{\sum_{t=1}^{k-1}(z_t - z_k)(k-t)\gamma^{k-t}}{\sum_{t=1}^{k-1}(k-t)^2\gamma^{k-t}}. \tag{7}$$

Proof of Theorem 1 .

Suppose $m(t) = (k-t)\alpha + z_k$, $\alpha = \sum_t (m(t) - z_t)^2\gamma^{k-t}$ whilst fixing γ, and the loss function

$$L(\alpha, \gamma) = \min_{\alpha} \sum_t (m(t) - z_t)^2\gamma^{k-t}. \tag{8}$$

Differentiating equation 8 with respect to α and setting to zero gives

$$\frac{\partial L}{\partial \alpha} = 2\sum_{t=1}^{k-1}\{(k-t)\alpha + z_k - z_t\}\gamma^{k-t}(k-t) = 0.$$

This implies

$$\sum_{t=1}^{k-1}(k-t)^2\alpha\gamma^{k-t} + \sum_{t=1}^{k-1}(z_k - z_t)\alpha\gamma^{k-t}(k-t) = 0,$$

where

$$\alpha\sum_{t=1}^{k-1}(k-t)^2\gamma^{k-t} = -\sum_{t=1}^{k-1}(z_k - z_t)\alpha\gamma^{k-t}(k-t).$$

Therefore, solving for α gives the required equation 7 such that

$$\hat{\alpha} = -\frac{\sum_{t=1}^{k-1}(z_k - z_t)(k-t)\gamma^{k-t}}{\sum_{t=1}^{k-1}(k-t)^2\gamma^{k-t}} = \frac{\sum_{t=1}^{k-1}(z_t - z_k)(k-t)\gamma^{k-t}}{\sum_{t=1}^{k-1}(k-t)^2\gamma^{k-t}}, \gamma \in (0,1).$$ Q. E. D.

4 Supplementary S4: Assessing the modified ABC and regression adjustment using a numerical experiment

4.1 Introduction

In this section, we present the results of a numerical experiment using our stochastic simulation model under predetermined parameter settings and the proposed ABC methods. The objective is to assess the effectiveness of our modified ABC-SMC sampler and explore the identifiability of the new stochastic model for the gyrodactylid-fish system. From an unrelated toy model (with multivariate normal likelihood and known analytical posterior distribution), it has already been shown from works by Twumasi [4, pp. 186-203] that our modified ABC-SMC algorithm (i.e., weighted-iterative ABC) resulted in mutually compatible posterior approximations at the different values of Monte Carlo sample size or number of particle draws, N (for $500 \leq N \leq 5000$), based on a pre-specified tolerance and ABC time steps of size 10. Thus, it was inferred that the resulting posterior from the modified ABC-SMC algorithm is independent of N, and the degree of variability in the posterior distributions is not significantly different, irrespective of the number of particles drawn from the importance distribution.

Twumasi [4] also found from the simple simulation experiment that the computational cost (or cost in time) of the modified ABC-SMC sampler increases quadratically as the number of proposal draws increases from $N = 500$ to $N = 5000$ during the ABC fitting of the toy model (run in parallel using over 20 CPU cores of a multi-core processor). Based on these findings, it suggests that it will be cost-effective to fit the complex stochastic simulation (described in the main paper) at any smaller values $N \geq 500$ due to the high computational cost associated with simulations from our complex stochastic model (especially during realisations of parasite population explosion), estimation of the multidimensional summary statistics (across the entire host population), and the potential cost of implementing the modified ABC algorithm at higher values of $N \gg 500$.

Generally, there was no significant difference in the accuracy of the unadjusted and ridge-adjusted posterior mean estimates (for instance) at the different values of N, and the true posterior mean estimates were found in their respective estimated credible intervals. However, the mean square error (MSE) of the adjusted posterior mean based on the proposed regression correction (with L2 regularisation) resulted in a relatively smaller MSE and credible interval width most of the time (especially at $N \leq 1000$). In the main paper, an ABC regression adjustment method based on an L1 regularisation procedure has also been proposed for comparison with the ridge-adjusted method. Section 4.2 presents ABC results from a different numerical experiment based on our stochastic simulation model at $N = 1500$ given pseudo-observed data (generated from our simulation model).

4.2 Results of the numerical experiment based on our stochastic simulation model

4.2.1 ABC fitting based on pseudo-observed data

Pseudo-observed data were simulated from our stochastic model at predefined parameter values (on a logarithmic scale). The model was then fitted to the pseudo-observed data using our modified ABC-SMC algorithm (i.e., weighted-iterative ABC). Figures 14–16 illustrate a consistent reduction in the quantiles of ABC distances (which quantify the discrepancy between simulated and pseudo-observed data) over ABC time steps at any predefined quantile or percentile value (at $500 \leq N \leq 1500$). This decreasing pattern indicates an enhanced performance or convergence of the modified ABC-SMC algorithm towards a good approximation of the true posterior distribution. This improvement aligns with findings from the unadjusted ABC marginal density plot (Figure 17), suggesting that as the ABC sequential updates unfold, the simulated data progressively gets closer to the pseudo-observed data, reflecting posterior convergence over time.

The resulting posterior was further adjusted using the two proposed penalised regression-adjusted methods (which employ L1 and L2 regularisation procedures). Figure 18 indicates the presence of high multicollinearity between some of the regression predictors (in the neighbourhood of the observed summaries);

confirming why the standard Beaumont et al. [1] local-linear regression (with heteroscedastic errors) is impossible to implement in this instance (as a result of non-invertible matrices in its estimator). Figures 19–22 are marginal density plots comparing the unadjusted posterior and the two regression-adjusted ABC methods.

In section 4.2.2, results on the accuracy of the posterior estimates based on the proposed ABC methods are presented. Further multivariate posterior predictive checks are performed with Principal Component Analysis (PCA) under section 4.2.3.

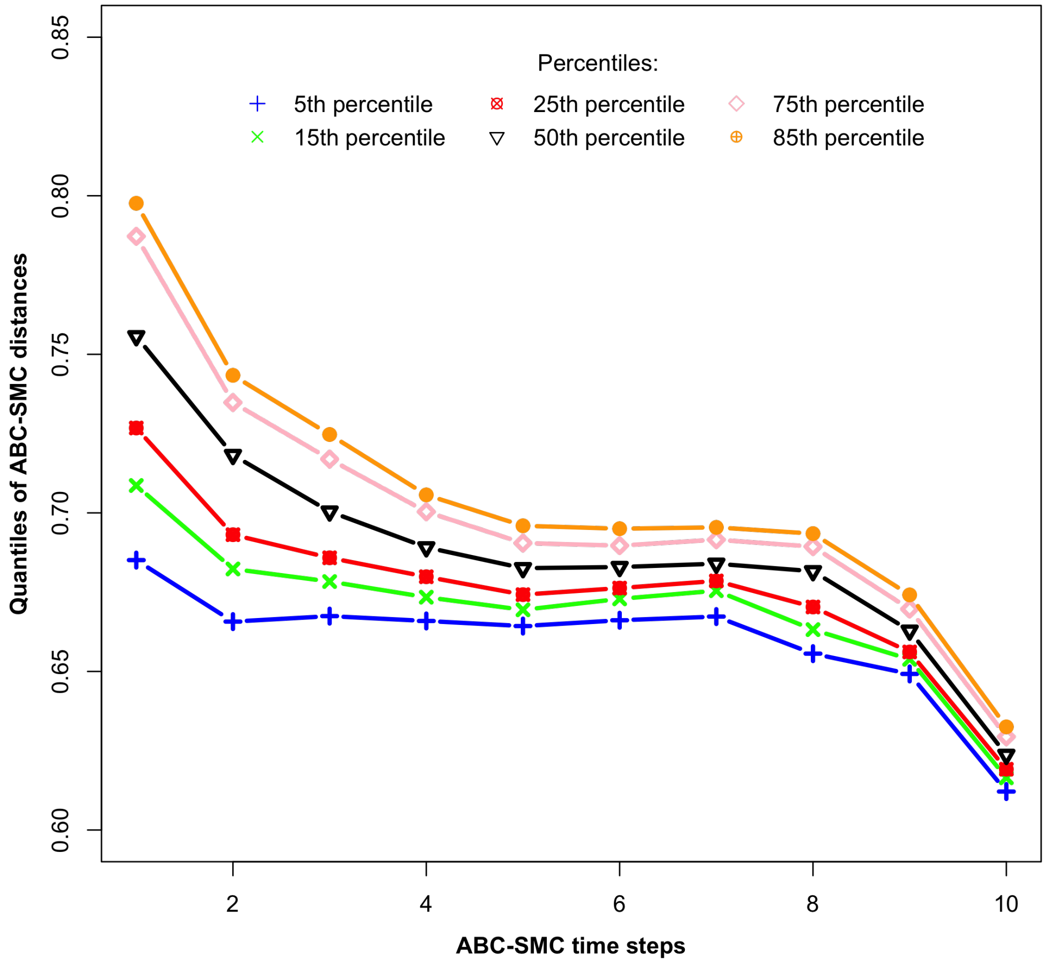

Fig. 14: Plot of distance quantiles at the 10 ABC time steps at N=500.

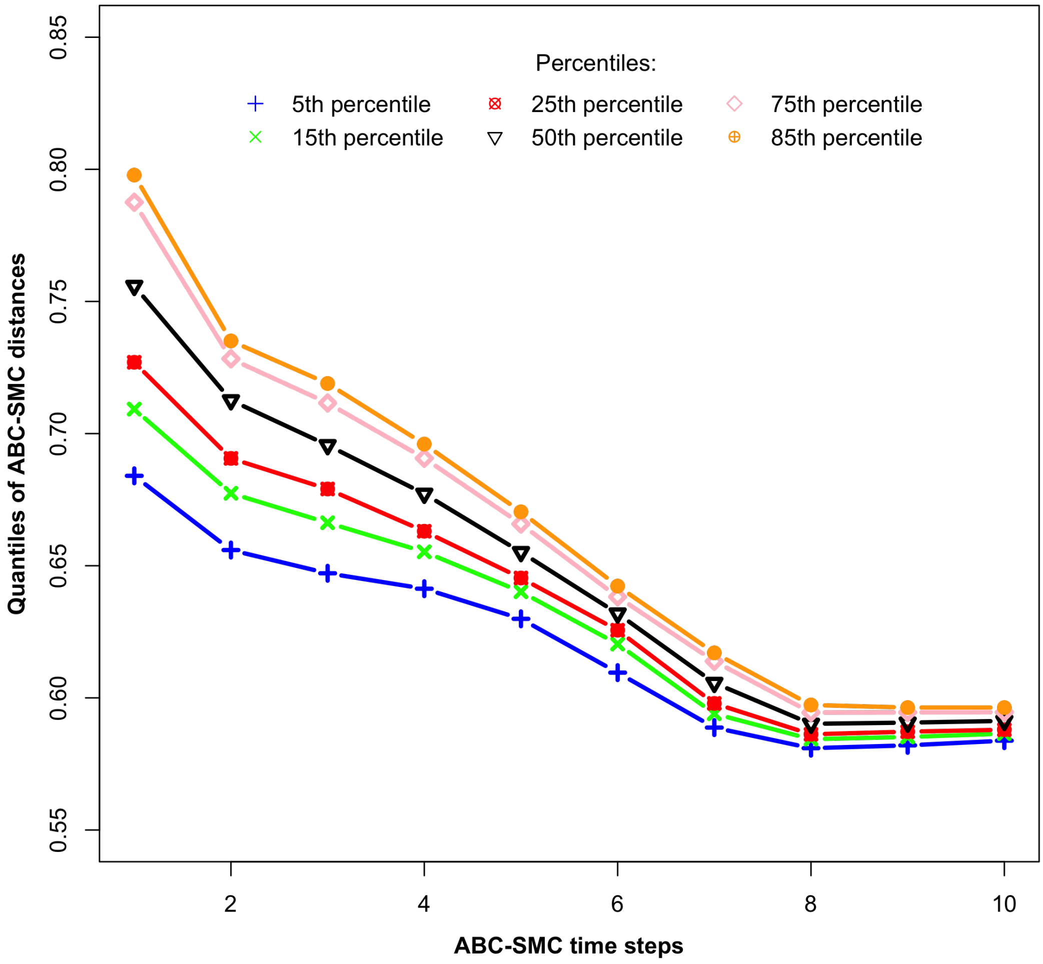

Fig. 15: Plot of distance quantiles at the 10 ABC time steps at N=1000.

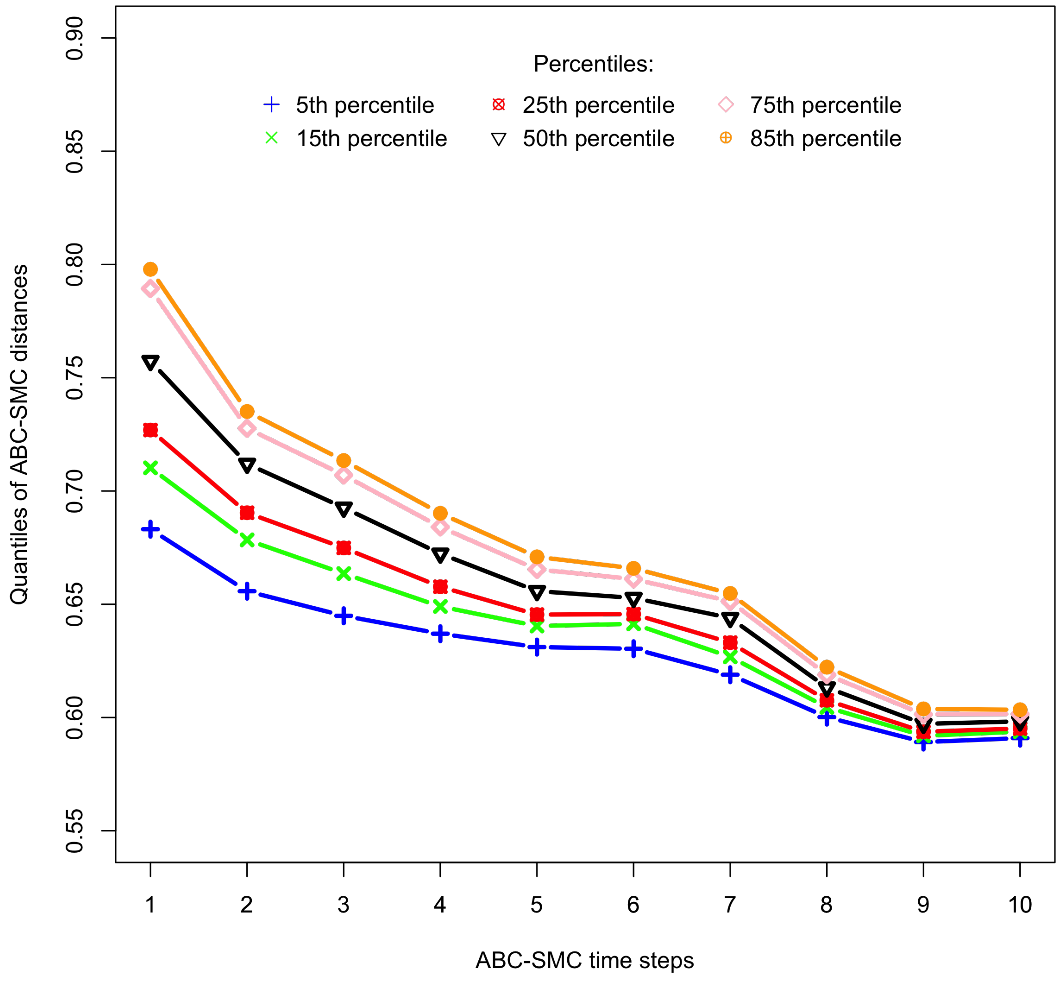

Fig. 16: Plot of distance quantiles at the 10 ABC time steps at N=1500.

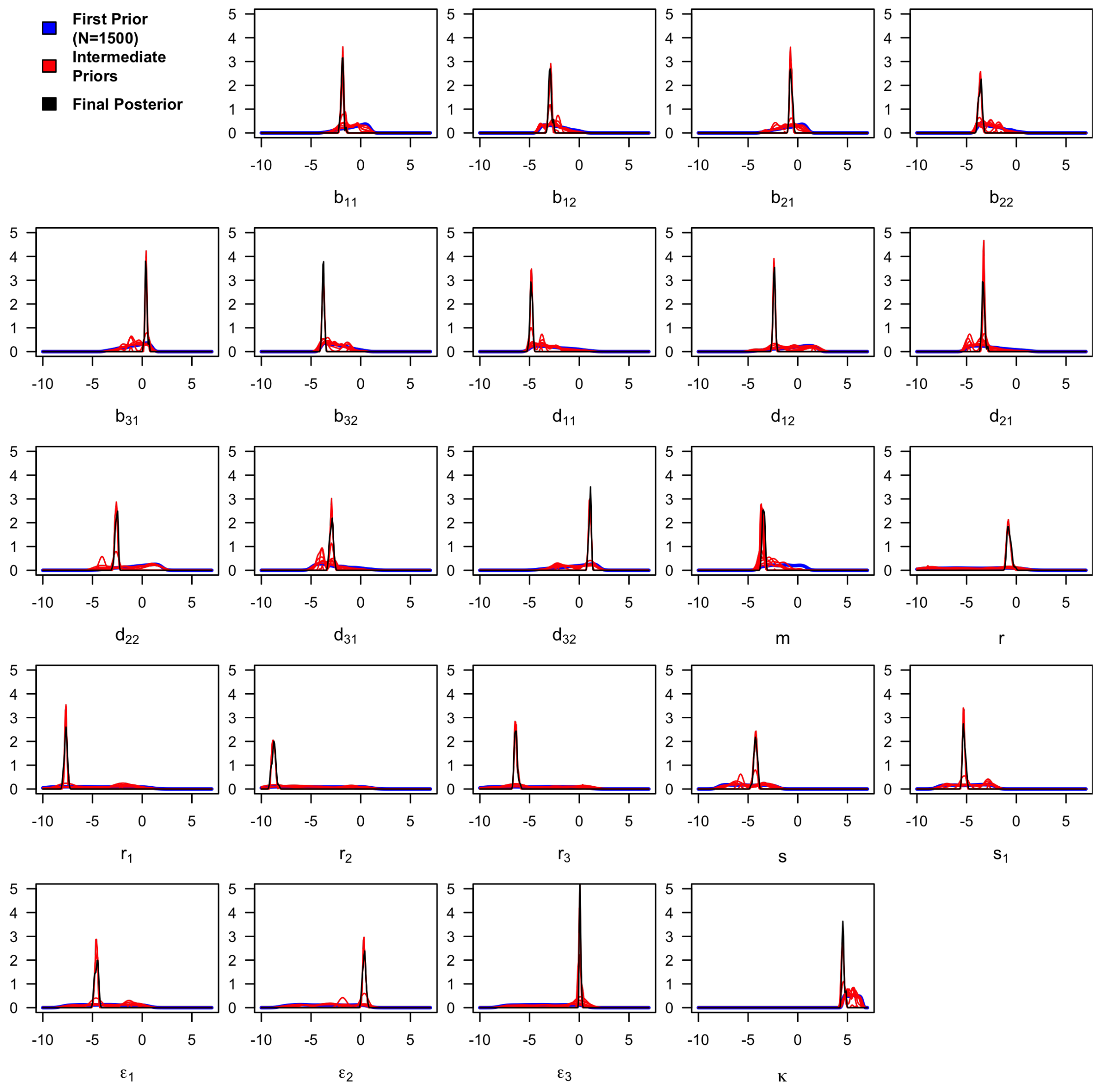

Fig. 17: Marginal density plots of the approximate posterior distributions (in black) for the 23 parameters of the complex stochastic model against the sequentially improving prior distributions at $N = 1500$ (on logarithmic scale).

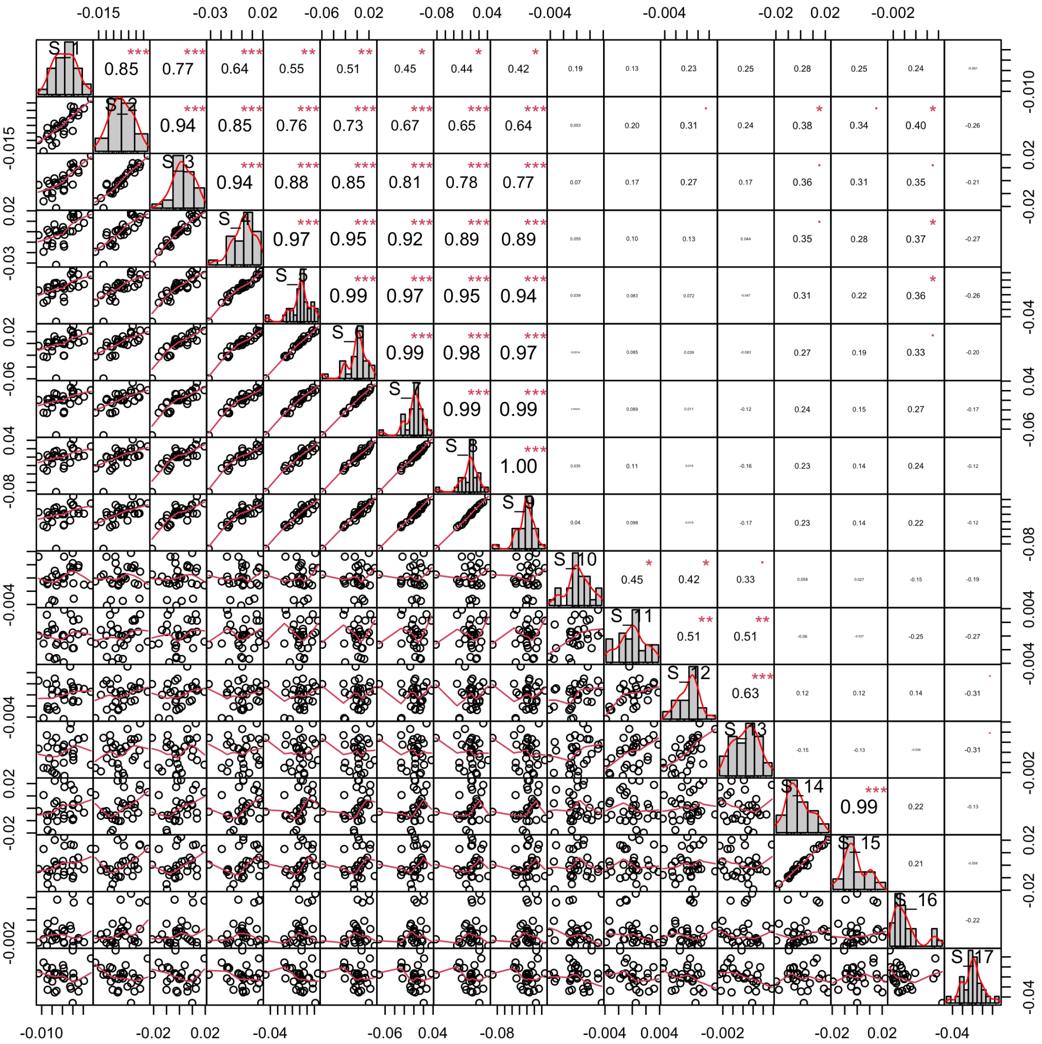

Fig. 18: Correlation matrix plot indicating high multicollinearity between some of the 17 regression predictors (denoted by S_i, $1 \leq i \leq 17$ in the neighbourhood of the pseudo-observed summary statistics) in the modified regression-adjusted ABC (with $L2$ regularisation).

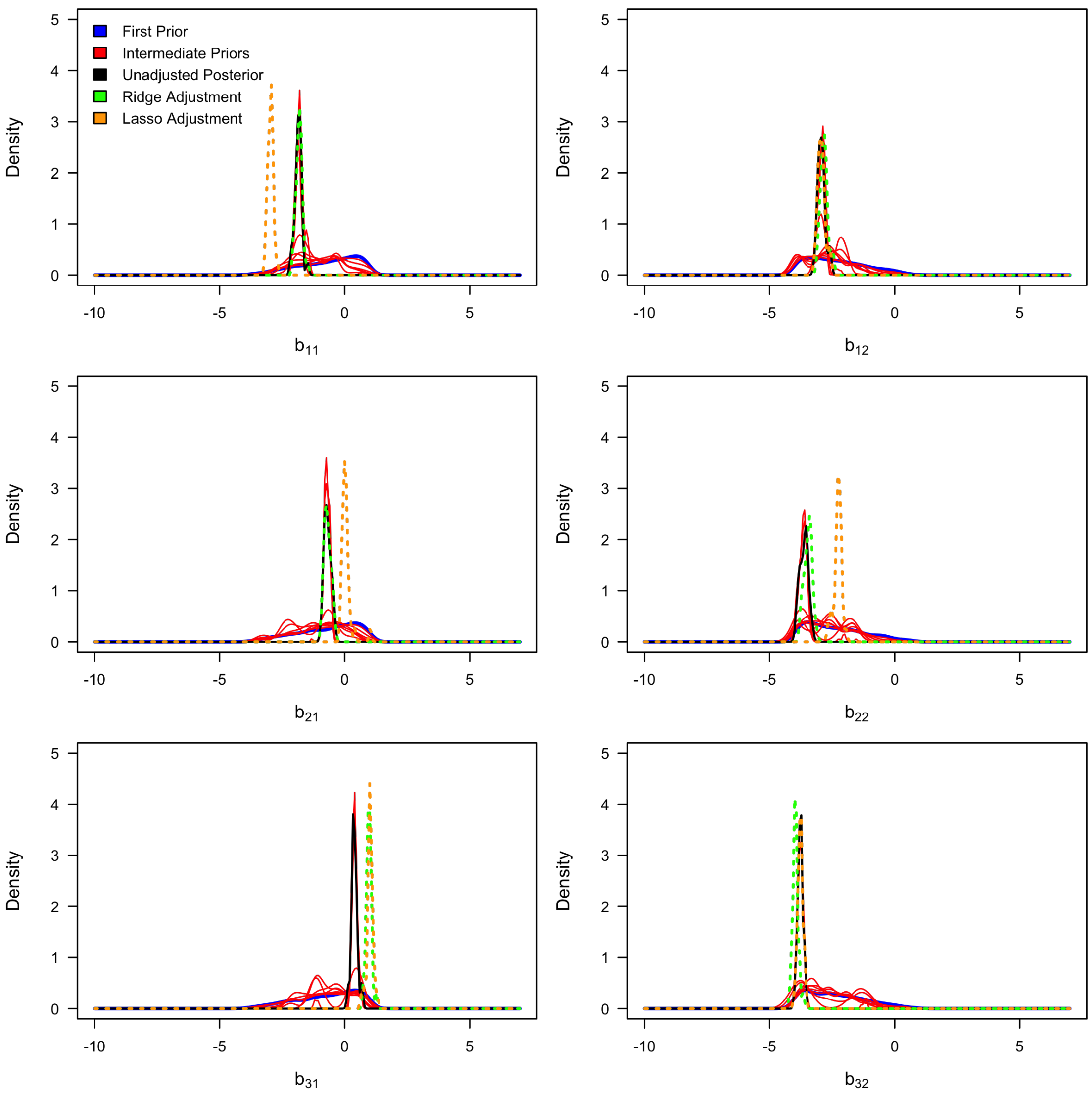

Fig. 19: Marginal density plots of the unadjusted (in black) and adjusted (in green) posterior distributions of model parameters: b_{11}, b_{12}, b_{21}, b_{22}, b_{31}, and b_{32} against the sequentially improving prior distributions (on logarithmic scale) based on pseudo-observed data.

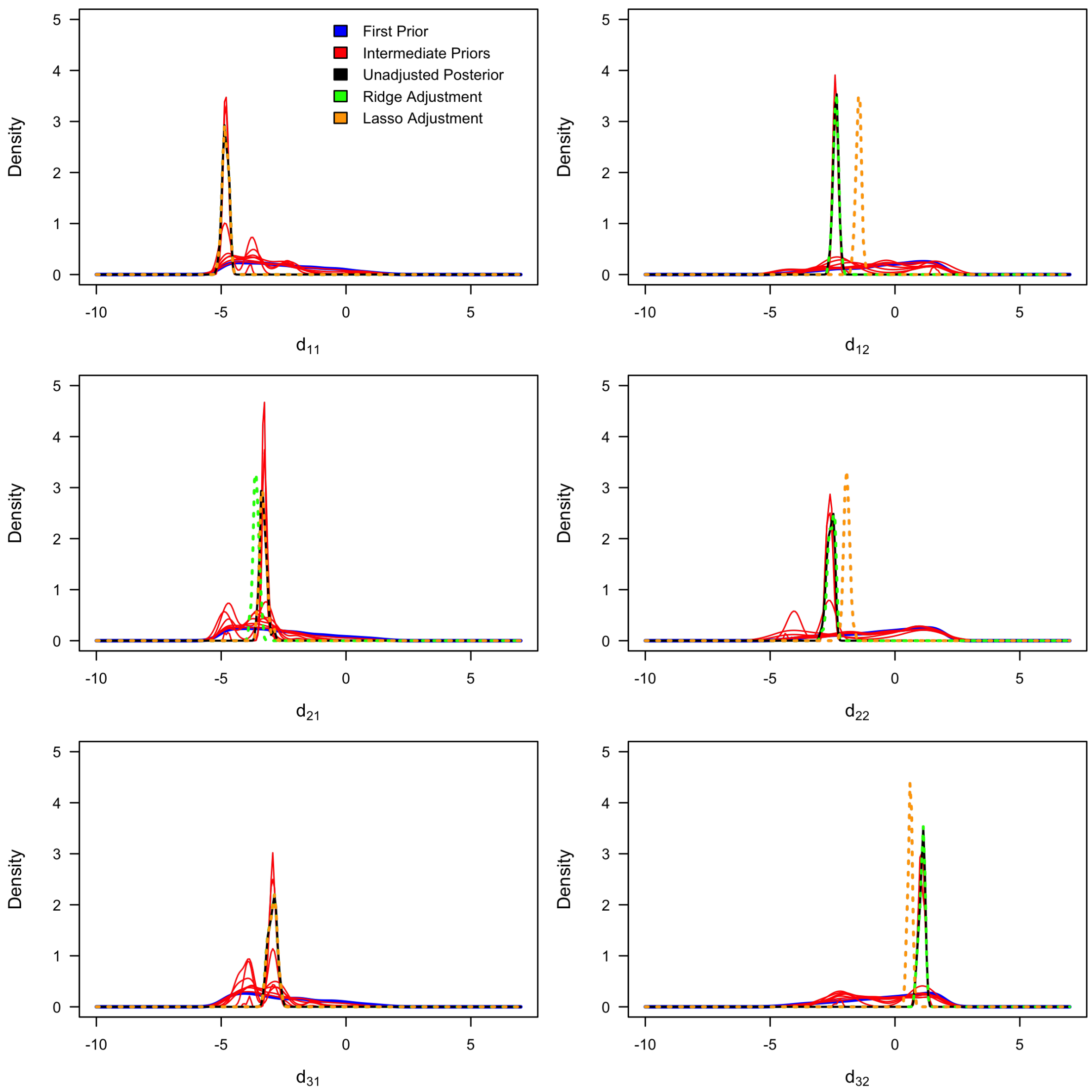

Fig. 20: Marginal density plots of the unadjusted (in black) and adjusted (in green) posterior distributions of model parameters: d_{11}, d_{12}, d_{21}, d_{22}, d_{31}, and d_{32} against the sequentially improving prior distributions (on logarithmic scale) based on pseudo-observed data.

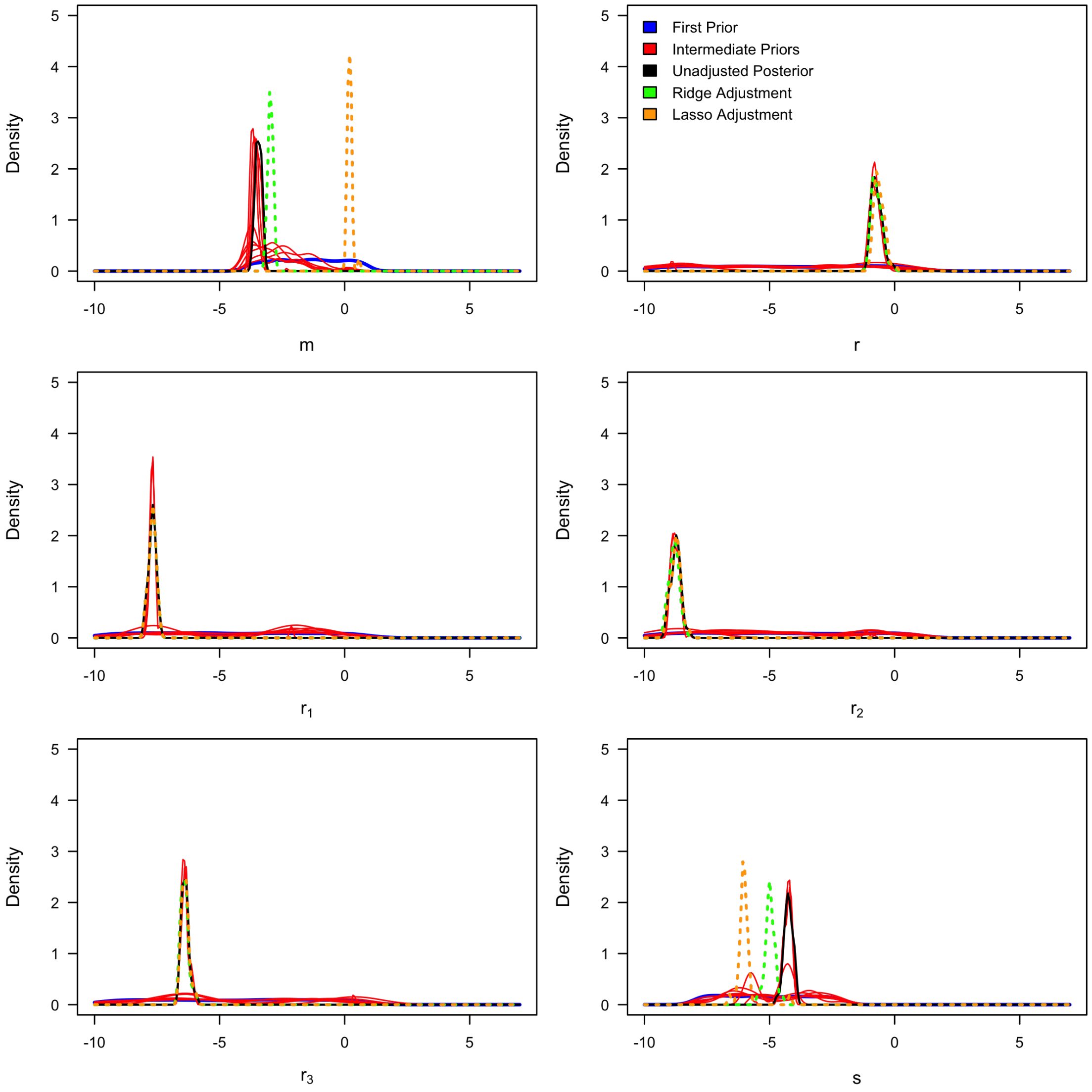

Fig. 21: Marginal density plots of the unadjusted (in black) and adjusted (in green) posterior distributions of model parameters: m, r, r_1, r_2, r_3, and s against the sequentially improving prior distributions (on logarithmic scale) based on pseudo-observed data.

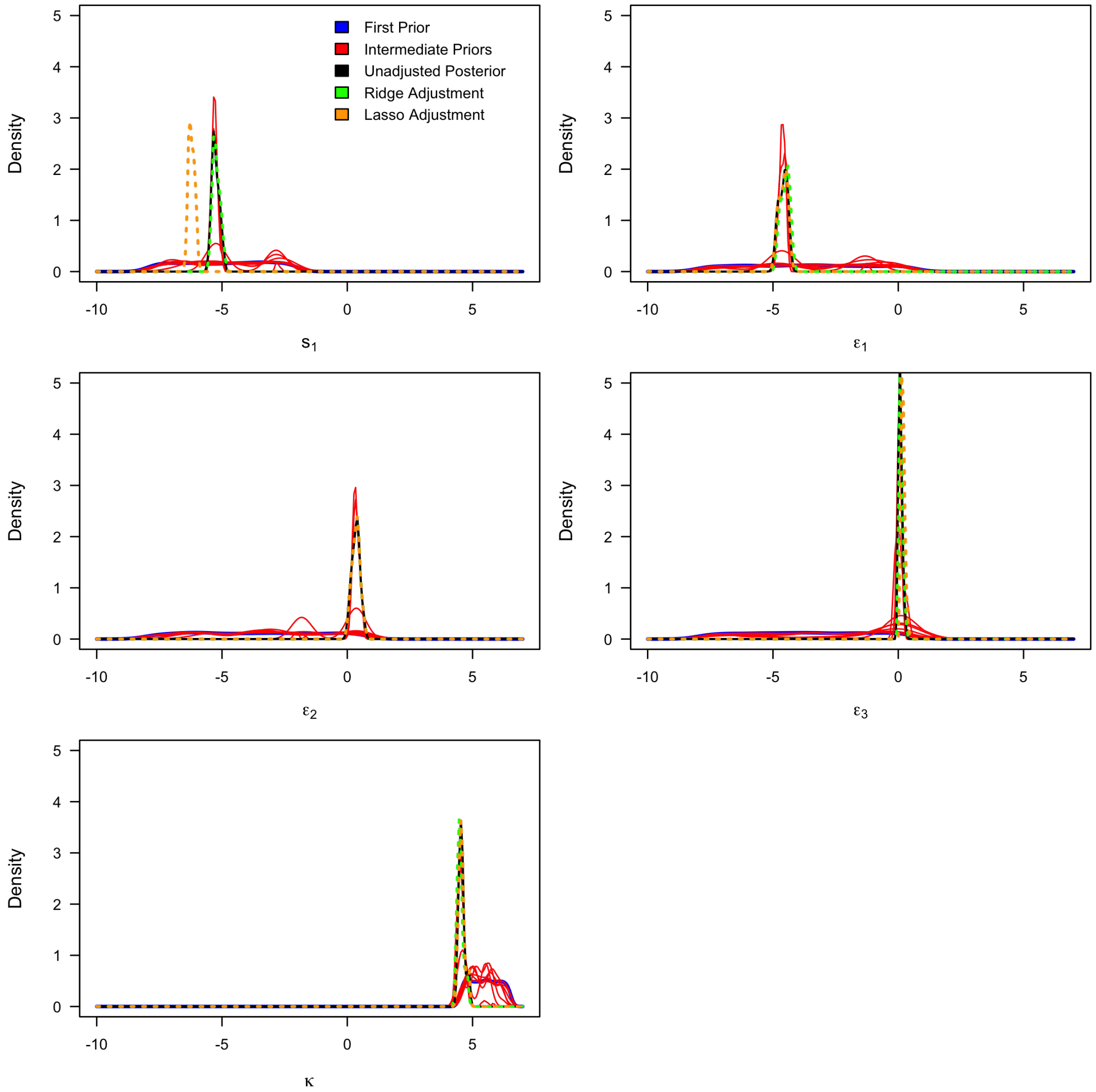

Fig. 22: Marginal density plots of the unadjusted (in black) and adjusted (in green) posterior distributions of model parameters: s_1, ϵ_1, ϵ_2, ϵ_3, and κ against the sequentially improving prior distributions (on logarithmic scale) based on pseudo-observed data.

4.2.2 Assessing the accuracy of the unadjusted and adjusted ABC posterior estimates given pseudo-observed data

Additionally, the bias, variance, and mean square error (MSE) of the posterior mean estimates, as well as their corresponding 95% credible intervals, were estimated to compare the performance of approximations from the weighted-iterative ABC and the two regression adjustments numerically. Tables 2–4 summarise the unadjusted and adjusted posterior estimates, presenting their corresponding 95% credible intervals (Cred. Int) and accuracy measures (bias, variance, and MSE), respectively. Figure 23 provides a graphical summary of the bias of the estimated model parameters based on the unadjusted and regression-adjusted posteriors, along with their pooled estimates of bias, variance, and MSE. The pooled estimates of the accuracy measures were calculated based on the averages across all model parameters concerning each measure. Generally, the unadjusted and ridge-adjusted posterior estimates achieve relatively lower biases and lower MSE compared to that of the lasso-adjusted posterior estimates.

In addition, with the help of the *vegan* R package [3], we performed a multidimensional scaling using Principal Coordinate Analysis (PCoA) to explore and visualise the similarities or dissimilarities (based on the Bray-Curtis distance metric) among posterior samples from the ABC methods under lower-dimensional space (see Part A of Figure 24). It was shown from the PCoA plot that the unadjusted posterior from the weighted iterative ABC and the ridge-adjusted posterior samples are more similar compared to the lasso-adjusted posterior. However, from a multivariate homogeneity test of variances between these comparable groups (p-value = 0.999), we discovered that the variability in the posterior samples between the three ABC posterior approximation methods is statistically insignificant in the multivariate space concerning their posterior samples (based on their respective average distances of the posterior samples to the group posterior centroid or spatial median); see Part B of Figure 24.

Table 2: Comparison between unadjusted ABC posterior mean estimates ($\hat{\boldsymbol{\theta}}_{\text{unadj}}$), and true parameter values ($\hat{\boldsymbol{\theta}}_0$) of the 23 parameters of the stochastic model across at $N = 1500$ (on log scale) with their respective accuracy measures.

Parameters	$\hat{\boldsymbol{\theta}}_{\text{unadj}}$[95% Cred. Int]	$\hat{\boldsymbol{\theta}}_0$	bias($\hat{\boldsymbol{\theta}}_{\text{unadj}}$)[95% Cred. Int]	Var($\hat{\boldsymbol{\theta}}_{\text{unadj}}$)	MSE($\hat{\boldsymbol{\theta}}_{\text{unadj}}$)
**Base simulation parameters**					
b_{11}	-1.8528(-2.1039, -1.6630)	-1.7170	-0.1358(-0.3869, 0.0540)	0.0173	0.0357
b_{12}	-2.9262(-3.1283, -2.5892)	-2.8612	-0.0650(-0.2671, 0.2720)	0.0211	0.0253
b_{21}	-0.6830(-0.8677, -0.4349)	-0.4365	-0.2465(-0.4312, 0.0016)	0.0178	0.0786
b_{22}	-3.6268(-3.8942, -3.3427)	-3.3496	-0.2773 (-0.5447, 0.0069)	0.0261	0.1030
b_{31}	0.3885(0.1621, 0.6682)	0.7021	-0.3136(-0.5400, -0.0339)	0.0140	0.1123
b_{32}	-3.7723(-3.9447, -3.5832)	-3.7423	-0.0300(-0.2024, 0.1591)	0.0103	0.0112
d_{11}	-4.8283(-5.0516, -4.6442)	-5.2214	0.3930(0.1698, 0.5771)	0.0150	0.1694
d_{12}	-2.3719(-2.5716, -2.1820)	-2.3361	-0.0358(-0.2355, 0.1541)	0.0117	0.0130
d_{21}	-3.3159(-3.5486, -3.0352)	-3.4204	0.1044(-0.1282, 0.3852)	0.0198	0.0307
d_{22}	-2.5836(-2.8576, -2.4077)	-2.6451	0.0615(-0.2125, 0.2374)	0.0197	0.0235
d_{31}	2.9412(-3.2046, -2.6463)	-2.9225	-0.0188(-0.2821, 0.2761)	0.0270	0.0274
d_{32}	1.0786(0.8432, 1.2288)	1.3443	-0.2656(-0.5011, -0.1155)	0.0126	0.0831
m	-3.4420(-3.6639, -3.2298)	-2.8404	-0.6016(-0.8235, -0.3893)	0.0154	0.3773
r	-0.7077(-0.9677, -0.2777)	-0.7265	0.0188(-0.2412, 0.4487)	0.0439	0.0443
s	-4.2475(-4.5516, -3.9818)	-4.3200	0.0725(-0.2316, 0.3382)	0.0294	0.0347
κ	4.5693(4.3934, 4.8498)	4.7715	-0.2021(-0.3781, 0.0783)	0.0153	0.0561
**Additional simulation parameters**					
ϵ_1	-4.5952(-4.8778, -4.3371)	-5.1328	0.5376(0.2550, 0.7957)	0.0296	0.3186
ϵ_2	0.3438(0.0692, 0.6367)	0.3460	-0.0022(-0.2768, 0.2907)	0.0250	0.0250
ϵ_3	0.0863(-0.0056, 0.2324)	0.0564	0.0299(-0.0620, 0.1760)	0.0050	0.0059
r_1	-7.6009(-7.9766, -7.4333)	-7.6009	0.0000(-0.3757, 0.1676)	0.0219	0.0219
r_2	-8.5172(-9.0845, -8.4013)	-8.5172	0.0000(-0.5673, 0.1159)	0.0406	0.0406
r_3	-6.3771(-6.5937, -6.0364)	-6.4378	0.0606(-0.1560, 0.4014)	0.0237	0.0274
s_1	-5.2591(-5.4788, -4.9702)	-5.5994	0.3403(0.1206, 0.6292)	0.0205	0.1363

Table 3: Comparison between ridge-adjusted ABC posterior mean estimates ($\hat{\boldsymbol{\theta}}_{\text{ridge}}$), and true parameter values ($\hat{\boldsymbol{\theta}}_0$) of the 23 parameters of the stochastic model across at $N = 1500$ (on log scale) with their respective accuracy measures.

Parameters	$\hat{\boldsymbol{\theta}}_{\text{ridge}}$[95% Cred. Int]	$\hat{\boldsymbol{\theta}}_0$	bias($\hat{\boldsymbol{\theta}}_{\text{ridge}}$)[95% Cred. Int]	Var($\hat{\boldsymbol{\theta}}_{\text{ridge}}$)	MSE($\hat{\boldsymbol{\theta}}_{\text{ridge}}$)
**Base simulation parameters**					
b_{11}	-1.8258(-2.0252, -1.5836)	-1.7170	-0.1088(-0.3082, 0.1334)	0.0154	0.0272
b_{12}	-2.8117(-3.0613, -2.4886)	-2.8612	0.0495(-0.2001, 0.3726)	0.0212	0.0237
b_{21}	-0.6762(-0.8674, -0.4351)	-0.4365	-0.2397(-0.4309, 0.0013)	0.0178	0.0753
b_{22}	-3.4428(-3.7274, -3.1933)	-3.3496	-0.0932(-0.3778, 0.1562)	0.0240	0.0327
b_{31}	0.9656(0.7700, 1.1354)	0.7021	0.2635 (0.0679, 0.4333)	0.0101	0.0795
b_{32}	-3.9772(-4.1425, -3.7449)	-3.7423	-0.2349(-0.4002, -0.0026)	0.0121	0.0673
d_{11}	-4.8208(-5.0515, -4.6442)	-5.2214	0.4005(0.1698, 0.5771)	0.0150	0.1754
d_{12}	-2.3764(-2.5718, -2.1822)	-2.3361	-0.0402(-0.2357, 0.1539)	0.0117	0.0133
d_{21}	-3.6033(-3.8360, -3.3863)	-3.4204	-0.1830(-0.4157, 0.0340)	0.0131	0.0466
d_{22}	-2.5445(-2.7766, -2.3650)	-2.6451	0.1006(-0.1315, 0.2801)	0.0185	0.0286
d_{31}	-2.9419(-3.2125, -2.6565)	-2.9225	-0.0194(-0.2900, 0.2660)	0.0270	0.0274
d_{32}	1.0752(0.8430, 1.2291)	1.3443	-0.2691(-0.5013, -0.1151)	0.0126	0.0850
m	-2.9674(-3.1613, -2.8082)	-2.8404	-0.1269(-0.3208, 0.0322)	0.0101	0.0262
r	-0.7337(-0.9698, -0.2796)	-0.7265	-0.0072(-0.2433, 0.4469)	0.0438	0.0439
s	-4.9887(-5.3812, -4.6765)	-4.3200	-0.6687(-1.0612, -0.3565)	0.0371	0.4843
κ	4.5072(4.3450, 4.7899)	4.7715	-0.2643(-0.4265, 0.0184)	0.0145	0.0844
**Additional simulation parameters**					
ϵ_1	-4.5126(-4.8037, -4.2531)	-5.1328	0.6202(0.3291, 0.8797)	0.0295	0.4141
ϵ_2	0.3646(0.0703, 0.6375)	0.3460	0.0186(-0.2757, 0.2915)	0.0250	0.0253
ϵ_3	0.1154 (0.0072, 0.2529)	0.0564	0.0590(-0.0492, 0.1965)	0.0050	0.0085
r_1	-7.6840(-7.9782, -7.4346)	-7.6009	-0.0830(-0.3773, 0.1663)	0.0219	0.0288
r_2	-8.8284(-9.1605, -8.4888)	-8.5172	-0.3112(-0.6433, 0.0284)	0.0382	0.1350
r_3	-6.4305(-6.6175, -6.0519)	-6.4378	0.0072(-0.1797, 0.3859)	0.0239	0.0240
s_1	-5.2316(-5.4495, -4.9453)	-5.5994	0.3679(0.1499, 0.6541)	0.0200	0.1554

Table 4: Comparison between lasso-adjusted ABC posterior mean estimates ($\hat{\boldsymbol{\theta}}_{\text{lasso}}$), and true parameter values ($\hat{\boldsymbol{\theta}}_0$) of the 23 parameters of the stochastic model across at $N = 1500$ (on log scale) with their respective accuracy measures.

Parameters	$\hat{\boldsymbol{\theta}}_{\text{lasso}}$[95% Cred. Int]	$\hat{\boldsymbol{\theta}}_0$	bias($\hat{\boldsymbol{\theta}}_{\text{lasso}}$)[95% Cred. Int]	Var($\hat{\boldsymbol{\theta}}_{\text{lasso}}$)	MSE($\hat{\boldsymbol{\theta}}_{\text{lasso}}$)
**Base simulation parameters**					
b_{11}	-2.9691(-3.1256,-2.7833)	-1.7170	-1.2521 (-1.4086, -1.0663)	0.0103	1.5781
b_{12}	-2.9234(-3.1283, -2.5892)	-2.8612	-0.0622 (-0.2671, 0.2720)	0.0211	0.0250
b_{21}	0.0134(-0.1308, 0.5794)	-0.4365	0.4499(0.3057, 1.0159)	0.0465	0.2489
b_{22}	-2.2381(-2.6092, -2.0622)	-3.3496	1.1114 (0.7403, 1.2874)	0.0227	1.2579
b_{31}	1.0168(0.7547, 1.2066)	0.7021	0.3147(0.0526, 0.5045)	0.0139	0.1129
b_{32}	-3.7752(-3.9452, -3.5834)	-3.7423	-0.0329(-0.2029, 0.1589)	0.0104	0.0115
d_{11}	-4.8212(-5.0516, -4.6443)	-5.2214	0.4001(0.1698, 0.5771)	0.0150	0.1751
d_{12}	-1.4549(-1.6509, -1.2980)	-2.3361	0.8812(0.6853, 1.0381)	0.0106	0.7871
d_{21}	-3.3026(-3.5485, -3.0351)	-3.4204	0.1177(-0.1281, 0.3852)	0.0198	0.0337
d_{22}	-1.9336(-2.1118, -1.7157)	-2.6451	0.7115(0.5333, 0.9293)	0.0127	0.5189
d_{31}	-2.9332(-3.2045, -2.6462)	-2.9225	-0.0107(-0.2820, 0.2763)	0.0270	0.0271
d_{32}	0.5944(0.4450, 0.7349)	1.3443	-0.7499(-0.8993, -0.6094)	0.0071	0.5695
m	0.1914(0.0820, 0.3858)	-2.8404	3.0318(2.9225, 3.2262)	0.0092	9.2010
r	-0.6332(-0.8639, -0.2320)	-0.7265	0.0933(-0.1374, 0.4945)	0.0408	0.0495
s	-6.0259(-6.3850, -5.3355)	-4.3200	-1.7060(-2.0650, -1.0155)	0.1409	3.0513
κ	4.5670(4.3940, 4.8504)	4.7715	-0.2044(-0.3774, 0.0789)	0.0153	0.0571
**Additional simulation parameters**					
ϵ_1	-4.6071(-4.8765, -4.3359)	-5.1328	0.5257(0.2563, 0.7969)	0.0296	0.3060
ϵ_2	0.3710(0.0692, 0.6367)	0.3460	0.0250(-0.2768, 0.2907)	0.0250	0.0256
ϵ_3	0.1947(0.0968, 0.3326)	0.0564	0.1383(0.0404, 0.2762)	0.0049	0.0240
r_1	-7.6898(-7.9766, -7.4333)	-7.6009	-0.0889(-0.3757, 0.1676)	0.0219	0.0298
r_2	-8.7544(-9.0845, -8.4013)	-8.5172	-0.2372(-0.5673, 0.1159)	0.0406	0.0969
r_3	-6.4076(-6.5935, -6.0363)	-6.4378	0.0301(-0.1558, 0.4015)	0.0237	0.0246
s_1	-6.2127(-6.3759, -6.0130)	-5.5994	-0.6133(-0.7765, -0.4135)	0.0132	0.3893

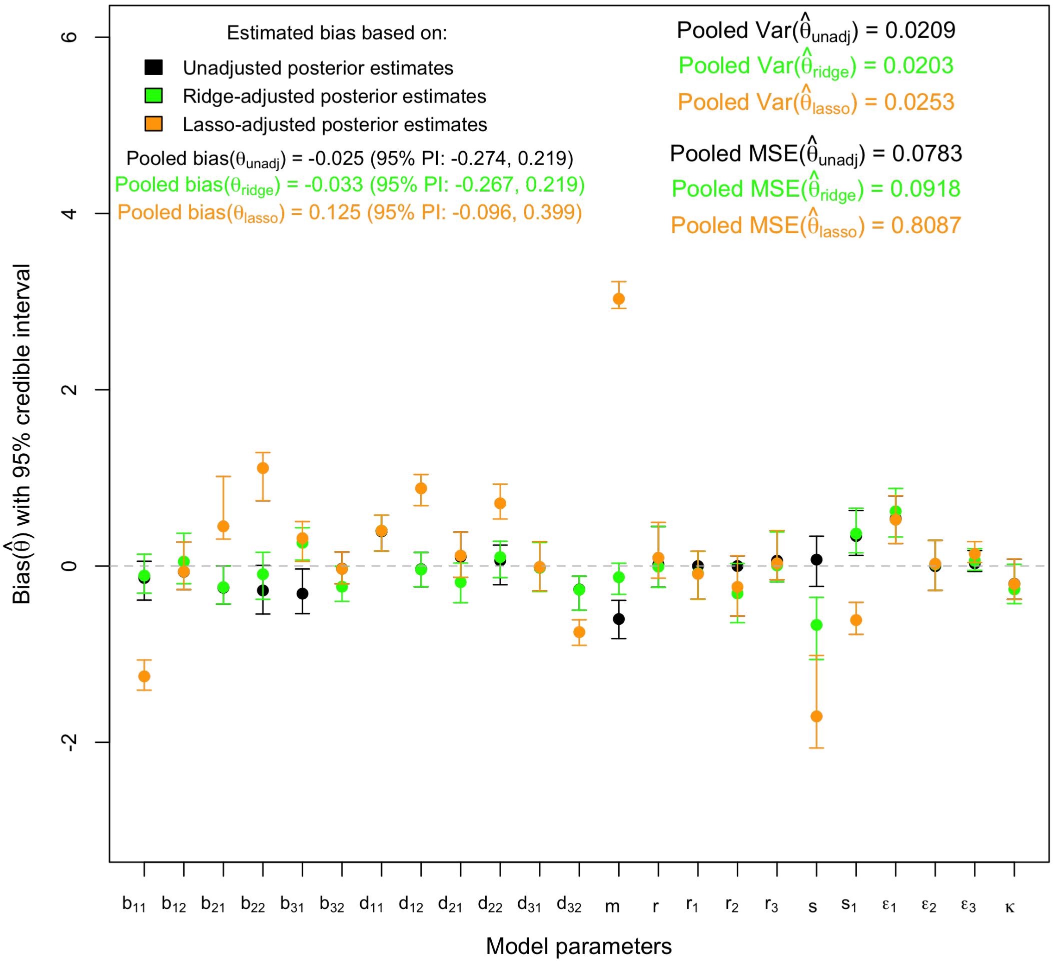

Fig. 23: Comparative plot of the bias of the estimated model parameters based on the unadjusted and regression-adjusted posteriors with their pooled estimates of the bias, variance and MSE.

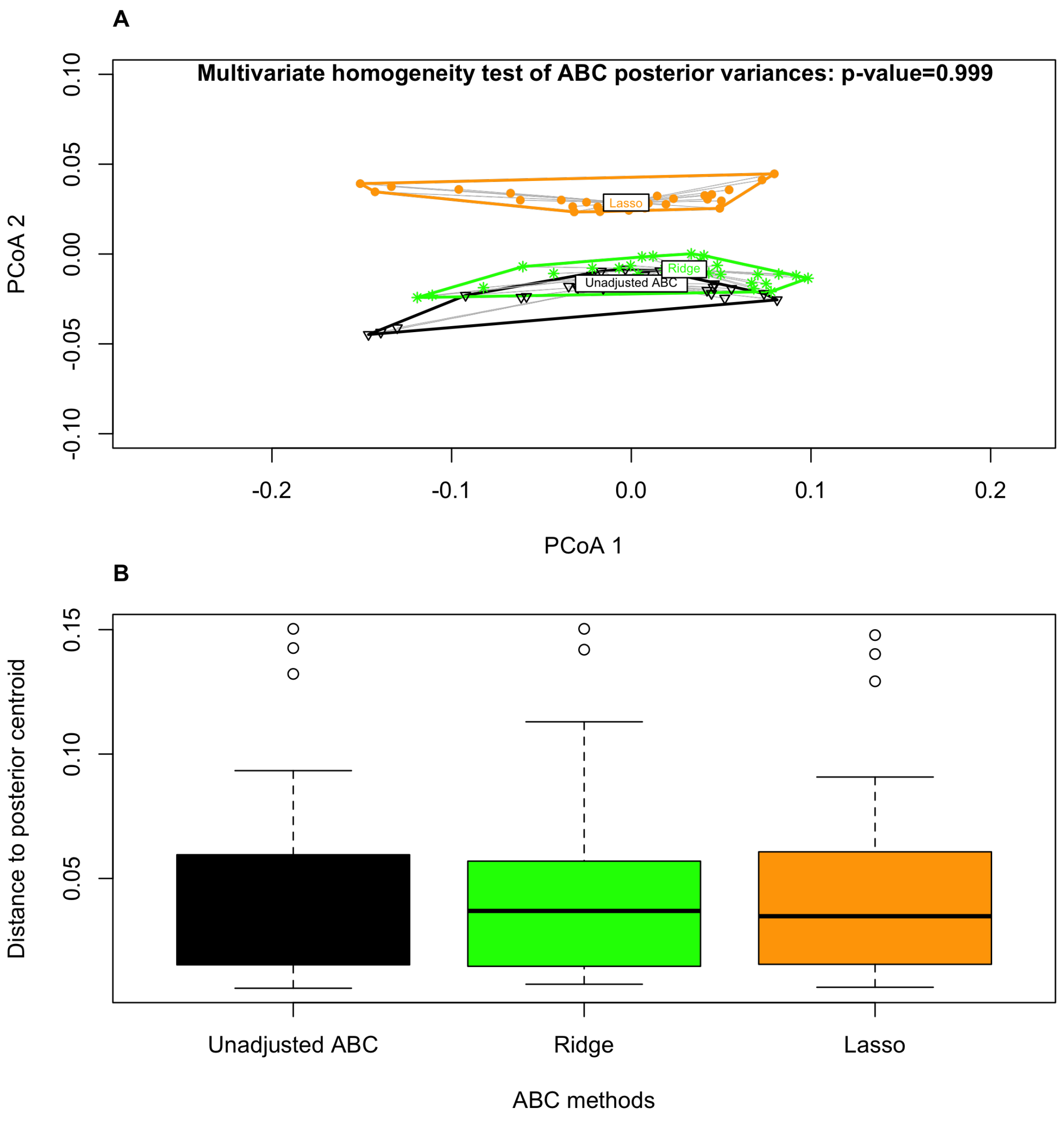

Fig. 24: PCoA plot of the similarities between posterior samples under a lower-dimensional space (Part A) and the distribution of the average distances of the posterior samples to their posterior centriod (Part B) between the unadjusted ABC and the two penalised regression-adjusted ABC methods (based on ridge and lasso regularisations).

4.2.3 Multivariate posterior predictive analysis using PCA given pseudo-observed data

We employed principal component analysis (PCA) to examine further the distribution of simulations derived from the posterior approximations and to evaluate the unadjusted and the two regression-adjusted posteriors. Specifically, our analysis explored potential patterns between the high-dimensional pseudo-observed data and simulations based on unadjusted, ridge-adjusted, and lasso-adjusted posterior within a lower-dimensional space. Part A of Figure 25 provides a graphical summary of the squared cosines of contributions (Cos2) arranged in decreasing order of importance. This arrangement offers insights into the contribution of each data variable (i.e., parasite data at each observed time point) to the principal components. Additionally, Part B of Figure 25 represents a PCA-biplot describing the structure of multivariate data and illustrating the relationships between the data variables.

The PCA plot presented in Figure 26 illustrates the variability and hierarchical relationship between pseudo-observed and simulated data within a reduced-dimensional space. Simulations derived from the unadjusted posterior exhibit spatial concentration within the pseudo-observed data. In contrast, the pseudo-observed data aligns more closely with simulations based on the ridge-adjusted posterior, and the latter set is contained within simulations derived from the lasso-adjusted posterior. This observation implies that the simulated data from the ridge-adjusted posterior are either much closer to the pseudo-observed data or exhibit lower discrepancy compared to simulations derived from the lasso-adjusted posterior. Meanwhile, simulations from the unadjusted posterior appear to underestimate the pseudo-observed data, a conclusion supported by the comparison of parasite mean intensities, as depicted in Figure 27. Figure 28 confirms that there is no statistical difference between the pseudo-observed data and the simulated data derived from the ridge-adjusted posterior concerning the distribution of parasites across the observation time points. Therefore, the posterior correction method utilising ridge adjustment exhibited a superior model fit compared to the lasso adjustment method, thereby enhancing the unadjusted posterior. However, it is essential to note that this conclusion may or may not consistently align with

the actual observed data during the ABC fitting process.

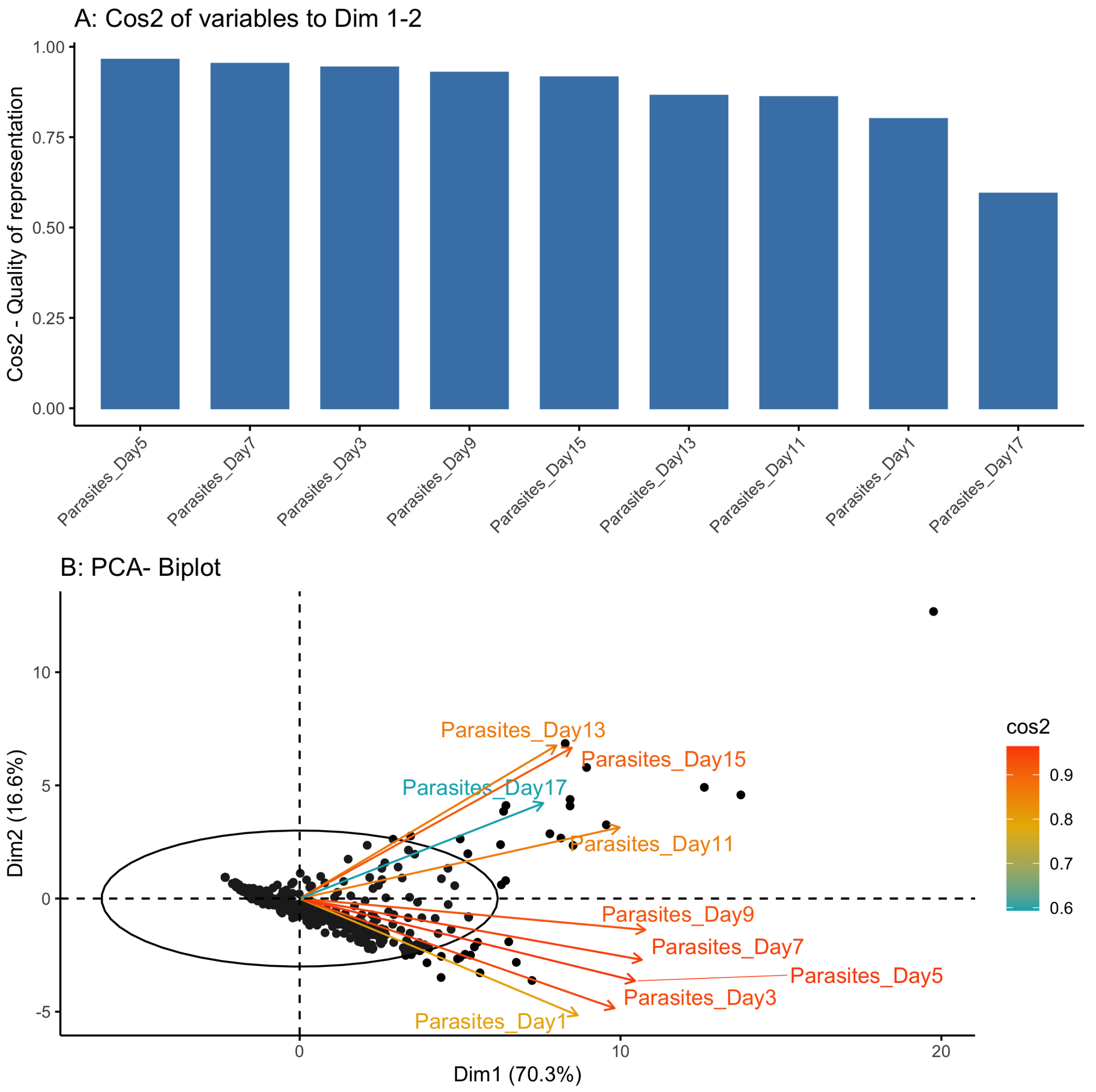

Fig. 25: Quality of data representation on the PCA factor map (Part A) and PCA biplot (Part B).

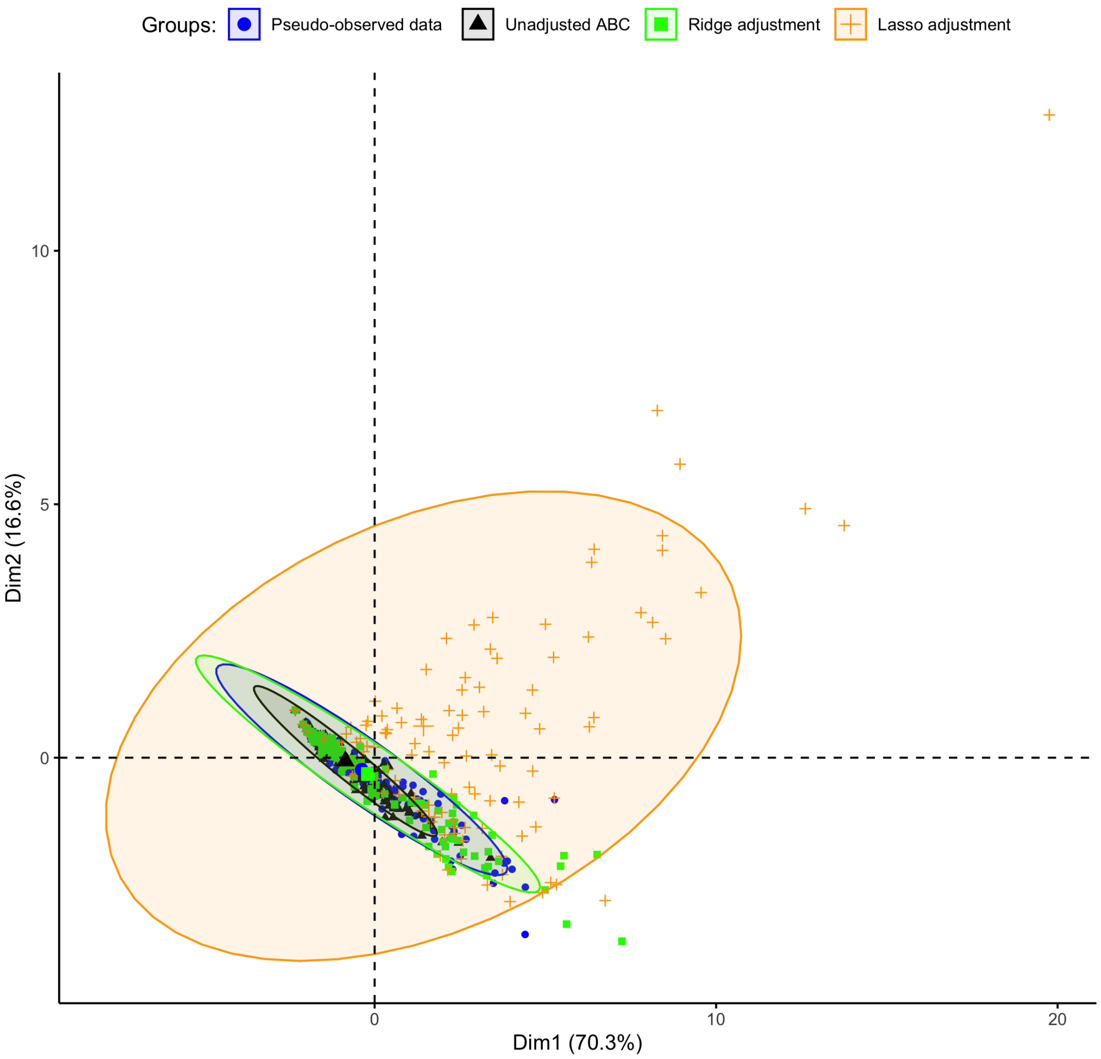

Fig. 26: PCA plot describing the variability and hierarchical relationship between the pseudo-observed and simulated data (based on the unadjusted and regression-adjusted posterior estimates) within a lower dimensional space.

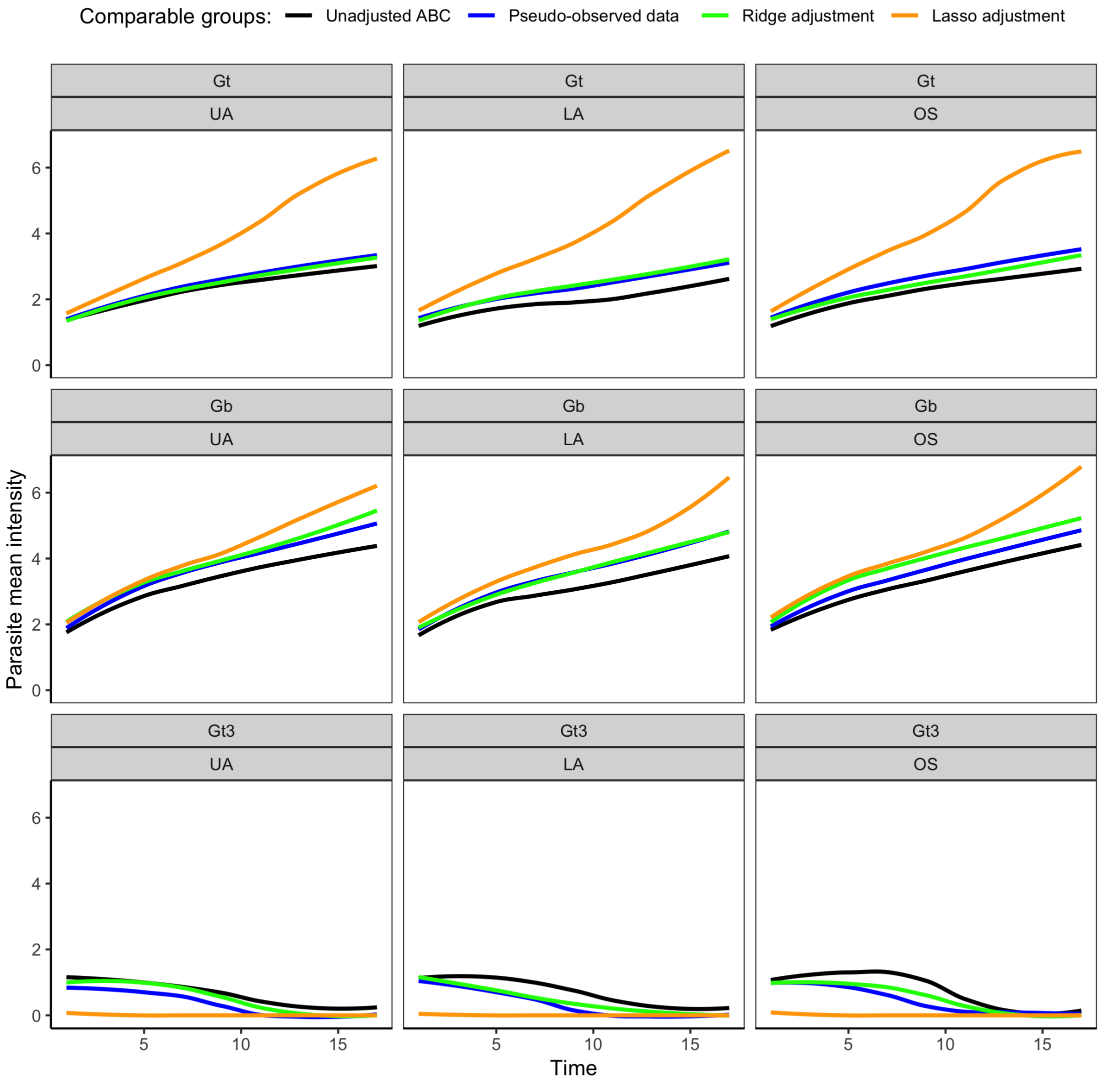

Fig. 27: Parasite mean intensities across parasite strain and fish stock stratified by the four comparable groups.

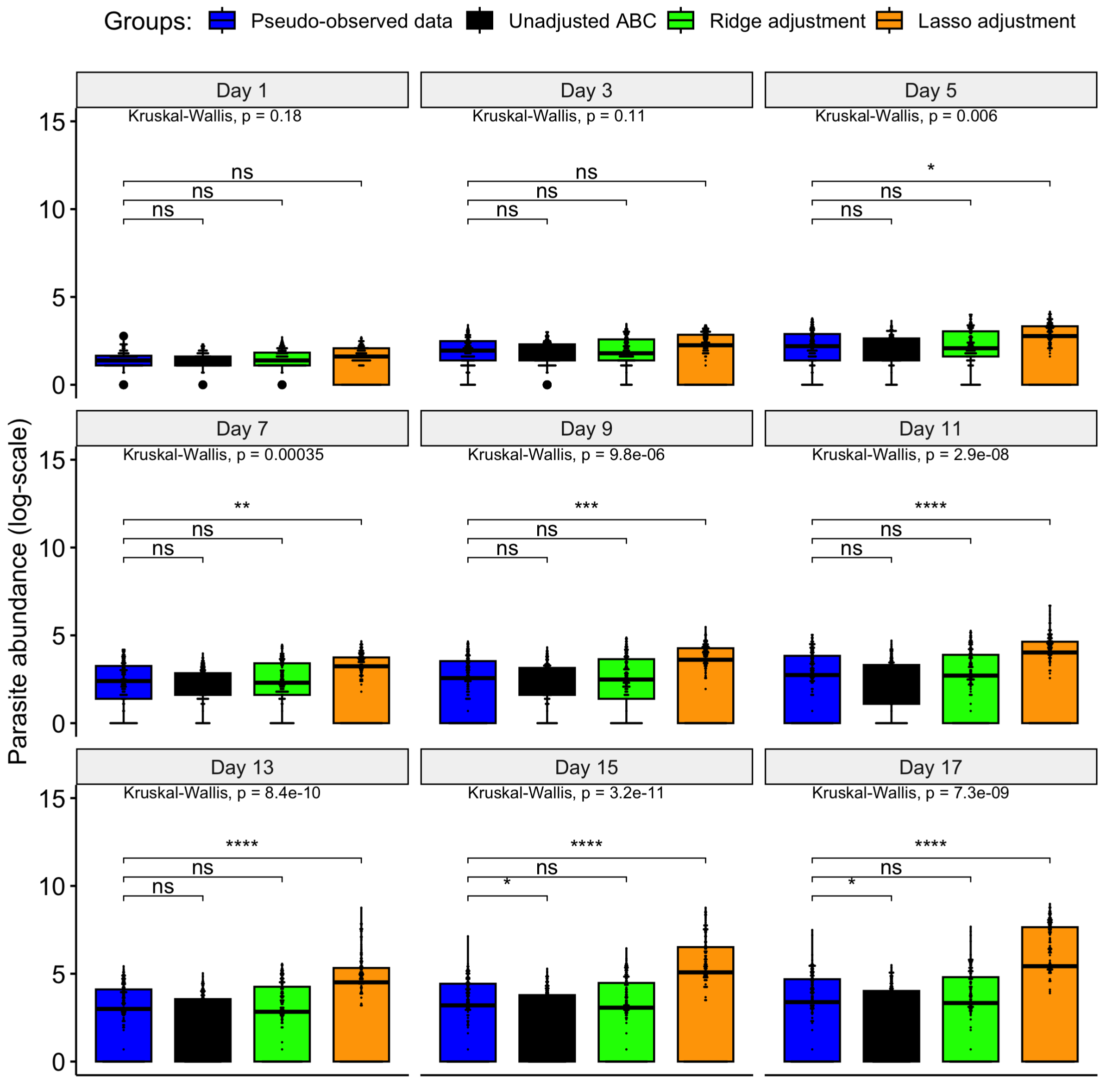

Fig. 28: Comparing the distribution of the pseudo-observed data and the different simulated data (where ^{ns}p-value non-significant; * p< 0.05; ** p< 0.01; *** p< 0.001; **** p< 0.0001).

5 Supplementary Figures: Unadjusted ABC marginal density plots at $N = 500$, 1000 and 1500 given the actual observed data

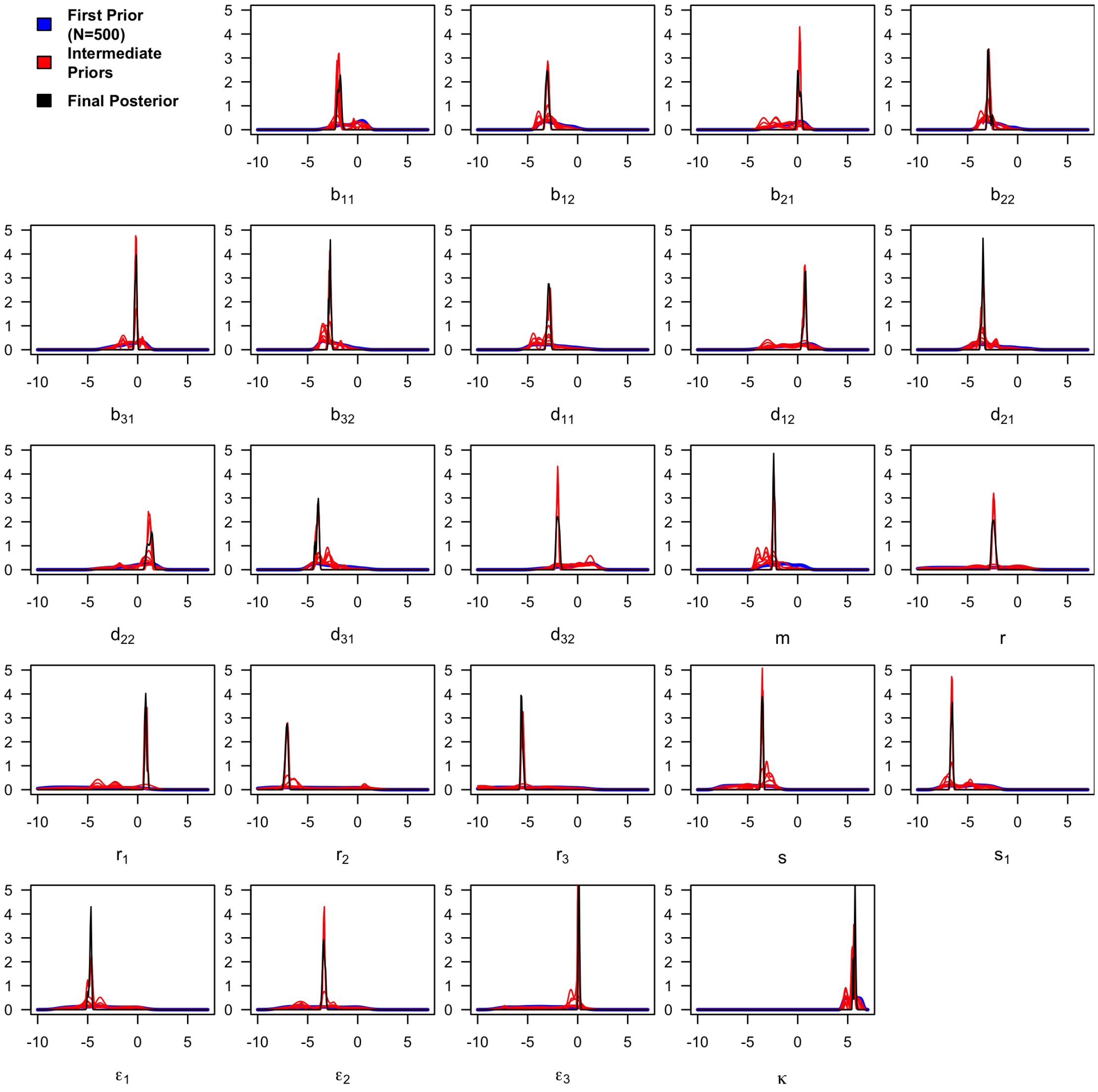

Fig. 29: Marginal density plots of the unadjusted approximate posterior distributions (in black) for the 23 parameters of the complex stochastic model against the sequentially improving prior distributions at $N = 500$ (on logarithmic scale).

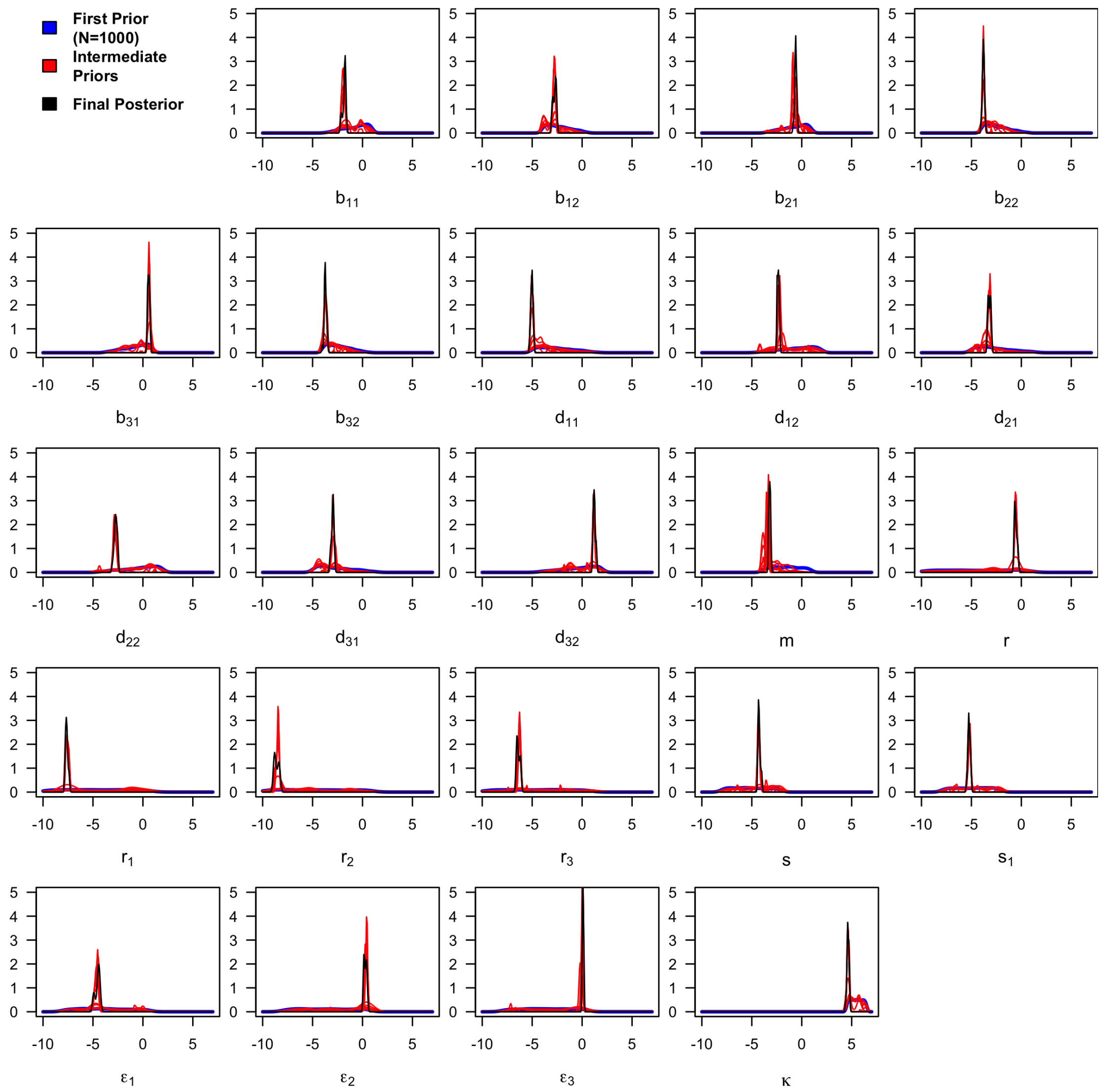

Fig. 30: Marginal density plots of the unadjusted approximate posterior distributions (in black) for the 23 parameters of the complex stochastic model against the sequentially improving prior distributions at $N = 1000$ (on logarithmic scale).

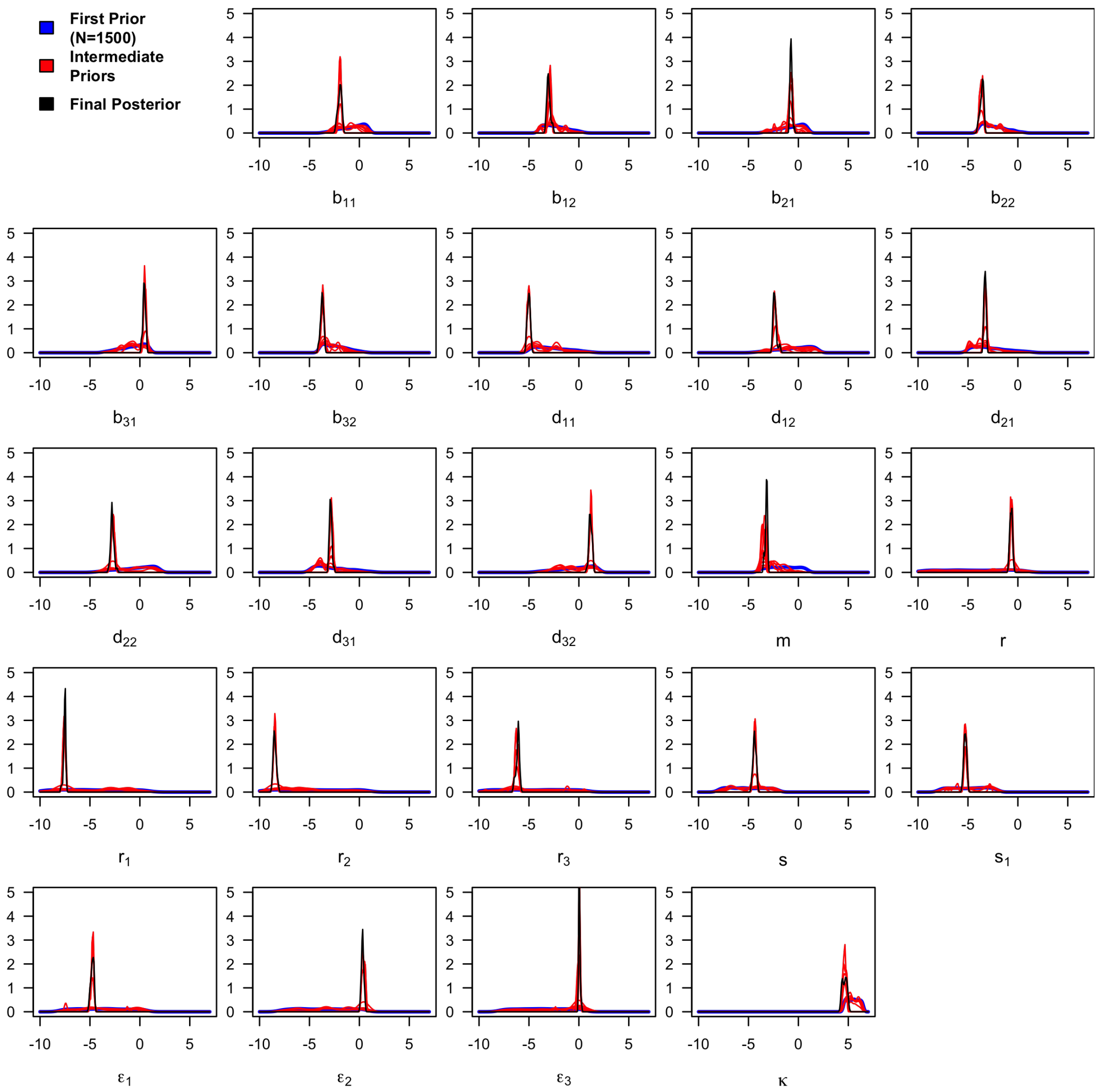

Fig. 31: Marginal density plot of the unadjusted approximate posterior distributions (in black) for the 23 parameters of the complex stochastic model against the sequentially improving prior distributions at $N = 1500$ (on logarithmic scale).

References

[1] Beaumont, M. A., Zhang, W., and Balding, D. J. (2002). Approximate Bayesian computation in population genetics. *Genetics*, 162(4):2025–2035.

[2] Cable, J. and Harris, P. (2002). Gyrodactylid developmental biology: historical

review, current status and future trends. *International Journal for Parasitology*, 32(3):255–280.

[3] Oksanen, J., Blanchet, F. G., Friendly, M., Kindt, R., Legendre, P., McGlinn, D., Minchin, P. R., O'hara, R., Simpson, G. L., Solymos, P., et al. (2019). Package 'vegan'. *Community ecology package, version*, 2(9).

[4] Twumasi, C. (2022). *In silico modelling of parasite dynamics.* PhD thesis, Cardiff University.
